# Direct α-heteroarylation of amides (α to nitrogen) and ethers through a benzaldehyde-mediated photoredox reaction†
†Electronic supplementary information (ESI) available: Fig. S1–S4, Scheme S1–S3, experimental procedures and characterization data for all new compounds. See DOI: 10.1039/c5sc03640b


**DOI:** 10.1039/c5sc03640b

**Published:** 2015-12-07

**Authors:** Yongqiang Zhang, Kevin B. Teuscher, Haitao Ji

**Affiliations:** a Department of Chemistry , Center for Cell and Genome Science , University of Utah , Salt Lake City , Utah 84112-0850 , USA . Email: markji@chem.Utah.edu

## Abstract

A photoredox reaction for cross-dehydrogenative coupling (CDC) was developed to C_α_-arylate amides (α to nitrogen) and ethers using a variety of five- and six-membered electron-deficient heteroarenes. A unique decomposition mechanism of ammonium persulfate enhanced by photoexcited benzaldehydes was revealed. This benzaldehyde-mediated photoredox reaction proceeded smoothly with household 23 W CFL bulbs as the energy source under metal-free conditions, allowing the construction of new C_sp^2^_–C_sp^2^_ and C_sp^3^_–C_sp^2^_ bonds and access to important pharmacophores of broad utility using commercially available reagents.

## Abstract 2

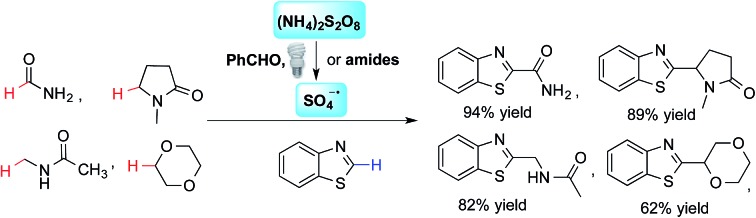
A benzaldehyde-mediated photoredox reaction for the α-heteroarylation of amides (α to nitrogen) and ethers through cross-dehydrogenative coupling (CDC).

## Introduction

Coupling reactions that connect nitrogen-containing heteroaromatic moieties to other molecular structures are a mainstay transformation in the synthesis of medicinal agents. The C–H bonds α to nitrogen or oxygen atoms in amines, amides (α to nitrogen), and ethers can be activated through a single electron transfer (SET) process to generate the corresponding radicals.^1^ This strategy enables a direct conversion of feedstock amines, amides and ethers, *etc.* into α-heteroarylated products that become important and broadly employed pharmacophores for drug discovery (see Fig. S1†).^2^ However, the generation of such radicals and the subsequent coupling reactions require harsh conditions, such as high temperatures, stoichiometric amounts of oxidants, and the use of transition-metal catalysts.^1^

**Fig. 1:**
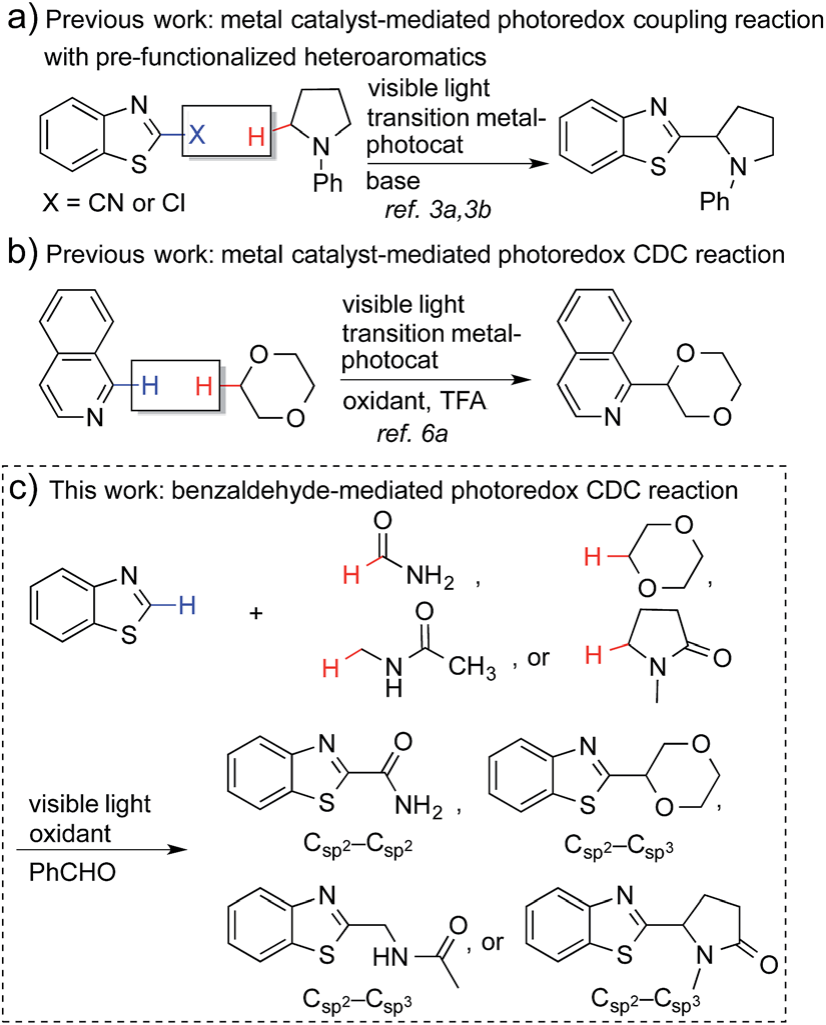
Photoredox-based coupling reactions.

Recently, metal-based, visible light-driven photoredox catalysis has been identified as a suitable approach for the generation of α-carbon radicals of amines and ethers.^3^ This approach facilitates coupling reactions of the generated radicals with aromatic heterocycles under mild conditions. One of the most fascinating aspects of this visible light-based photochemistry is that the reaction can be conducted with a small quantity of a metal-based photocatalyst and a household compact fluorescent light (CFL) bulb, which is easier to handle than a UV lamp. Further, complex organic molecules are more stable towards photodecomposition when they are irradiated with lower energy wavelengths.^4^ Nevertheless, the current methodologies require pre-functionalized heteroaromatics (with CN and Cl) that consequently produce stoichiometric amounts of undesired waste (Fig. 1a), and the coupling partners are limited to amines and ethers.^3^ The reactions of electron-poor amide substrates remain unexplored. Thus, the development of new photoredox cross-dehydrogenative-coupling (CDC) reactions, which can couple medicinally important heterocycles with a broader scope of coupling partners and selectively form C–C bonds between two different inert C–H bonds without any pre-functionalization, is highly desirable.^5^

Notably, MacMillan and co-workers recently reported a CDC-based reaction for electron-deficient heteroarenes through a combination of iridium- or ruthenium-containing photoredox catalysis with a Minisci-type reaction of α-oxy radicals (Fig. 1b).6*a* A similar strategy was also applied in the synthesis of a JAK2 inhibitor by Stephenson *et al.*6*b* However, these reported reactions only coupled six-membered nitrogen-containing heteroaromatics with ethers or electron-rich amines. There is an urgent need to develop an efficient photochemically induced CDC reaction for a broader range of heteroaromatics with diverse amines, amides, and ethers. It would also be advantageous that the new reaction avoids the use of expensive transition metal catalysts or toxic/explosive reagents. Herein, we report a photoredox CDC reaction that enables the selective α-heteroarylation of amides (α to nitrogen) and ethers through C–H activation (Fig. 1c). This protocol features a distinct decomposition mechanism of APS promoted by photoexcited benzaldehydes, metal-free reaction conditions using an environment-friendly oxidant radical initiator and 23 W CFL bulbs, and the coupling of amides and ethers with five- and six-membered electron deficient heteroarenes.

## Results and discussion

(NH_4_)_2_S_2_O_8_ (ammonium persulfate, APS) is widely used as a cheap and environmentally benign radical initiator in organic synthesis and the polymer industry.^7^ In combination with a metal-based photocatalyst, such as Ru(bpy)_3_Cl_2_, visible light can drive the decomposition of APS to produce sulfate radicals, which are widely involved in the initiation of diverse radicals through a hydrogen atom transfer (HAT) process.^6^,^8^ This strategy has been successfully implemented in the CDC reactions of ethers with heteroaromatics.^6^ We questioned whether an α-amino radical could also be initiated *via* this photochemically induced process followed by a Minisci-type reaction with electron-deficient heteroarenes to create a new CDC reaction.

Formamide has a relatively low C–H bond strength (BDE = 94 kcal mol^–1^)^9^ that tends to promote homolytic cleavage to generate a C_sp^2^_-based nucleophilic α-amino radical. Moreover, a sunlight-induced coupling reaction of formamide with benzene-fused pyridines was successfully implemented with polycrystalline TiO_2_ as the catalyst. This reaction exhibits a low reaction efficiency and limited substrate scope.^10^ Our examination of the proposed photochemically induced CDC reaction with heteroaromatics began with formamide and benzothiazole given the utility of benzothiazoles in biomedicine.^11^ The initial reaction employing 2 mol% of Ru(bpy)_3_Cl_2_ provided a carbamoylated benzothiazole, however, with a low yield (entry 1 in Table 1, 6% yield). Surprisingly, the product was obtained in a moderate yield when the photocatalyst was excluded (entry 2, 59% yield). Lowering the amount of APS resulted in a decreased yield (entry 5, 25% yield). The careful exclusion of light had little effect on the reaction (entry 4, 55% yield), while decreasing the temperature led to lower reaction efficiency (entry 3, 15% yield). This result may be attributed to the temperature-dependent decomposition of APS enhanced by formamide, which has been described in the APS-initiated polymerization of acrylamide.^12^

**Table 1:** Preliminary studies towards the carbamoylation of benzothiazole^*a*^

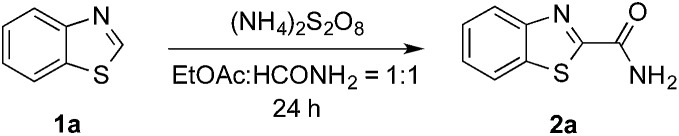				
Entry	(NH_4_)_2_S_2_O_8_	Photosensitizer	TsOH	Yield^*b*^
1^*c*^	3 equiv.	—	—	6%
2	3 equiv.	—	—	59%
3^*d*^ ^,^^*e*^	3 equiv.	—	—	15%
4^*e*^	3 equiv.	—	—	55%
5	1 equiv.	—	—	25%
6	3 equiv.	**P1** (0.5 equiv.)	—	90%
7	3 equiv.	**P1** (1.0 equiv.)	—	95%
8^*d*^ ^,^^*e*^	3 equiv.	**P1** (1.0 equiv.)	—	3%
9	—	**P1** (1.0 equiv.)	—	n.r.^*f*^
10	3 equiv.	**P2** (1.0 equiv.)	—	84%
11	3 equiv.	**P3** (1.0 equiv.)	—	34%
12^*g*^	3 equiv.	**P1** (1.0 equiv.)	—	46%
13^*h*^	3 equiv.	**P1** (1.0 equiv.)	—	13%
14^*i*^	3 equiv.	**P1** (1.0 equiv.)	—	n.r.^*f*^
15	3 equiv.	**P1** (1.0 equiv.)	1.0 equiv.	99% (94%^*j*^)
16	1 equiv.	**P1** (1.0 equiv.)	1.0 equiv.	70%
17	3 equiv.	—	1.0 equiv	86%
18^*k*^	3 equiv.	**P1** (1.0 equiv.)	1.0 equiv.	85%^*j*^

^*a*^See general procedures A–C for the experimental details unless otherwise noted.

^*b*^Yields were determined using ^1^H NMR spectroscopy with CH_2_Br_2_ as an internal standard.

^*c*^Performed with Ru(bpy)_3_Cl_2_ (2 mol%).

^*d*^Performed at 23 °C.

^*e*^Performed in darkness.

^*f*^No reaction.

^*g*^Performed with HCONH_2_ (50 equiv.).

^*h*^Performed under air.

^*i*^Performed with TEMPO (3 equiv.).

^*j*^Isolated yield.

^*k*^Performed on a 1 g scale.

Interestingly, even 0.5 equivalents of benzaldehyde can promote the reaction (entry 6 in Table 1, 90% yield). In the absence of CFL illumination, benzaldehyde did not improve the yield (entry 8, 3% yield). In addition, no product was detected when the reaction was performed with benzaldehyde but not APS (entry 9). These results indicated that photoexcited benzaldehyde might be involved in this photoredox process to promote the decomposition of APS and the generation of the carbamoyl radical. Benzophenone, which is reported to mediate a SET process upon illumination with CFL bulbs, can also improve the yield (entry 10, 84% yield). On the other hand, 9-fluorenone that displays a stronger absorption in the visible light region (Fig. S2†) completely suppressed the enhanced reactivity (entry 11, 34% yield, *versus* entries 2 and 7 in Table 1).^13^ Therefore, 1.0 equivalent of benzaldehyde was selected for further optimization. Lowering the amount of formamide to 50 equivalents resulted in a decreased yield (entry 12, 46% yield). The reaction was inhibited in the presence of air (O_2_) and TEMPO (entries 13 and 14), again suggesting a radical mechanism. Further, *p*-toluenesulfonic acid monohydrate (1 equiv.) can improve the yield (entry 15, 99% yield) through the protonation and activation of benzothiazole. In the presence of *p*-toluenesulfonic acid, lowering the amount of APS (entry 16) or the exclusion of benzaldehyde (entry 17) led to decreased yields. Hence, the optimized reaction conditions are shown in entry 15. With these conditions in hand, the reaction was successfully implemented on a gram scale (entry 18, 85% isolated yield).

The synthetic potential of this new photoredox reaction was then evaluated with different heteroarene compounds. For benzothiazole substrates with electron-withdrawing or weak electron-donating groups, the reactions proceeded well (**2b–e** in Table 2, 50–85% yields) with excellent selectivity at the C2 position. Bromide, ester, and acyl groups were well tolerated, providing handles for functionalization. However, the introduction of a stronger electron-donating group, such as an acetamido group, to benzothiazole resulted in diminished reactivity towards the nucleophilic addition of the carbamoyl radical, lowering the yield (**2f**, 30% yield). These results further indicated the nucleophilic nature of the reaction. Benzimidazoles also functioned as suitable substrates (**2g–k**, 64–85% yields). Benzoxazole was not as well tolerated for this protocol (**2l**, trace) with the reaction resulting in a complicated mixture perhaps owing to its instability in the presence of strong oxidants.^14^ Finally, a monocyclic thiazole was also amenable to this coupling strategy (**2m**, 54% yield).

**Table 2:** Benzaldehyde-mediated photoredox CDC reactions of azoles and benzazoles with formamide^*a*^

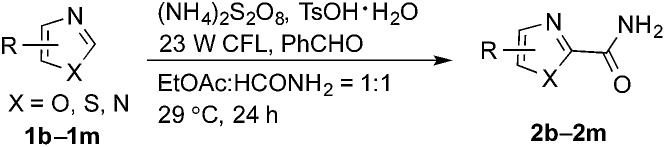
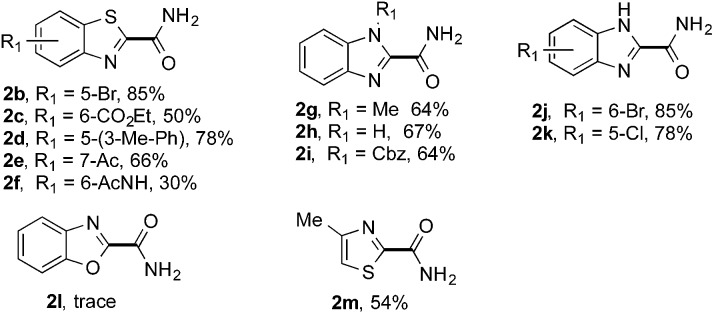

^*a*^See general procedure A for the experimental details unless otherwise noted. The isolated yield is reported for each reaction.

Having successfully demonstrated the carbamoylation of a variety of azole heterocycles, we anticipate that electron-deficient six-membered nitrogen-containing heteroaromatics can also be engaged with this new protocol (Table 3). Pyridine substrates underwent coupling with moderate yields (**4a–c** in Table 3a, 60–67% yields). Pyridine or pyrazine fused with a benzene ring displayed increased reaction efficiency and produced excellent yields even without an acidic additive (**4d–g**, 71–95% yields). For pyridine-containing substrates, *ortho* substitution is more favored than *para* substitution (**4c**, C-6 : C-2 : C-4 = 16.5 : 5.5 : 1; and **4e**, C-2 : C-4 = 3 : 1). The bis-addition products proved to be minimal. The H-bond between the pyridyl nitrogen atoms of the substrates and the formamide amide group is expected to account for this regioselectivity. Not surprisingly, the C-4 reactivity of 2-chloroquinoline was dramatically diminished and resulted in a low yield (**4h**, 15% yield). Interestingly, the reaction with 4-chloroquinazoline (it contains two electron-withdrawing groups: the pyridine nitrogen atom and the chlorine atom) led to an excellent yield (99% NMR yield after 6 h) even with 0.1 equivalents of benzaldehyde, as shown in Table 3b. In contrast, a yield of 13% was achieved in the absence of the aldehyde. These results suggested that benzaldehyde might function through a catalytic cycle in this reaction. Carboline, a widely used chemical scaffold in drug discovery,^15^ was also amenable to this protocol, albeit with a moderate yield (**4i** in Table 3a, 50% yield). It is worth noting that this protocol can also be implemented in the synthesis of **4a**, a key intermediate for the preparation of an NMDA antagonist, CGS 19755 (**4a**, 67% yield. The literature yield was 30–40% with the reaction conditions of H_2_SO_4_, 30% H_2_O_2_, and FeSO_4_·7H_2_O).2*a*

**Table 3:** Benzaldehyde-mediated photoredox CDC reactions of six-membered nitrogen-containing heteroaromatics with formamide^*a*^

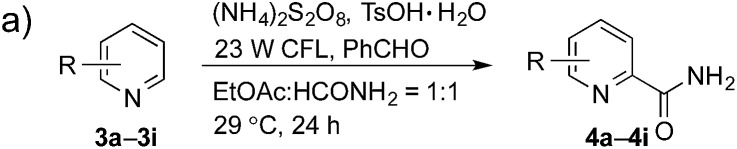
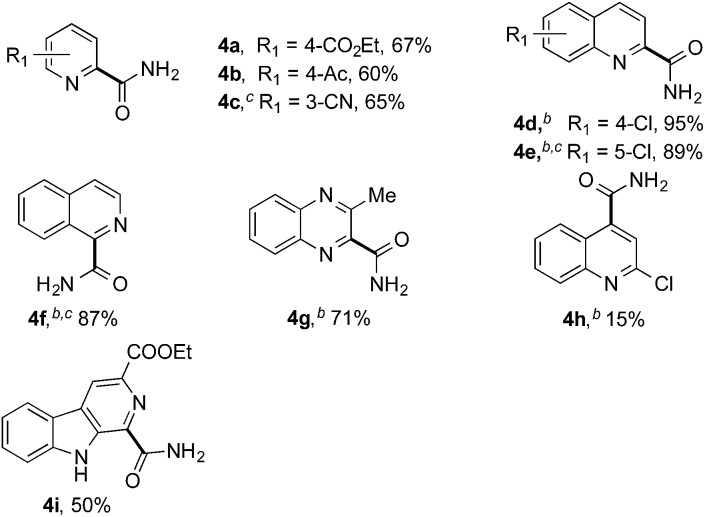

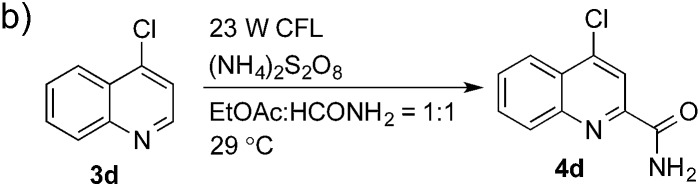			
Entry	Additive	Time	Yield
1	—	6 h	13%
2	PhCHO (1 equiv.)	6 h	>99%
3	PhCHO (0.1 equiv.)	6 h	>99%

^*a*^See general procedure A for the experimental details unless otherwise noted. The isolated yield is reported for each reaction.

^*b*^Performed without TsOH·H_2_O (general procedure B).

^*c*^The reaction to generate **4c**, C-6 : C-2 : C-4 = 16.5 : 5.5 : 1 (r.r.); the reaction to generate **4e**, C-2 : C-4 = 3 : 1 (r.r); and the reaction to generate **4f**, C-1 product only. The regiomeric ratio (r.r.) was determined using ^1^H NMR spectroscopy.

Reactions between benzothiazole and *N*-alkylated amides were also investigated (Table 4). *N*-Methylformamide underwent an arylation with approximately 8 : 1 regioselectivity to activate the carbonyl C_sp^2^_–H bond (**5a**, 88% yield). *N*,*N*-Dimethylformamide (DMF) mainly led to the activation of the C_sp^3^_–H bond of the *N*-methyl group, affording the opposite regioselectivity (**6a**, 80% yield). The different BDEs of the carbonyl C_sp^2^_–H and *N*-methyl C_sp^3^_–H bonds of DMF account for this selectivity.^16^*N*-Methylacetamide and *N*,*N*-dimethylacetamide (DMA) do not have a C_sp^2^_–H bond and only undergo arylation at the *N*-methyl group through the activation of the C_sp^3^_–H bond (**6b** and **6c**). The reaction with *N*-methyl pyrrolidin-2-one also displayed excellent efficiency (**6d**, 89% yield). The regioselectivity (r.r. = 12 : 1) favors the methylene carbon over the *N*-methyl carbon. It should be noted that all of these reactions were conducted in the absence of TsOH·H_2_O. The reaction with *N*,*N*-diethylacetamide activated the C_sp^3^_–H bond next to the nitrogen atom. However, the yield was low even with the use of one equivalent of TsOH·H_2_O (**6e**, 25% yield).

**Table 4:** Benzaldehyde-mediated photoredox CDC reactions between benzothiazole and different amides^*a*^

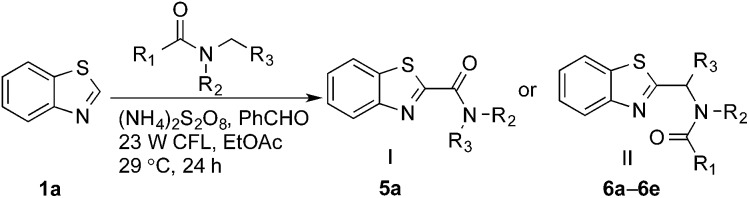			
Entry	Substrate	Product	Yield
1	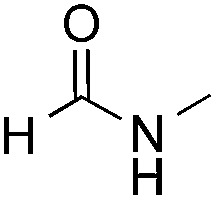	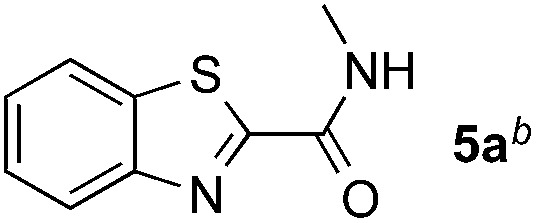	88%
2	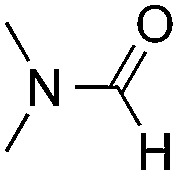	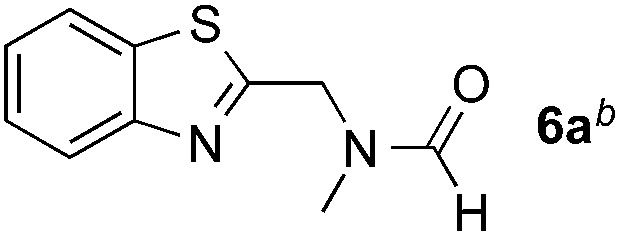	80%
3	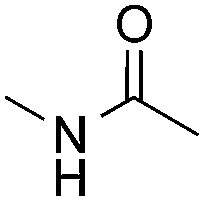	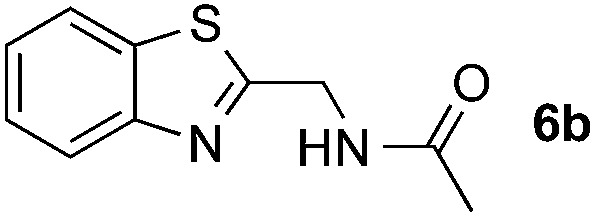	82%
4	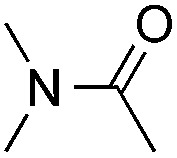	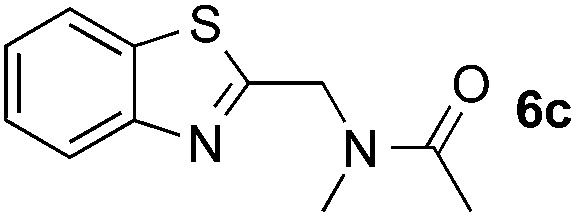	87%
5	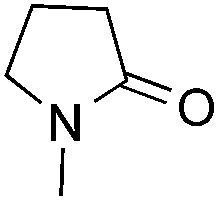	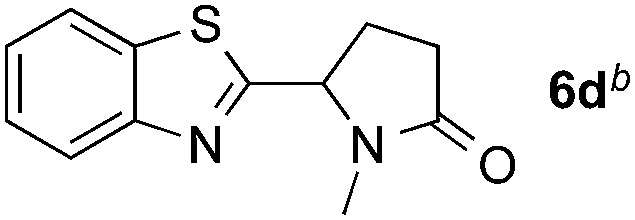	89%
6	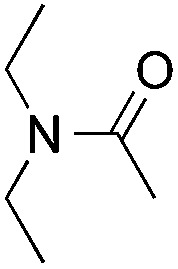	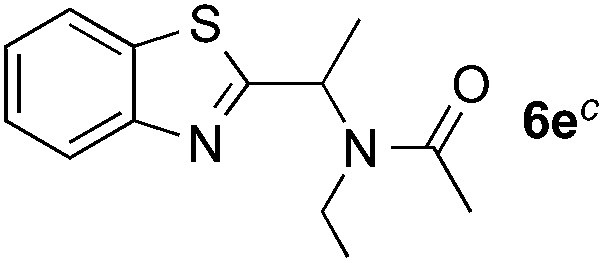	25%

^*a*^See general procedure C for the experimental details. All reactions are reported with the isolated yields unless otherwise noted.

^*b*^Entry 1, 8 : 1 (r.r.) = structure I : structure II; entry 2, 1 : 9 (r.r.) = structure I : structure II; and entry 5, 12 : 1 (r.r.) = 1-methyl-5-substituted pyrrolidin-2-one : 1-substituted methyl pyrrolidin-2-one. The regiomeric ratio (r.r.) was determined using ^1^H NMR spectroscopy.

^*c*^Performed with TsOH·H_2_O (1 equiv.).

### Mechanism

**Fig. 2:**
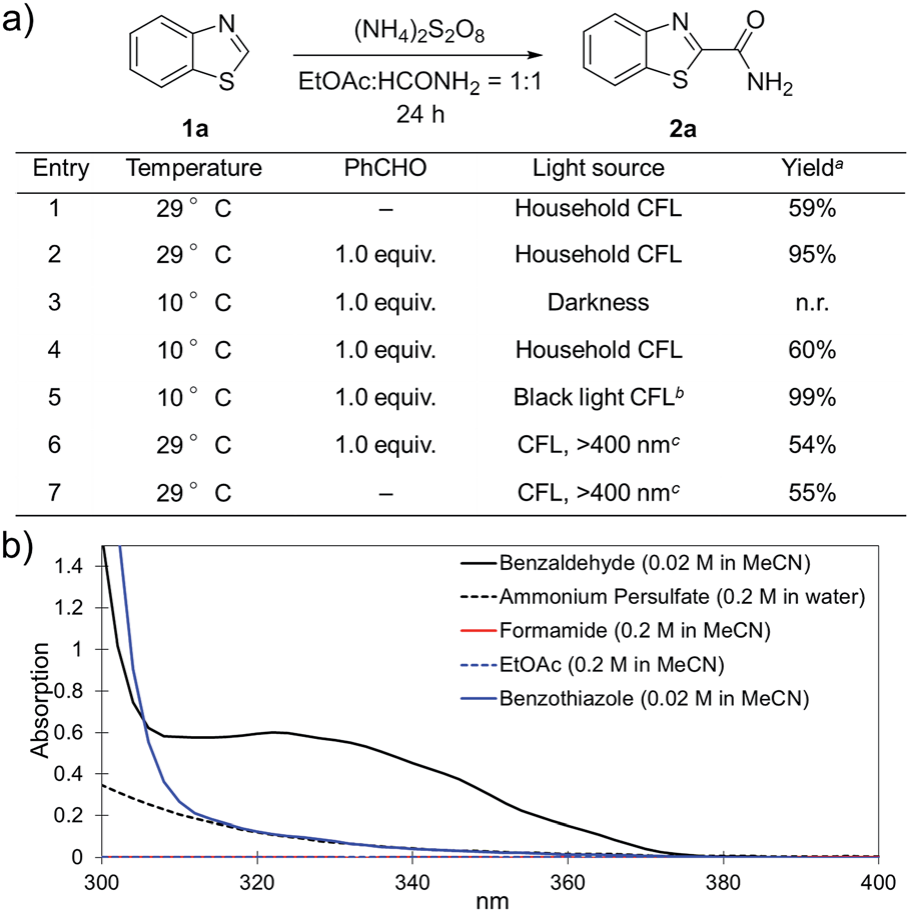
(a) Investigation of the role of light in the reaction. ^a^ Yields were determined using ^1^H NMR spectroscopy with CH_2_Br_2_ as an internal standard; ^b^ UV-A light; ^c^ water bath with 1 M NaNO_2_ aqueous solution to suppress light with *λ* ≤ 400 nm; and n.r., no reaction. (b) UV-Vis absorption spectra of the reagents.

To further disclose the photochemical nature of the reaction, we performed several control experiments by varying the wavelengths of the light (Fig. 2a). The initial results indicated that the near UV region of the CFL emission spectrum (364 nm) contributed to this conversion (entry 3, no reaction in darkness; entry 4, 60% yield, irradiated with a household CFL bulb; and entry 5, 99% yield, irradiated with a black light CFL bulb, UV-A light). A similar yield was achieved for entries 6 and 7, indicating that the benzaldehyde-enhanced reactivity was completely suppressed when the reaction was performed under filtered light (>400 nm). The UV-visible absorption spectra of the reagents were collected as shown in Fig. 2b. No obvious absorption was detected for formamide, ethyl acetate, APS and benzothiazole in the near UV region of the CFL emission spectrum (*e.g. λ* = 364 nm), while benzaldehyde displayed a strong concentration-dependent absorption (Fig. S3†). We then investigated the correlation of the photochemical activity of the reaction with benzaldehyde. No ground state association was detected between the reaction components, as shown in Fig. S4.† These experimental results again suggested that benzaldehyde works *via* an excited state through an n–π* transition.17*a* However, it is not clear if the triplet state, which plays a dominant role in the photochemistry of benzaldehyde and has a relatively long lifetime, is involved in this process, because the triplet state of benzaldehyde tends to be quenched by acid.17a,^18^

The coupling reactions of dioxane with benzothiazole were then performed to further investigate the mechanism for the generation of the sulphate radical. Further, we envisioned that this CDC reaction might be used to install an ether functionality on heteroaromatics due to the stability of C_α_ radicals towards oxygen atoms. As shown in Table 5, in the absence of acetamide (please note that acetamide does not have a C–H bond α to the nitrogen atom) and benzaldehyde, no product was observed in the reaction with 1,4-dioxane. A yield of 32% was achieved with the use of 20 equivalents of acetamide. This reaction again suggested that the amide-enhanced decomposition of APS at low temperature accounted for the reactivity due to hydrogen bonding and donor–acceptor interactions between acetamide and APS.^12^ The addition of one equivalent of benzaldehyde to this acetamide-containing reaction mixture improved the yield to 68%. A similar yield was achieved in the absence of acetamide (entry 4 in Table 5, 70% yield). The isolated yield of this reaction was 62%. These results indicated that photoexcited benzaldehyde worked *via* the direct promotion of APS decomposition and contributed to the initiation of the radical chain reaction. The same result was also obtained for glycol dimethyl ether with an isolated yield of 64% (entry 5 in Table 5).

**Table 5:** Investigation of the CDC reactions of ethers with benzothiazole^*a*^

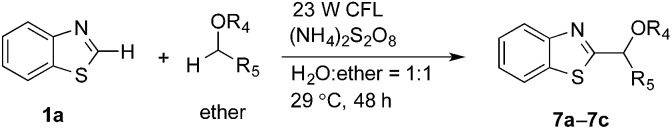					
Entry	Ether	CH_3_CONH_2_	PhCHO	Product	Yield^*b*^
1	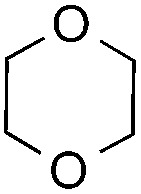	—	—	—	n.r
2	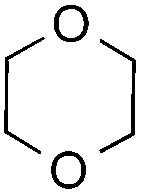	20 equiv.	—	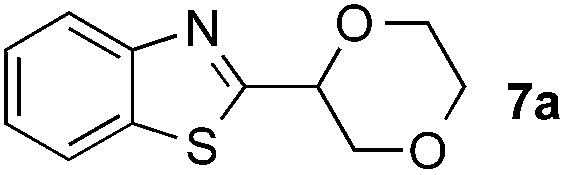	32%
3	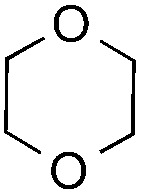	20 equiv.	1 equiv.	**7a**	68%
4	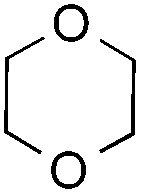	—	1 equiv.	**7a**	70% (62%^*c*^)
5	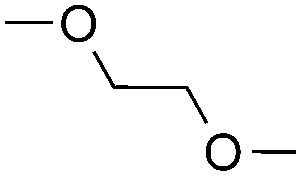	—	1 equiv.	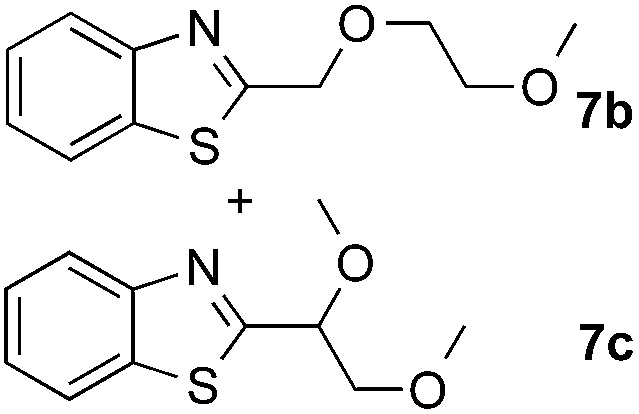	(64%^*c*^) r.r. = 1 : 1

^*a*^See general procedure D for the experimental details.

^*b*^Yields were determined using ^1^H NMR spectroscopy with CH_2_Br_2_ as an internal standard; n.r., no reaction; r.r. = regioselectivity ratio between **7b** and **7c**.

^*c*^Isolated yield.

**Fig. 3:**
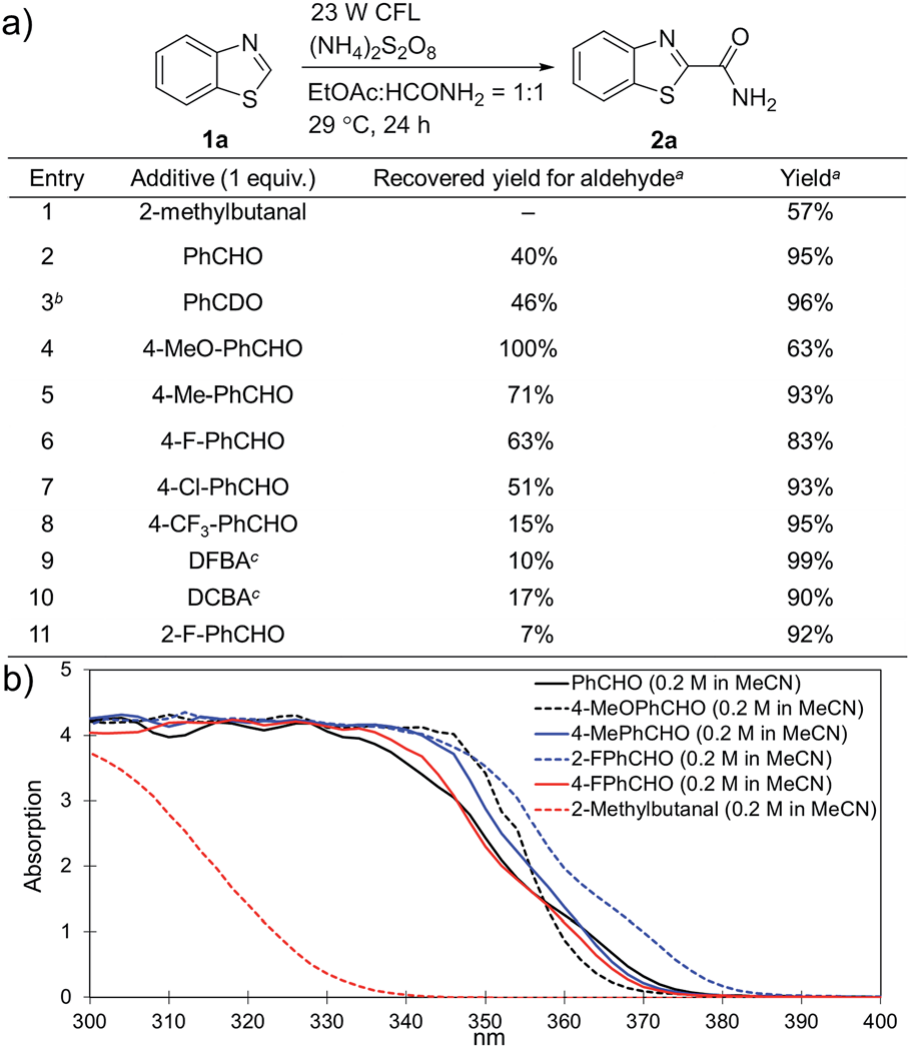
Investigation of the role of benzaldehyde in the reaction. (a) Evaluation of different aldehydes. ^a^ Yields were determined using ^1^H NMR spectroscopy with CH_2_Br_2_ as an internal standard; ^b^ PhCDO was recovered without deuterium exchange; ^c^ DFBA stands for 2,5-difluorobenzaldehyde, and DCBA stands for 2,5-dichrolobenzaldehyde. (b) UV-Vis absorption spectra for different benzaldehydes.

Substituted benzaldehydes were then used to further investigate the mechanism. 2-Methylbutanal without an aromatic system cannot improve the yield of the reaction (entry 1 in Fig. 3a, 57% yield), which again suggests that benzaldehyde functions through absorbing near UV light from the CFL bulb. No hydrogen/deuterium exchange was observed for the reaction with benzaldehyde-α-d_1_ (entry 3, 96% yield), suggesting that the benzoyl radical was not involved in this reaction. No yield improvement was observed for a benzaldehyde with strong electron-donating groups, such as 4-methoxybenzaldehyde (entry 4, 63% yield), which was previously reported to promote the atom-transfer radical addition of alkenes as an excellent energy transfer catalyst.17*a* This result is indicative that benzaldehyde might not work *via* an energy-transfer pathway in the studied reactions. It should be noted that 4-methoxybenzaldehyde was fully recovered in this reaction. On the other hand, the benzaldehydes with electron-withdrawing groups and weak electron-donating groups promoted the reactions accompanied with partial decomposition (entries 2 and 5–11, 83–99% yields with 7–71% recovered yields for the benzaldehydes). The results suggest that a study of the decomposition of benzaldehyde may help disclose the reaction mechanism. The UV-Vis absorption spectra of different benzaldehydes were collected. As shown in Fig. 3b, the substituted benzaldehydes, which displayed stronger absorption in the near UV region of the CFL emission spectrum (*e.g. λ* = 364 nm), suffered more decomposition.

**Scheme 1:**
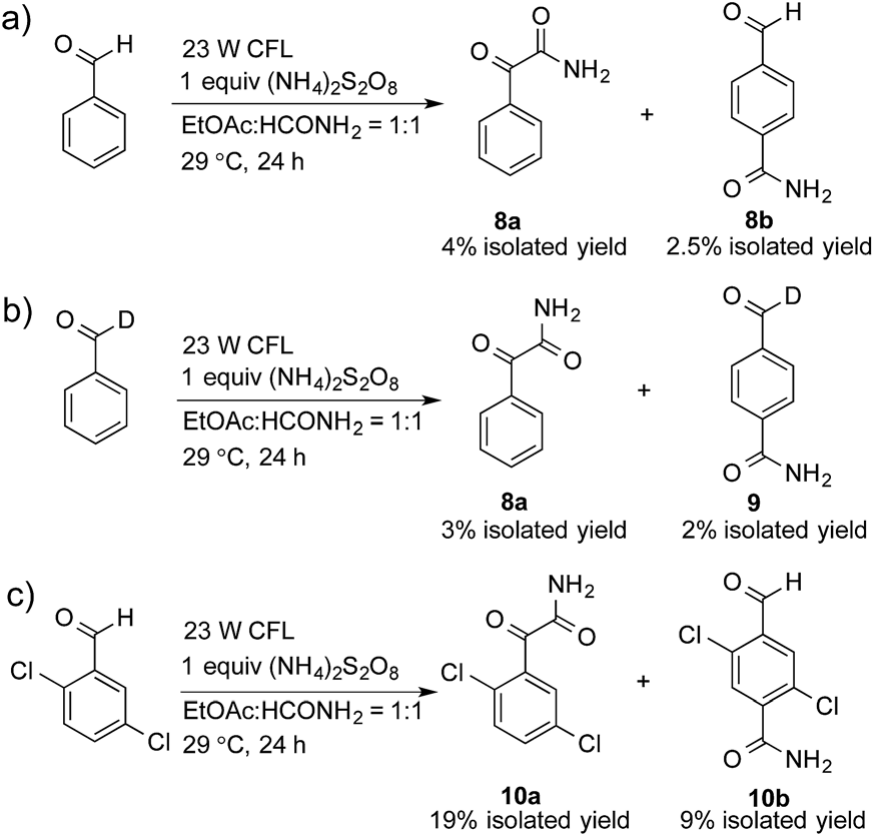
Reactions to study the products of benzaldehyde-mediated photoredox CDC reactions.

A control experiment without the addition of heteroaromatics, such as benzothiazole, was conducted to study the decomposition of benzaldehyde. Only 2-oxo-2-phenylacetamide (**8a**, 4% isolated yield) and 4-formylbenzamide (**8b**, 2.5% isolated yield) were detected, as shown in Scheme 1a. A possible mechanism for the decomposition of benzaldehyde in this reaction is shown in Scheme S1.† Photoexcited benzaldehydes **V** and **VI** that are generated upon illumination might be the key intermediates. The coupling reaction between the photoexcited benzaldehydes and the carbamoyl radical, followed by the extraction of hydrogen, could afford **8a** and **8b**. Two other control reactions with benzaldehyde-α-d_1_ and 2,5-dichlorobenzaldehyde were also conducted to study the decomposition of the benzaldehydes. Similar results were obtained, as shown in Scheme 1b and c.

**Scheme 2:**
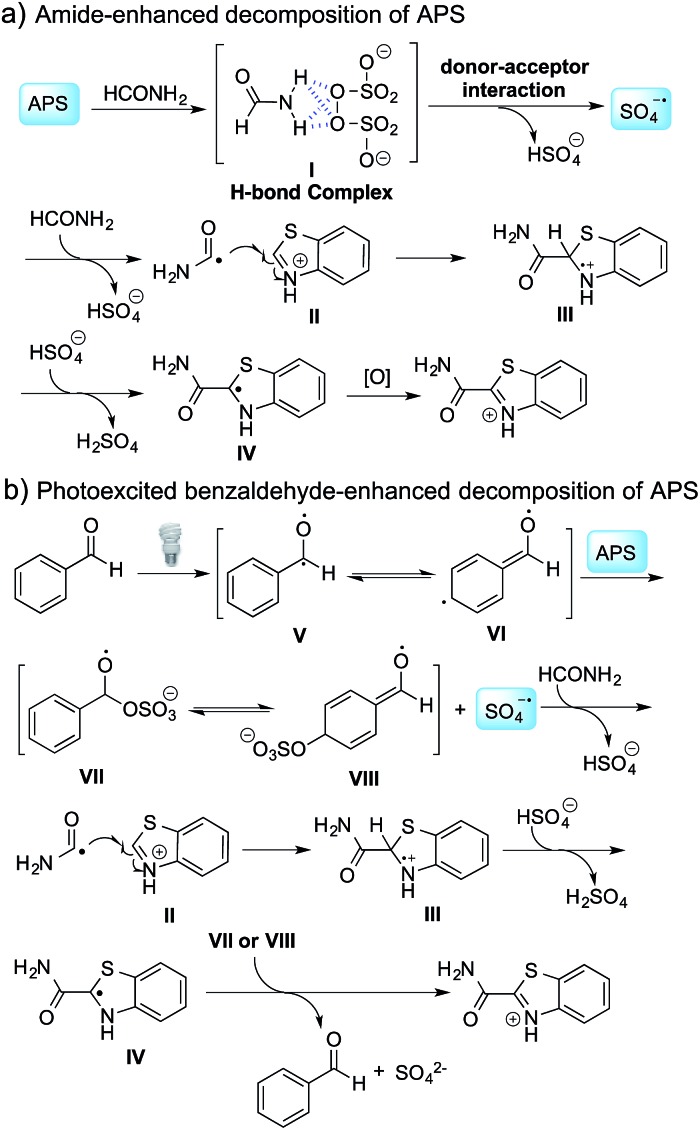
Proposed reaction mechanism.

The proposed mechanism is shown in Scheme 2. In the absence of benzaldehyde, hydrogen bonding between APS and formamide leads to the formation of complex **I**,^12^ followed by the generation of a sulfate radical through a donor–acceptor interaction and the decomposition of this complex. When benzaldehyde is added to the reaction mixture, photoexcited benzaldehydes **V** and **VI** are generated upon illumination^13^ and promote the decomposition of APS to generate a sulfate radical. The carbamoyl radical from the reaction between the sulfate radical and formamide undergoes a nucleophilic addition with the protonated benzothiazole at the C-2 position, followed by deprotonation and oxidation to provide the desired product. It should be noted that intermediates **VII** or **VIII** might be involved in the oxidation step, and thereby regenerated benzaldehyde to finish the catalytic cycle based on the observation that a catalytic amount of benzaldehyde can also promote the reaction (Table 3b). Further validation of the mechanism is still ongoing in our lab.

### Application

**Scheme 3:**
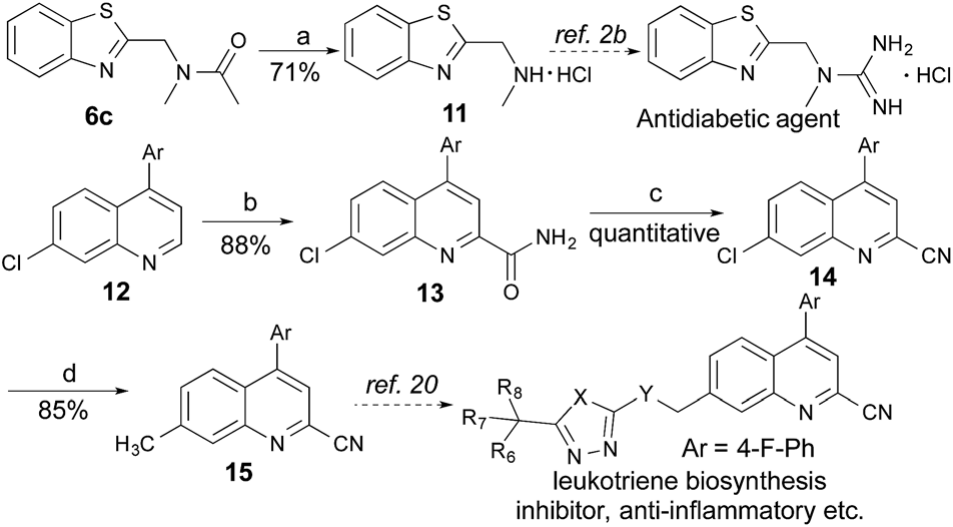
New CDC strategy to synthesize two key intermediates for biologically active compounds. Regents and conditions: (a) HCl (6 M), EtOH, reflux, overnight; (b) 23 W CFL, (NH_4_)_2_S_2_O_8_, PhCHO, HCONH_2_, EtOAc; 29 °C, 24 h; (c) TFAA, TEA, THF, 0 °C, 2 h; (d) Pd(OAc)_2_, MeB(OH)_2_, SPhos, K_3_PO_4_, toluene, reflux, 36 h.

Many therapeutic drugs feature an amine or ether C_α_ to a heteroarene ring (Fig. S1†). The new CDC protocol described in this study is readily applicable to the efficient assembly of compounds in this class (Schemes 3, S2, and S3†). For example, starting with **6c**, the hydrolysis reaction led to key intermediate **11** for the synthesis of an antidiabetic agent.2*b* The new reaction was also successfully applied to the installation of a cyano group at the C-2 position of **12** with an excellent yield (88% yield over two steps) through a coupling reaction with formamide followed by a dehydration reaction under moderate reaction conditions, thereby facilitating the synthesis of a leukotriene biosynthesis inhibitor.^19^ Given the prevalence of nitrile-containing pharmaceuticals^20^ and the difficulty in performing late-stage functionalization of the nitrile group (previous reactions typically needed metal catalysts, high temperatures, toxic reagents, *etc.*),^21^ this new strategy will find broad application in drug discovery programs. In combination with the Suzuki–Miyaura coupling reaction, a new synthetic route was successfully implemented to generate key intermediate **15** with 75% overall yield.

## Conclusions

In this study, we developed an effective photochemically induced CDC strategy for the α-heteroarylation of amides (α to nitrogen) and ethers. A new decomposition mechanism of APS enhanced by amides and photoexcited benzaldehydes was disclosed. Remarkably, both C_sp^3^_–H activation (the *N*-alkyl C–H bonds of the amide or the C_α_–H bonds of the ether) and C_sp^2^_–H activation (the carbonyl C–H bond of formamide) were amenable to this protocol. The reaction is environmentally benign and metal-free, is conducted at room temperature with readily available reagents, and uses household CFL bulbs as the light source. These features will likely render this reaction a useful tool for the synthesis of biologically active compounds and the design of new radical-based reactions. However, a large excess of amides and ethers is required for the effective conversion of this reaction. Future research aims to optimize the reaction to decrease the required amount of the amide or ether coupling partners and to study the application of this new strategy for the synthesis of new biologically active compounds.

## Supplementary Material

Supplementary information
