# Supplementary material for: Direct α-heteroarylation of amides (α to nitrogen) and ethers through a benzaldehyde-mediated photoredox reaction
Source: Chem Sci. 2015 Dec 7;7(3):2111–8. doi: 10.1039/c5sc03640b (PMC5968545; doi:10.1039/c5sc03640b)

Electronic Supplementary Material (ESI) for Chemical Science.
This journal is © The Royal Society of Chemistry 2015

Direct α-Heteroarylation of Amides (α to Nitrogen) and Ethers through a Benzaldehyde-Mediated Photoredox Reaction

Yongqiang Zhang, Kevin B. Teuscher, Haitao Ji*

Department of Chemistry, Center for Cell and Genome Science, University of Utah, Salt Lake City, Utah 84112–0850, USA
*Correspondence to: Professor Haitao Ji (E-mail: markji@chem.utah.edu)

Table of Contents

Figure S1……………………………………………………………………………… S2
Figure S2………………………………………………………………………………S2
Figure S3………………………………………………………………………………S3
Figure S4………………………………………………………………………………S3
Scheme S1…………………………………………………………………………… S4
Scheme S2……………………………………………………………………………S5
Scheme S3……………………………………………………………………………S5
Experiment Procedures and Product Characterization……………………………………S6–S21
Supplementary References…………………………………………………………………S22
Spectral Data for the Products……………………………………………………………S23-S58

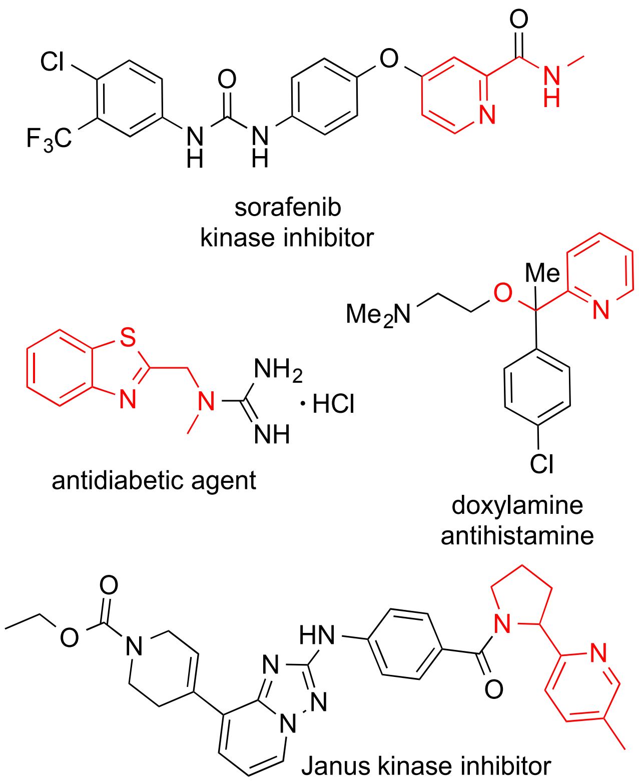

Figure S1. Bioactive compounds that contain a heteroaryl *α*-amine/ether functionality

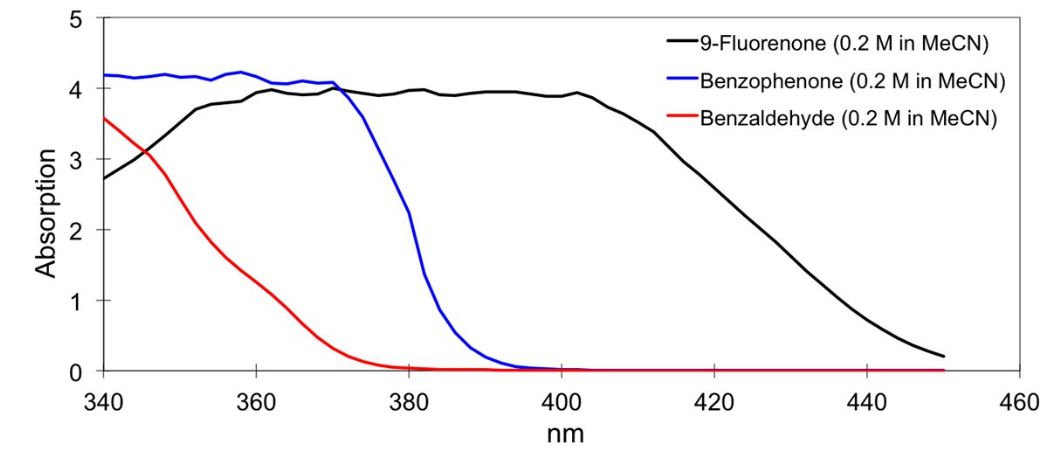

Figure S2. UV-Vis absorption spectra of photosensitizers

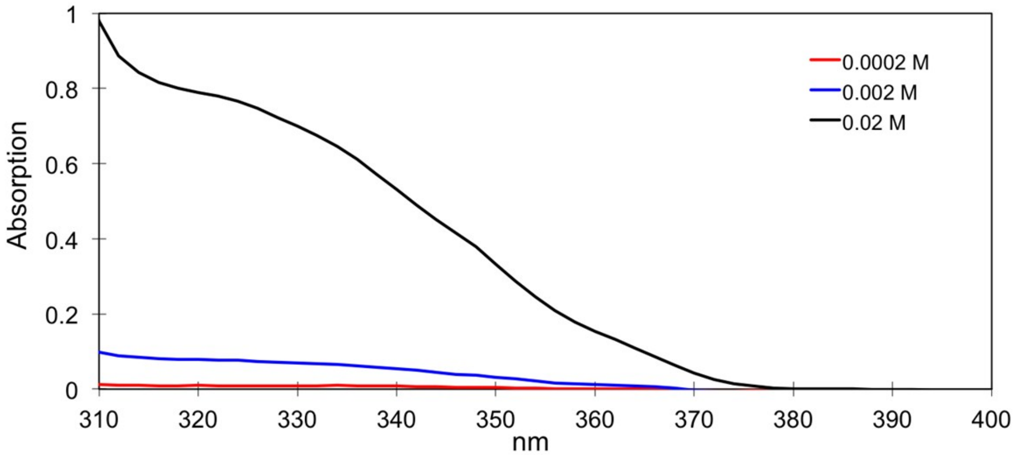

Figure S3. UV-Vis absorption spectra of different concentrations of benzaldehyde in the reaction solvent, EtOAc:$HCONH_2$ = 1:1.

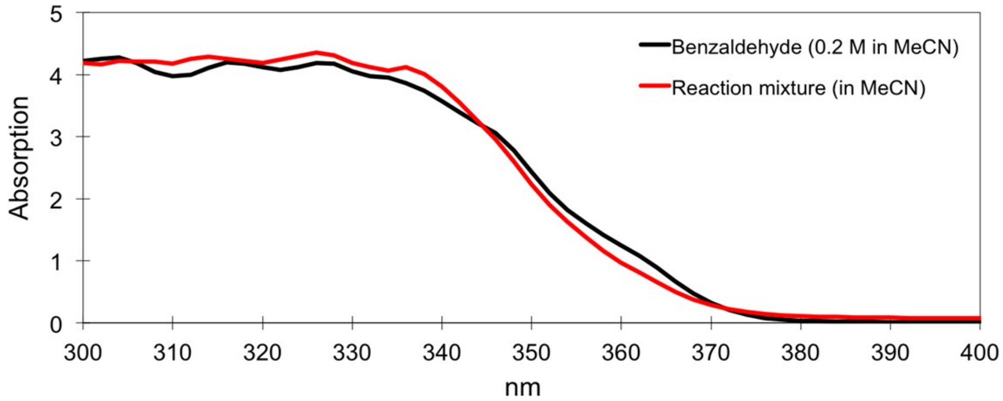

Figure S4. UV-Vis absorption spectra of benzaldehyde and the reaction mixture in acetonitrile.

Scheme S1. Plausible Mechanism for the Decomposition of Benzaldehyde

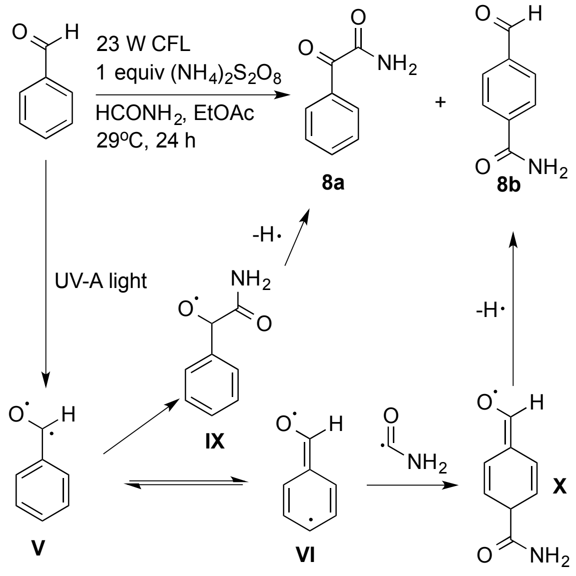

Scheme S2. Comparison of Two Routes towards the Synthesis of a Key Intermediate for an Antidiabetic Agent

Reported synthetic route (*ref.1*):

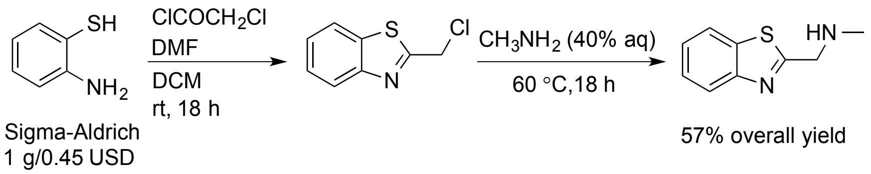

Our new method:

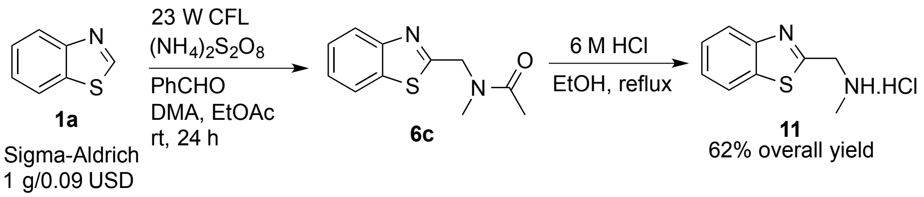

Scheme S3. Comparison of Two Methods to Install a Cyan Group at Quinoline in the Synthesis of a Leukotriene Biosynthesis Inhibitor

Reported synthetic route (*ref.2*):

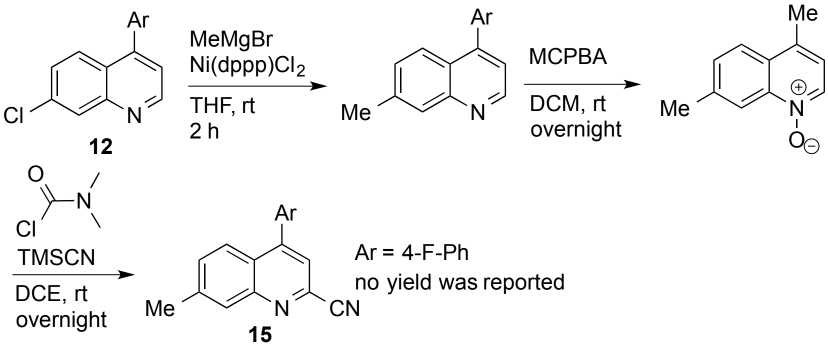

Our new method:

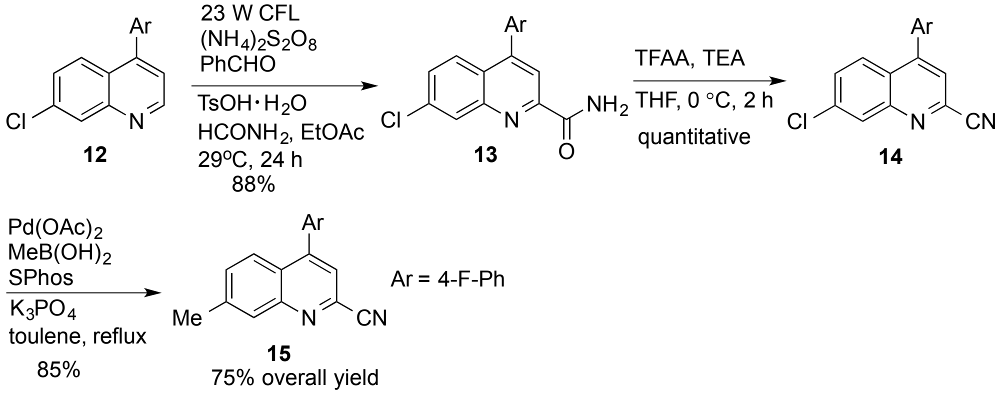

Experiment Procedures and Product Characterization

Commercial reagents and solvents were used as received, unless otherwise stated. Organic solution was concentrated under reduced pressure on a Büchi rotary evaporator using an isopropyl alcohol-dry ice bath. Analytical thin layer chromatography (TLC) was performed on 0.25 mm silica gel plates (SiliCycle Inc., Canada), and the compounds were visualized with a UV light at 254 nm. Further visualization was achieved by staining with iodine. Flash chromatography was performed on silica gel 230–400 mesh (SiliCycle Inc., Canada) with commercial solvents. The ^{1}H and ^{13}C NMR spectra were recorded on a Varian Unity 300 Spectrometer (300 and 75 MHz for ^{1}H and ^{13}C NMR, respectively) or a VXR 500 Spectrometer (500 and 125 MHz for ^{1}H and ^{13}C NMR, respectively) and are internally referenced to residual solvent signals (note: d^6-DMSO referenced at 2.50 and 39.52 ppm in ^{1}H and ^{13}C NMR, respectively). Multiplicities were given as s (singlet), d (doublet), t (triplet), dd (double of doublet), and m (multiplets). Coupling constants were reported in Hertz (Hz). Data for ^{13}C NMR are reported in terms of chemical shift. High-resolution mass spectrometry (HRMS) was recorded on Waters LCT Premier XE TOF with Acquity Classic UPLC ToF High Resolution Exact Mass.

General procedure for the carbamoylation of heteroaromatics.

Method A (with TsOH•H_2O): To a 10 mL (or 20 mL) schlenk tube equipped with a magnetic stir bar was charged a heteroarene (0.22 mmol, 1.0 equiv), $(NH_4)_2S_2O_8$ (0.15 g, 0.66 mmol, 3.0 equiv), TsOH•H_2O (0.042 g, 0.22 mmol, 1.0 equiv), benzaldehyde (0.023 g, 0.22 mmol, 1.0 equiv), 1.5 mL ethyl acetate, and 1.5 mL formamide. The reaction mixture was degassed via freeze pump thaw (× 3 times) and refilled with argon. After the reaction mixture was thoroughly degassed, the vial was sealed and positioned approximately 10 cm away from the light source. Two household full-spectrum 23 W CFL bulbs were used to irradiate the reaction mixture. Water bath was used to keep the reaction temperature at 29 °C. After stirring for the indicated time, the resulting mixture was diluted with water (20 mL). The pH value was then adjusted to 9–10 with NaOH (1 M), extracted with ethyl acetate (15 mL × 3), dried over Na_2SO_4, and concentrated under vacuum. Purification of the crude product by flash chromatography on silica gel using the indicated solvent system afforded the desired product.

Method B (without TsOH•H_2O): To a 10 mL (or 20 mL) schlenk tube equipped with a magnetic stir bar was charged a heteroarene (0.22 mmol, 1.0 equiv), $(NH_4)_2S_2O_8$ (0.15 g, 0.66 mmol, 3.0 equiv), benzaldehyde (0.023 g, 0.22 mmol, 1.0 equiv), 1.5 mL ethyl acetate, and 1.5 mL formamide. The reaction mixture was degassed via freeze pump thaw (× 3 times) and refilled with argon. After the reaction mixture was thoroughly degassed, the vial was sealed and positioned approximately 10 cm away from the light source. Two household full-spectrum 23 W CFL bulbs were used to irradiate the reaction mixture. Water bath was used to keep the reaction temperature at 29 °C. After stirring for the indicated time, the resulting mixture was diluted with ethyl acetate (30 mL), washed with brine (10 mL × 3), dried over Na_2SO_4, and concentrated under vacuum. Purification of the crude product by flash chromatography on silica gel using the indicated solvent system afforded the desired product.

Method C: To a 10 mL (or 20 mL) schlenk tube equipped with a magnetic stir bar was charged a heteroarene (0.22 mmol, 1.0 equiv), $(NH_4)_2S_2O_8$ (0.15 g, 0.66 mmol, 3.0 equiv), benzaldehyde (0.023 g, 0.22 mmol, 1.0 equiv), 1.5 mL amide, and 1.5 mL ethyl acetate. The reaction mixture was degassed via freeze pump thaw (× 3 times) and refilled with argon. After the reaction mixture was thoroughly degassed, the vial was sealed and positioned approximately 10 cm away from the light source. Two household full-spectrum 23 W CFL bulbs were used to irradiate the reaction mixture. Water bath was used to keep the reaction temperature at 29 °C. After stirring for the indicated time, the resulting mixture was diluted with ethyl acetate (30 mL), washed with brine (10 mL × 3), dried over Na_2SO_4, and concentrated under vacuum. Purification of the crude product by flash chromatography on silica gel using the indicated solvent system afforded the desired product.

Method D: To a 10 mL (or 20 mL) schlenk tube equipped with a magnetic stir bar was charged a heteroarene (0.22 mmol, 1.0 equiv), $(NH_4)_2S_2O_8$ (0.15 g, 0.66 mmol, 3.0 equiv), benzaldehyde (0.023 g, 0.22 mmol, 1.0 equiv), 1.5 mL ether, and 1.5 mL water. The reaction mixture was degassed via freeze pump thaw (× 3 times) and refilled with argon. After the reaction mixture was thoroughly degassed, the vial was sealed and positioned approximately 10 cm away from the light source. Two household full spectrum 23 W CFL bulbs were used to irradiate the reaction mixture. Water bath was used to keep the reaction temperature at 29 °C. After stirring for the indicated time, the resulting mixture was diluted with ethyl acetate (30 mL), washed with brine (10 mL × 3), dried over Na_2SO_4, and concentrated under vacuum. Purification of the crude product by flash chromatography on silica gel using the indicated solvent system afforded the desired product.

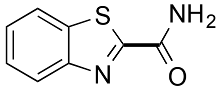

Benzo[d]thiazole-2-carboxamide (2a): According to the general procedure (method A), benzothiazole (0.030 g, 0.22 mmol, 1.0 equiv), $(NH_4)_2S_2O_8$ (0.15 g, 0.66 mmol, 3.0 equiv), TsOH•H_2O (0.042 g, 0.22 mmol, 1.0 equiv), benzaldehyde (0.023 g, 0.22 mmol, 1.0 equiv), 1.5 mL ethyl acetate, and 1.5 mL formamide were used. After 24 h, the reaction mixture was subjected to the workup protocol outlined in the general procedure and purified through flash chromatography (hexanes:acetone = 8:1–6:1) to provide the title compound as white solid (37 mg, 94%). ^{1}H NMR (500 MHz, d^6-DMSO) δ ppm 8.47 (brs, 1H), 8.19 (d, *J* = 8.0 Hz, 1H), 8.10 (d, *J* = 8.0 Hz, 1H), 8.07 (brs, 1H), 7.60 (t, *J* = 8.0 Hz, 1H), 7.55 (t, *J* = 8.0 Hz, 1H); ^{13}C NMR (125 MHz, d^6-DMSO) δ ppm 165.57, 162.00, 153.52, 137.06, 127.69, 127.52, 124.68, 123.66; HRMS (ESI) Calcd. for $C_8H_6N_2OS$ $(M + Na)^+$ 201.0099, found 201.0099.

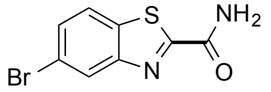

5-Bromobenzo[d]thiazole-2-carboxamide (2b): According to the general procedure (method A), 5-bromobenzothiazole (0.050 g, 0.23 mmol, 1.0 equiv), $(NH_4)_2S_2O_8$ (0.16 g, 0.69 mmol, 3.0

equiv), TsOH•H_2O (0.044 g, 0.23 mmol, 1.0 equiv), benzaldehyde (0.024 g, 0.23 mmol, 1.0 equiv), 2.5 mL ethyl acetate, and 2.5 mL formamide were used. After 24 h, the reaction mixture was subjected to the workup protocol outlined in the general procedure and purified through flash chromatography (hexanes:acetone = 8:1–6:1) to provide the title compound as white solid (50 mg, 85%). ^{1}H NMR (500 MHz, d^6-DMSO) δ ppm 8.50 (brs, 1H), 8.25 (d, *J* = 2.0 Hz, 1H), 8.16 (d, *J* = 8.5 Hz, 1H), 8.13 (brs, 1H), 7.70 (dd, *J* = 2.0, 8.0 Hz, 1H); ^{13}C NMR (125 MHz, d^6-DMSO) δ ppm 167.74, 161.64, 154.66, 136.25, 130.30, 126.86, 125.55, 120.43; HRMS (ESI) Calcd. for $C_8H_5BrN_2OS$ $(M + Na)^+$ 278.9204, found 278.9204.

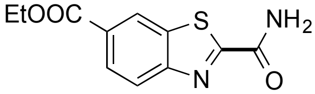

Ethyl 2-carbamoylbenzo[d]thiazole-6-carboxylate (2c): According to the general procedure (method A), ethyl benzo[d]thiazole-6-carboxylate (0.050 g, 0.24 mmol, 1.0 equiv), $(NH_4)_2S_2O_8$ (0.17 g, 0.72 mmol, 3.0 equiv), TsOH•H_2O (0.045 g, 0.24 mmol, 1.0 equiv), benzaldehyde (0.025 g, 0.24 mmol, 1.0 equiv), 2.5 mL ethyl acetate, and 2.5 mL formamide were used. After 24 h, the reaction mixture was subjected to the workup protocol outlined in the general procedure and purified through flash chromatography (hexanes:acetone = 8:1–6:1) to provide the title compound as white solid (30 mg, 50%). ^{1}H NMR (300 MHz, d^6-DMSO) δ ppm 8.89 (s, 1H), 8.59 (brs, 1H), 8.22 (d, *J* = 8.0 Hz, 1H), 8.18 (brs, 1H), 8.15 (d, *J* = 8.0 Hz, 1H), 4.38 (q, *J* = 7.2 Hz, 2H), 1.34 (t, *J* = 7.2 Hz, 3H); ^{13}C NMR (75 MHz, d^6-DMSO) δ ppm 169.45, 165.87, 161.69, 156.33, 137.25, 128.50, 128.02, 125.77, 124.72, 61.89, 14.85; HRMS (ESI) Calcd. for $C_{11}H_{10}N_2O_3S$ $(M + Na)^+$ 273.0310, found 273.0310.

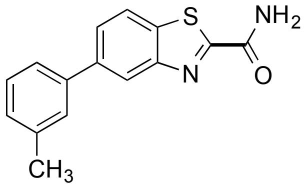

5-(*m*-tolyl)benzo[d]thiazole-2-carboxamide (2d): According to the general procedure (method A), 5-(m-tolyl)benzo[d]thiazole (0.050 g, 0.22 mmol, 1.0 equiv), $(NH_4)_2S_2O_8$ (0.15 g, 0.66 mmol, 3.0 equiv), TsOH•H_2O (0.042 g, 0.22 mmol, 1.0 equiv), benzaldehyde (0.023 g, 0.22 mmol, 1.0 equiv), 2.5 mL ethyl acetate, and 2.5 mL formamide were used. After 24 h, the reaction mixture was subjected to the workup protocol outlined in the general procedure and purified through flash chromatography (hexanes:acetone = 8:1–6:1) to provide the title compound as white solid (46 mg, 78%). ^{1}H NMR (500 MHz, d^6-DMSO) δ ppm 8.46 (brs, 1H), 8.27 (d, *J* = 2.0 Hz, 1H), 8.23 (d, *J* = 8.5 Hz, 1H), 8.13 (brs, 1H), 7.81 (dd, *J* = 2.0, 8.5 Hz, 1H), 7.55 (s, 1H), 7.51 (d, *J* = 8.0 Hz, 1H), 7.35 (t, *J* = 8.0 Hz, 1H), 7.18 (d, *J* = 7.5 Hz, 1H), 2.37 (s, 3H); ^{13}C NMR (125 MHz, d^6-DMSO) δ ppm 166.39, 161.95, 154.27, 140.25, 140.05, 138.94, 136.06, 129.63, 129.10, 128.45, 126.67, 124.89, 123.98, 122.11, 21.83; HRMS (ESI) Calcd. for $C_{15}H_{12}N_2OS$ $(M + Na)^+$ 291.0568, found 291.0573.

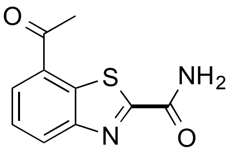

7-Acetylbenzo[d]thiazole-2-carboxamide (2e): According to the general procedure (method A), 1-(benzo[d]thiazol-7-yl)ethanone (0.050 g, 0.28 mmol, 1.0 equiv), $(NH_4)_2S_2O_8$ (0.19 g, 0.84 mmol, 3.0 equiv), TsOH•H_2O (0.053 g, 0.28 mmol, 1.0 equiv), benzaldehyde (0.030 g, 0.28 mmol, 1.0 equiv), 2.5 mL ethyl acetate, and 2.5 mL formamide were used. After 24 h, the reaction mixture was subjected to the workup protocol outlined in the general procedure and purified through flash chromatography (hexanes:acetone = 6:1–4:1) to provide the title compound as white solid (41 mg, 66%). ^{1}H NMR (300 MHz, d^6-DMSO) δ ppm 8.50 (brs, 1H), 8.39 (d, *J* = 8.1 Hz, 1H), 8.37 (d, *J* = 8.1 Hz, 1H), 8.08 (brs, 1H), 7.80 (t, *J* = 8.1 Hz, 1H), 2.76 (s, 3H); ^{13}C NMR (75 MHz, d^6-DMSO) δ ppm 197.62, 169.20, 162.33, 154.55, 134.28, 131.63, 130.47, 129.94, 128.00, 26.87; HRMS (ESI) Calcd. for $C_{10}H_8N_2O_2S$ $(M + Na)^+$ 243.0204, found 243.0204.

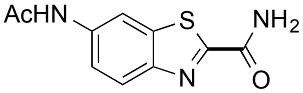

6-Acetamidobenzo[d]thiazole-2-carboxamide (2f): According to the general procedure (method A), *N*-(benzo[d]thiazol-6-yl)acetamide (0.040 g, 0.21 mmol, 1.0 equiv), $(NH_4)_2S_2O_8$ (0.14 g, 0.63 mmol, 3.0 equiv), TsOH•H_2O (0.040 g, 0.21 mmol, 1.0 equiv), benzaldehyde (0.022 g, 0.21 mmol, 1.0 equiv), 2.5 mL ethyl acetate, and 2.5 mL formamide were used. After 24 h, the reaction mixture was subjected to the workup protocol outlined in the general procedure and purified through flash chromatography (hexanes:acetone = 3:1–1:1) to provide the title compound as white solid (15 mg, 30%). ^{1}H NMR (500 MHz, d^6-DMSO) δ ppm 10.26 (s, 1H), 8.54 (d, *J* = 2.0 Hz, 1H), 8.34 (brs, 1H), 8.00 (d, *J* = 8.5 Hz, 1H), 7.95 (brs, 1H), 7.60 (dd, *J* = 2.0, 9.0 Hz, 1H), 2.08 (s, 3H); ^{13}C NMR (125 MHz, d^6-DMSO) δ ppm 169.40, 163.84, 162.06, 149.30, 138.89, 137.99, 124.79, 120.10, 111.94, 24.77; HRMS (ESI) Calcd. for $C_{10}H_9N_3O_2S$ $(M + Na)^+$ 258.0313, found 258.0314.

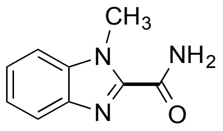

1-Methyl-1H-benzo[d]imidazole-2-carboxamide (2g): According to the general procedure (method A), 1-methyl-1H-benzo[d]imidazole (0.050 g, 0.38 mmol, 1.0 equiv), $(NH_4)_2S_2O_8$ (0.26 g, 1.13 mmol, 3.0 equiv), TsOH•H_2O (0.072 g, 0.38 mmol, 1.0 equiv), benzaldehyde (0.040 g, 0.38 mmol, 1.0 equiv), 2.5 mL ethyl acetate, and 2.5 mL formamide were used. After 24 h, the reaction mixture was subjected to the workup protocol outlined in the general procedure and purified through flash chromatography (hexanes:acetone = 5:1–3:1) to provide the title compound as white solid (42 mg, 64%). ^{1}H NMR (500 MHz, d^6-DMSO) δ ppm 8.26 (brs, 1H), 7.82 (brs, 1H), 7.71 (d, *J* = 8.0 Hz, 1H), 7.63 (d, *J* = 8.0 Hz, 1H), 7.36 (t, *J* = 8.0 Hz, 1H), 7.29 (t,

J = 8.0 Hz, 1H), 4.10 (s, 3H); ^{13}C NMR (125 MHz, d^{6}-DMSO) δ ppm 162.16, 144.57, 141.31, 137.34, 124.74, 123.51, 120.67, 111.91, 32.52; HRMS (ESI) Calcd. for $C_9H_9N_3O$ (M + Na)$^+$ 198.0643, found 198.0643.

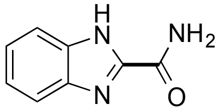

1H-benzo[d]imidazole-2-carboxamide (2h): According to the general procedure (method A), benzo[d]imidazole (0.050 g, 0.43 mmol, 1.0 equiv), $(NH_4)_2S_2O_8$ (0.30 g, 1.30 mmol, 3.0 equiv), TsOH•H_2O (0.081 g, 0.43 mmol, 1.0 equiv), benzaldehyde (0.045 g, 0.43 mmol, 1.0 equiv), 2.5 mL ethyl acetate, and 2.5 mL formamide were used. After 24 h, the reaction mixture was subjected to the workup protocol outlined in the general procedure and purified through flash chromatography (hexanes:acetone = 3:1–2:1) to provide the title compound as white solid (46 mg, 67%). ^{1}H NMR (500 MHz, d^{6}-DMSO) δ ppm 13.17 (s, 1H), 8.24 (brs, 1H), 7.80 (brs, 1H), 7.69 (d, J = 7.0 Hz, 1H), 7.50 (d, J = 7.0 Hz, 1H), 7.28 – 7.24 (m, 2H); ^{13}C NMR (125 MHz, d^{6}-DMSO) δ ppm 161.37, 146.49, 143.28, 135.20, 124.73, 123.12, 120.61, 113.16; HRMS (ESI) Calcd. for $C_8H_7N_3O$ (M + Na)$^+$ 184.0487, found 184.0489.

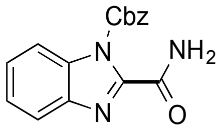

Benzyl 2-carbamoyl-1H-benzo[d]imidazole-1-carboxylate (2i): According to the general procedure (method A), benzyl 1H-benzo[d]imidazole-1-carboxylate (0.050 g, 0.20 mmol, 1.0 equiv), $(NH_4)_2S_2O_8$ (0.14 g, 0.60 mmol, 3.0 equiv), TsOH•H_2O (0.038 g, 0.20 mmol, 1.0 equiv), benzaldehyde (0.021 g, 0.20 mmol, 1.0 equiv), 2.5 mL ethyl acetate, and 2.5 mL formamide were used. After 24 h, the reaction mixture was subjected to workup protocol outlined in the general procedure and purified through flash chromatography (hexanes:acetone = 5:1–4:1) to provide the title compound as white solid (38 mg, 64%). ^{1}H NMR (500 MHz, d^{6}-DMSO) δ ppm 11.00 (brs, 1H), 7.80 – 7.50 (m, 2H), 7.47 (d, J = 7.0 Hz, 2H), 7.38 (t, J = 7.0 Hz, 2H), 7.34 – 7.31 (m, 3H); ^{13}C NMR (125 MHz, d^{6}-DMSO) δ ppm 157.99, 151.16, 144.56, 136.48, 129.06, 128.72, 128.38, 67.28; HRMS (ESI) Calcd. for $C_{16}H_{13}N_3O_3$ (M + Na)$^+$ 318.0855, found 318.0855.

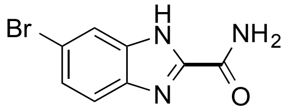

6-Bromo-1H-benzo[d]imidazole-2-carboxamide (2j): According to the general procedure (method A), 6-bromo-1H-benzo[d]imidazole (0.050 g, 0.25 mmol, 1.0 equiv), $(NH_4)_2S_2O_8$ (0.17 g, 0.75 mmol, 3.0 equiv), TsOH•H_2O (0.048 g, 0.25 mmol, 1.0 equiv), benzaldehyde (0.027 g, 0.25 mmol, 1.0 equiv), 2.5 mL ethyl acetate, and 2.5 mL formamide were used. After 24 h, the reaction mixture was subjected to the workup protocol outlined in the general procedure and purified through flash chromatography (hexanes:acetone = 3:1–2:1) to provide the title compound as white solid (51 mg, 85%). Both the Z and E isomers were obtained (E:Z = 1:1). ^{1}H

NMR (500 MHz, d^6-DMSO) δ ppm 13.40 (s, 1H, rotamer A), 13.33 (s, 1H, rotamer B), 8.29 (brs, 2H), 7.89 (s, 1H), 7.87 (brs, 2H), 7.66 (d, *J* = 7.5 Hz, 1H), 7.65 (s, 1H), 7.47 (d, *J* = 8.5 Hz, 1H), 7.42 (d, *J* = 8.5 Hz, 1H), 7.37 (d, *J* = 7.5 Hz, 1H); ^{13}C NMR (125 MHz, d^6-DMSO) δ ppm 161.51, 148.28, 147.96, 145.24, 142.92, 136.95, 134.83, 128.14, 126.79, 123.50, 123.04, 117.63, 116.29, 115.87, 115.61; HRMS (ESI) Calcd. for $C_8H_6BrN_3O$ (M + Na)$^+$ 261.9592, found 261.9598.

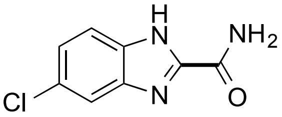

5-Chloro-1H-benzo[d]imidazole-2-carboxamide (2k): According to the general procedure (method A), 5-chloro-1H-benzo[d]imidazole (0.050 g, 0.33 mmol, 1.0 equiv), $(NH_4)_2S_2O_8$ (0.23 g, 0.99 mmol, 3.0 equiv), TsOH•H_2O (0.063 g, 0.33 mmol, 1.0 equiv), benzaldehyde (0.035 g, 0.33 mmol, 1.0 equiv), 2.5 mL ethyl acetate, and 2.5 mL formamide were used. After 24 h, the reaction mixture was subjected to the workup protocol outlined in the general procedure and purified through flash chromatography (hexanes:acetone = 3:1–2:1) to provide the title compound as white solid (50 mg, 78%). Both the Z and E isomers were obtained (E:Z = 1:1). ^{1}H NMR (500 MHz, d^6-DMSO) δ ppm 13.40 (s, 1H, rotamer A), 13.33 (s, 1H, rotamer B), 8.30 (brs, 1H), 8.28 (brs, 1H), 7.88 (brs, 2H), 7.54 (s, 1H), 7.71 (d, *J* = 8.5 Hz, 1H), 7.52 (d, *J* = 9.0 Hz, 1H), 7.51 (s, 1H), 7.31 (d, *J* = 8.5 Hz, 1H), 7.26 (d, *J* = 9.0 Hz, 1H); ^{13}C NMR (125 MHz, d^6-DMSO) δ ppm 160.74, 147.71, 147.37, 143.88, 141.88, 135.66, 133.76, 128.87, 127.27, 124.82, 123.41, 121.86, 119.68, 114.39, 112.52; HRMS (ESI) Calcd. for $C_8H_6ClN_3O$ (M + Na)$^+$ 218.0097, found 218.0102.

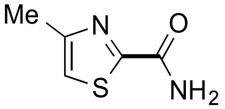

4-Methylthiazole-2-carboxamide (2m): According to the general procedure (method A), 5-chloro-1H-benzo[d]imidazole (0.050 g, 0.50 mmol, 1.0 equiv), $(NH_4)_2S_2O_8$ (0.34 g, 1.50 mmol, 3.0 equiv), TsOH•H_2O (0.095 g, 0.50 mmol, 1.0 equiv), benzaldehyde (0.053 g, 0.50 mmol, 1.0 equiv), 2.5 mL ethyl acetate, and 2.5 mL formamide were used. After 24 h, the reaction mixture was subjected to the workup protocol outlined in the general procedure and purified through flash chromatography (hexanes:acetone = 8:1–6:1) to provide the title compound as white solid (38 mg, 54%). ^{1}H NMR (500 MHz, d^6-DMSO) δ ppm 8.06 (brs, 1H), 7.76 (brs, 1H), 7.55 (s, 1H), 2.40 (s, 3H); ^{13}C NMR (125 MHz, d^6-DMSO) δ ppm 164.02, 161.64, 154.18, 121.05, 17.48; HRMS (ESI) Calcd. for $C_5H_6N_2OS$ (M + Na)$^+$ 165.0099, found 165.0101.

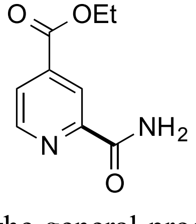

Ethyl isonicotinate (4a): According to the general procedure (method A), ethyl 9H-pyrido[3,4-

b]indole-3-carboxylate (0.050 g, 0.33 mmol, 1.0 equiv), $(NH_4)_2S_2O_8$ (0.23 g, 0.99 mmol, 3.0 equiv), TsOH•H_2O (0.063 g, 0.33 mmol, 1.0 equiv), benzaldehyde (0.035 g, 0.33 mmol, 1.0 equiv), 2.5 mL ethyl acetate, and 2.5 mL formamide were used. After 24 h, the reaction mixture was subjected to the workup protocol outlined in the general procedure and purified through flash chromatography (hexanes:acetone = 5:1–3:1) to provide the title compound as white solid (43 mg, 67%). ^{1}H NMR (500 MHz, d^6-DMSO) δ ppm 8.81 (dd, J = 0.5, 5.0 Hz, 1H), 8.39 (dd, J = 0.5, 1.5 Hz, 1H), 8.23 (brs, 1H), 7.98 (dd, J = 1.5, 5.5 Hz, 1H), 7.81 (brs, 1H), 4.35 (q, J = 7.0 Hz, 2H), 1.33 (t, J = 7.0 Hz, 3H); ^{13}C NMR (125 MHz, d^6-DMSO) δ ppm 165.88, 164.81, 152.16, 150.51, 139.22, 125.73, 121.21, 62.53, 14.65; HRMS (ESI) Calcd. for $C_9H_{10}N_2O_3$ $(M + Na)^+$ 217.0589, found 217.0586.

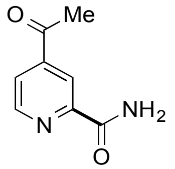

4-Acetylpicolinamide (4b): According to the general procedure (method A), 4-acetylpyridine (0.050 g, 0.41 mmol, 1.0 equiv), $(NH_4)_2S_2O_8$ (0.28 g, 1.24 mmol, 3.0 equiv), TsOH•H_2O (0.078 g, 0.41 mmol, 1.0 equiv), benzaldehyde (0.043 g, 0.41 mmol, 1.0 equiv), 2.5 mL ethyl acetate, and 2.5 mL formamide were used. After 24 h, the reaction mixture was subjected to the workup protocol outlined in the general procedure and purified through flash chromatography (hexanes:acetone = 5:1–3:1) to provide the title compound as white solid (40 mg, 60%). ^{1}H NMR (300 MHz, d^6-DMSO) δ ppm 8.83 (d, J = 5.1 Hz, 1H), 8.36 (s, 1H), 8.23 (brs, 1H), 7.98 (dd, J = 1.5, 4.8 Hz, 1H), 7.81 (brs, 1H), 2.66 (s, 3H); ^{13}C NMR (75 MHz, d^6-DMSO) δ ppm 198.29, 166.12, 152.24, 150.53, 144.63, 124.55, 120.06, 17.71; HRMS (ESI) Calcd. for $C_8H_8N_2O_2$ $(M + Na)^+$ 187.0483, found 187.0482.

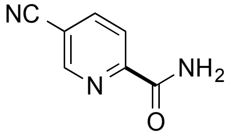

5-Cyanopicolinamide (4c): According to the general procedure (method A), 5-cyanopyridine (0.050 g, 0.48 mmol, 1.0 equiv), $(NH_4)_2S_2O_8$ (0.40 g, 1.44 mmol, 3.0 equiv), TsOH•H_2O (0.091 g, 0.48 mmol, 1.0 equiv), benzaldehyde (0.051 g, 0.48 mmol, 1.0 equiv), 2.5 mL ethyl acetate, and 2.5 mL formamide were used. After 24 h, the reaction mixture was subjected to the workup protocol outlined in the general procedure and purified through flash chromatography (hexanes:acetone = 5:1–3:1) to provide the title compound as white solid (46 mg, 65%). Regioselectivity ratio (r.r.), C-6:C-2:C-4 = 16.5:5.5:1. Major isomer (C-2): ^{1}H NMR (300 MHz, d^6-DMSO) δ ppm 9.08 (dd, J = 0.9, 2.1 Hz, 1H), 8.48 (dd, J = 2.1, 8.4 Hz, 1H), 8.31 (brs, 1H), 8.15 (dd, J = 0.9, 8.1 Hz, 1H), 7.92 (brs, 1H); Minor isomer (C-6): ^{1}H NMR (300 MHz, d^6-DMSO) δ ppm 8.85 (dd, J = 1.5, 4.8 Hz, 1H), 8.43 (dd, J = 1.5, 7.8 Hz, 1H), 8.31 (brs, 1H), 7.96 (brs, 1H), 7.77 (dd, J = 4.8, 8.1 Hz, 1H); isomers, C-6 and C-2, ^{13}C NMR (75 MHz, d^6-DMSO) δ ppm 165.35, 164.95, 153.51, 152.66, 152.26, 152.23, 144.31, 142.49, 126.79, 122.57, 117.37,

117.23; HRMS (ESI) Calcd. for $C_7H_5N_3O$ $(M + Na)^+$ 170.0330, found 170.0336.

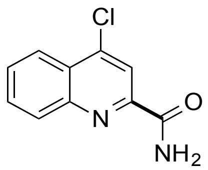

4-Chloroquinoline-2-carboxamide (4d): According to the general procedure (method B), 4-chloroquinoline (0.030 g, 0.18 mmol, 1.0 equiv), $(NH_4)_2S_2O_8$ (0.12 g, 0.54 mmol, 3.0 equiv), benzaldehyde (0.019 g, 0.18 mmol, 1.0 equiv), 1.5 mL ethyl acetate, and 1.5 mL formamide were used. After 6 h, the reaction mixture was subjected to the workup protocol outlined in the general procedure and purified through flash chromatography (hexanes:acetone = 10:1–8:1) to provide the title compound as white solid (35 mg, 95%). ^1H NMR (300 MHz, d^6-DMSO) δ ppm 8.35 (brs, 1H), 8.23 (d, J = 8.4 Hz, 1H), 8.20 (s, 1H), 8.16 (d, J = 8.4 Hz, 1H), 7.95 (t, J = 8.4 Hz, 1H), 7.91 (brs, 1H), 7.84 (t, J = 8.4 Hz, 1H); ^{13}C NMR (75 MHz, d^6-DMSO) δ ppm 165.77, 151.28, 147.63, 143.47, 132.30, 130.64, 130.34, 126.89, 124.42, 119.42; HRMS (ESI) Calcd. for $C_{10}H_7ClN_2O$ $(M + Na)^+$ 229.0145, found 229.0142.

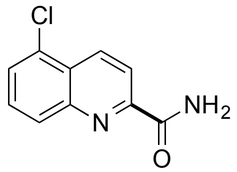

5-Chloroquinoline-2-carboxamide (4e): According to the general procedure (method B), 5-chloroquinoline (0.030 g, 0.18 mmol, 1.0 equiv), $(NH_4)_2S_2O_8$ (0.12 g, 0.54 mmol, 3.0 equiv), benzaldehyde (0.019 g, 0.18 mmol, 1.0 equiv), 1.5 mL ethyl acetate, and 1.5 mL formamide were used. After 12 h, the reaction mixture was subjected to the workup protocol outlined in the general procedure and purified through flash chromatography (hexanes:acetone = 10:1–8:1) to provide the title compound as white solid (35 mg, 95%). ^1H NMR (500 MHz, d^6-DMSO) δ ppm 8.71 (d, J = 8.5 Hz, 1H), 8.33 (brs, 1H), 8.26 (d, J = 9.0 Hz, 1H), 8.09 (d, J = 8.0 Hz, 1H), 7.88 (d, J = 7.5 Hz, 1H), 7.87 (brs, 1H), 7.84 (t, J = 7.5 Hz, 1H); ^{13}C NMR (125 MHz, d^6-DMSO) δ ppm 166.36, 151.91, 147.50, 134.78, 131.25, 130.99, 129.67, 128.90, 127.03, 120.65; HRMS (ESI) Calcd. for $C_{10}H_7ClN_2O$ $(M + Na)^+$ 229.0145, found 229.0145.

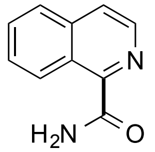

Isoquinoline-1-carboxamide (4f): According to the general procedure (method B), isoquinoline (0.050 g, 0.39 mmol, 1.0 equiv), $(NH_4)_2S_2O_8$ (0.26 g, 1.16 mmol, 3.0 equiv), benzaldehyde (0.041 g, 0.39 mmol, 1.0 equiv), 2.5 mL ethyl acetate, and 2.5 mL formamide were used. After 12 h, the reaction mixture was subjected to the workup protocol outlined in the general procedure and purified through flash chromatography (hexanes:acetone = 8:1–5:1) to provide the title compound as white solid (58 mg, 87%). ^1H NMR (300 MHz, d^6-DMSO) δ ppm 8.94 (d, J = 8.4

Hz, 1H), 8.50 (d, J = 5.7 Hz, 1H), 8.26 (brs, 1H), 8.00 (d, J = 8.1 Hz, 1H), 7.96 (d, J = 5.4 Hz, 1H), 7.80 (brs, 1H), 7.78 (t, J = 8.4 Hz, 1H), 7.69 (t, J = 8.4 Hz, 1H); ^{13}C NMR (75 MHz, d^6-DMSO) δ ppm 169.16, 152.06, 141.51, 137.19, 131.24, 128.96, 127.75, 127.40, 126.12, 123.92; HRMS (ESI) Calcd. for $C_{10}H_8N_2O$ (M + Na)$^+$ 195.0534, found 195.0536.

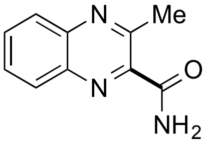

3-Methylquinoxaline-2-carboxamide (4g): According to the general procedure (method B), 2-methylquinoxaline (0.030 g, 0.21 mmol, 1.0 equiv), $(NH_4)_2S_2O_8$ (0.14 g, 0.63 mmol, 3.0 equiv), benzaldehyde (0.022 g, 0.21 mmol, 1.0 equiv), 1.5 mL ethyl acetate, and 1.5 mL formamide were used. After 24 h, the reaction mixture was subjected to the workup protocol outlined in the general procedure and purified through flash chromatography (hexanes:acetone = 8:1–5:1) to provide the title compound as white solid (28 mg, 71%). ^{1}H NMR (500 MHz, d^6-DMSO) δ ppm 8.24 (brs, 1H), 8.07 (d, J = 8.0 Hz, 1H), 8.03 (d, J = 8.0 Hz, 1H), 7.88 (brs, 1H), 7.87 (t, J = 8.0 Hz, 1H), 7.82 (t, J = 8.0 Hz, 1H), 2.81 (s, 3H); ^{13}C NMR (125 MHz, d^6-DMSO) δ ppm 189.54, 168.37, 152.87, 148.24, 142.23, 139.52, 131.83, 130.51, 129.55, 128.80; HRMS (ESI) Calcd. for $C_{10}H_9N_3O$ (M + Na)$^+$ 210.0643, found 210.0644.

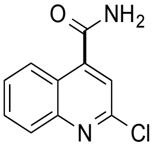

2-Chloroquinoline-4-carboxamide (4h): According to the general procedure (method B), 2-chloroquinoline (0.030 g, 0.18 mmol, 1.0 equiv), $(NH_4)_2S_2O_8$ (0.12 g, 0.54 mmol, 3.0 equiv), benzaldehyde (0.019 g, 0.18 mmol, 1.0 equiv), 1.5 mL ethyl acetate, and 1.5 mL formamide were used. After 12 h, the reaction mixture was subjected to the workup protocol outlined in the general procedure and purified through flash chromatography (hexanes:acetone = 5:1–3:1) to provide the title compound as white solid (6 mg, 15%). ^{1}H NMR (500 MHz, d^6-DMSO) δ ppm 8.32 (brs, 1H), 8.18 (d, J = 8.5 Hz, 1H), 8.02 (brs, 1H), 7.99 (d, J = 8.5 Hz, 1H), 7.85 (t, J = 8.5 Hz, 1H), 7.70 (t, J = 8.5 Hz, 1H), 7.62 (s, 1H); ^{13}C NMR (125 MHz, d^6-DMSO) δ ppm 167.71, 150.05, 148.33, 146.57, 131.88, 128.97, 128.57, 126.44, 123.89, 120.56; HRMS (ESI) Calcd. for $C_{10}H_7ClN_2O$ (M + Na)$^+$ 229.0145, found 229.0138.

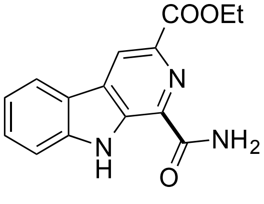

Ethyl 1-carbamoyl-9H-pyrido[3,4-b]indole-3-carboxylate (4i): According to the general procedure (method A), ethyl 9H-pyrido[3,4-b]indole-3-carboxylate (0.050 g, 0.21 mmol, 1.0 equiv), $(NH_4)_2S_2O_8$ (0.14 g, 0.63 mmol, 3.0 equiv), TsOH•H_2O (0.039 g, 0.21 mmol, 1.0 equiv), benzaldehyde (0.022 g, 0.21 mmol, 1.0 equiv), 2.5 mL ethyl acetate, and 2.5 mL formamide

were used. After 24 h, the reaction mixture was subjected to the workup protocol outlined in the general procedure and purified through flash chromatography (CH_2Cl_2:MeOH = 80:1–50:1) to provide the title compound as pale yellow solid (30 mg, 50%). Both the Z and E isomers were obtained. (E:Z = 1:1). ^{1}H NMR (500 MHz, d^6-DMSO) δ ppm 12.48 (s, 1H, rotamer A), 12.07 (s, 1H, rotamer B), 11.29 (brs, 1H), 9.06 (s, 1H), 8.42 (d, *J* = 8.0 Hz, 1H), 8.18 (d, *J* = 8.0 Hz, 1H), 7.98 (d, J = 9.0 Hz, 2H), 7.90 (s, 1H), 7.85 (d, *J* = 8.5 Hz, 1H), 7.60 (t, *J* = 7.5 Hz, 1H), 7.55 (d, *J* = 8.0 Hz, 1H), 7.44 (t, *J* = 8.0 Hz, 1H), 7.31 (t, *J* = 7.5 Hz, 1H), 7.24 (t, *J* = 7.5 Hz, 1H), 4.40 (q, *J* = 7.0 Hz, 2H), 4.33 (q, *J* = 7.0 Hz, 2H), 1.39 (t, *J* = 7.0 Hz, 3H), 1.34 (t, *J* = 7.0 Hz, 3H); ^{13}C NMR (125 MHz, d^6-DMSO) δ ppm 167.55, 165.30, 161.98, 155.19, 142.49, 139.82, 136.09, 135.67, 132.62, 131.53, 131.39, 129.69, 127.21, 125.31, 123.01, 122.63, 122.47, 122.05, 121.09, 120.74, 120.43, 113.89, 113.28, 106.57, 61.88, 61.38, 14.78, 14.60; HRMS (ESI) Calcd. for $C_{15}H_{13}N_3O_3$ (M + Na)$^+$ 306.0855, found 306.0861.

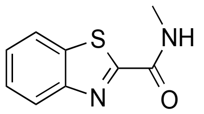

***N*-Methylbenzo[d]thiazole-2-carboxamide (5a):** According to the general procedure (method C), benzothiazole (0.030 g, 0.22 mmol, 1.0 equiv), $(NH_4)_2S_2O_8$ (0.15 g, 0.66 mmol, 3.0 equiv), benzaldehyde (0.023 g, 0.22 mmol, 1.0 equiv), 1.5 mL ethyl acetate, and 1.5 mL *N*-methylformamide were used. After 24 h, the reaction mixture was subjected to the workup protocol outlined in the general procedure and purified through flash chromatography (hexanes:acetone = 8:1–6:1) to provide the title compound as colorless oil (37 mg, 88%). ^{1}H NMR (500 MHz, d^6-Acetone) δ ppm 8.25 (brs, 1H), 8.15 (d, *J* = 8.0 Hz, 1H), 8.05 (d, *J* = 8.5 Hz, 1H), 7.59 (t, *J* = 8.0 Hz, 1H), 7.55 (t, *J* = 8.0 Hz, 1H), 3.03 (d, *J* = 5.0 Hz, 3H); ^{13}C NMR (125 MHz, d^6-Acetone) δ ppm 164.91, 160.24, 153.41, 136.97, 127.03, 126.89, 124.88, 122.82, 25.87; HRMS (ESI) Calcd. for $C_9H_8N_2OS$ (M + Na)$^+$ 215.0255, found 215.0255.

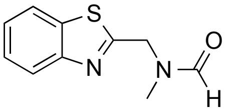

***N*-(Benzo[d]thiazol-2-ylmethyl)-*N*-methylformamide (6a):** According to the general procedure (method C), benzothiazole (0.030 g, 0.22 mmol, 1.0 equiv), $(NH_4)_2S_2O_8$ (0.15 g, 0.66 mmol, 3.0 equiv), benzaldehyde (0.023 g, 0.22 mmol, 1.0 equiv), 1.5 mL ethyl acetate, and 1.5 mL *N*, *N*-dimethylformamide were used. After 24 h, the reaction mixture was subjected to the workup protocol outlined in the general procedure and purified through flash chromatography (hexanes:acetone = 5:1–3:1) to provide the title compound as colorless oil (36 mg, 80%). Both the Z and E isomers were obtained. (E:Z = 1:1) ^{1}H NMR (500 MHz, d^6-Acetone) δ ppm 8.38 (s, 1H, rotamer A), 8.22 (s, 1H, rotamer B), 8.05 (d, *J* = 8.5 Hz, 1H), 8.02 (d, *J* = 8.0 Hz, 1H), 7.99 (d, *J* = 8.5 Hz, 1H), 7.97 (d, *J* = 8.5 Hz, 1H), 7.54 (t, *J* = 8.5 Hz, 1H), 7.51 (t, *J* = 8.5 Hz, 1H), 7.46 (t, *J* = 8.5 Hz, 1H), 7.43 (t, *J* = 8.5 Hz, 1H), 4.97 (s, 2H), 4.91 (s, 2H), 3.12 (s, 3H), 2.92 (s, 3H); ^{13}C NMR (125 MHz, d^6-Acetone) δ ppm 168.78, 167.56, 162.92, 162.65, 153.71, 153.40, 135.74, 135.50, 126.49, 126.29, 125.61, 125.45, 123.17, 123.02, 122.30, 122.17, 51.05, 45.84, 34.07, 29.52; HRMS (ESI) Calcd. for $C_{10}H_{10}N_2OS$ (M + Na)$^+$ 229.0412, found 229.0412.

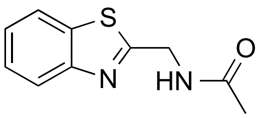

***N*-(Benzo[d]thiazol-2-ylmethyl)acetamide (6b):** According to the general procedure (method C), benzothiazole (0.060 g, 0.44 mmol, 1.0 equiv), $(NH_4)_2S_2O_8$ (0.30 g, 1.32 mmol, 3.0 equiv), benzaldehyde (0.046 g, 0.44 mmol, 1.0 equiv), 2.5 mL ethyl acetate, and 2.5 mL *N*-methylformamide were used. After 24 h, the reaction mixture was subjected to the workup protocol outlined in the general procedure and purified through flash chromatography (hexanes:acetone = 3:1–2:1) to provide the title compound as white solid (74 mg, 82%). ^{1}H NMR (300 MHz, d^6-DMSO) δ ppm 8.86 (t, *J* = 6.0 Hz, 1H), 8.04 (d, *J* = 7.8 Hz, 1H), 7.92 (d, *J* = 7.8 Hz, 1H), 7.49 (t, *J* = 7.8 Hz, 1H), 7.39 (t, *J* = 7.8 Hz, 1H), 4.63 (d, *J* = 6.0 Hz, 2H), 1.92 (s, 3H); ^{13}C NMR (75 MHz, d^6-DMSO) δ ppm 172.23, 170.51, 153.40, 135.23, 126.79, 125.65, 123.01, 122.91, 41.74, 23.08; HRMS (ESI) Calcd. for $C_{10}H_{10}N_2OS$ $(M + Na)^+$ 229.0412, found 229.0408.

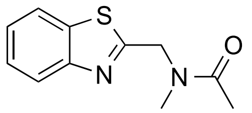

***N*-(benzo[d]thiazol-2-ylmethyl)-*N*-methylacetamide (6c):** According to the general procedure (method C), benzothiazole (0.030 g, 0.22 mmol, 1.0 equiv), $(NH_4)_2S_2O_8$ (0.15 g, 0.66 mmol, 3.0 equiv), benzaldehyde (0.023 g, 0.22 mmol, 1.0 equiv), 1.5 mL ethyl acetate, and 1.5 mL *N*, *N*-dimethylacetamide were used. After 24 h, the reaction mixture was subjected to the workup protocol outlined in the general procedure and purified through flash chromatography (hexanes:acetone = 5:1–3:1) to provide the title compound as colorless oil (42 mg, 87%). Both the Z and E isomers were obtained (E:Z = 2.4:1). Major isomer, ^{1}H NMR (500 MHz, d^6-Acetone) δ ppm 8.00 (d, *J* = 8.0 Hz, 1H), 7.95 (d, *J* = 8.0 Hz, 1H), 7.50 (t, *J* = 8.0 Hz, 1H), 7.41 (t, *J* = 8.0 Hz, 1H), 4.92 (s, 2H), 3.17 (s, 3H), 2.13 (s, 3H); ^{13}C NMR (125 MHz, d^6-Acetone) δ ppm 170.36, 169.05, 153.4, 135.78, 126.17, 125.29, 122.92, 122.12, 49.18, 36.04, 20.92; HRMS (ESI) Calcd. for $C_{11}H_{12}N_2OS$ $(M + Na)^+$ 243.0568, found 243.0566.

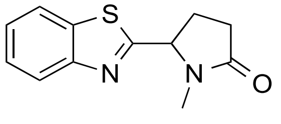

5-(Benzo[d]thiazol-2-yl)-1-methylpyrrolidin-2-one [(±)-6d]: According to the general procedure (method C), benzothiazole (0.030 g, 0.22 mmol, 1.0 equiv), $(NH_4)_2S_2O_8$ (0.15 g, 0.66 mmol, 3.0 equiv), benzaldehyde (0.023 g, 0.22 mmol, 1.0 equiv), 1.5 mL ethyl acetate, and 1.5 mL 1-methylpyrrolidin-2-one were used. After 24 h, the reaction mixture was subjected to the workup protocol outlined in the general procedure and purified through flash chromatography (hexanes:acetone = 5:1–3:1) to provide the title compound as colorless oil (45 mg, 89%). ^{1}H NMR (300 MHz, d^6-Acetone) δ ppm 8.06 (d, *J* = 8.1 Hz, 1H), 8.00 (d, *J* = 7.8 Hz, 1H), 7.54 (t, *J* = 7.8 Hz, 1H), 7.45 (t, *J* = 7.8 Hz, 1H), 5.09 (dd, *J* = 3.9, 8.7 Hz, 1H), 2.80 (s, 3H), 2.74 – 2.05 (m, 4H); ^{13}C NMR (75 MHz, d^6-Acetone) δ ppm 174.32, 173.19, 153.61, 135.18, 126.53, 125.68, 123.27, 122.47, 62.28, 28.23, 26.61; HRMS (ESI) Calcd. for $C_{12}H_{12}N_2OS$ $(M + Na)^+$ 255.0568,

found 255.0564.

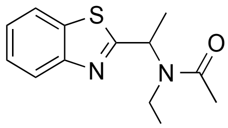

***N*-(1-(Benzo[d]thiazol-2-yl)ethyl)-*N*-ethylacetamide [(±)-6e]:** According to the general procedure (method C), benzothiazole (0.060 g, 0.44 mmol, 1.0 equiv), $(NH_4)_2S_2O_8$ (0.30 g, 1.32 mmol, 3.0 equiv), TsOH•H_2O (0.081 g, 0.44 mmol, 1.0 equiv), benzaldehyde (0.046 g, 0.44 mmol, 1.0 equiv), 2.5 mL ethyl acetate, and 2.5 mL *N*, *N*-dimethylacetamide were used. After 24 h, the reaction mixture was subjected to the workup protocol outlined in the general procedure and purified through flash chromatography (hexanes:acetone = 8:1–6:1) to provide the title compound as pale yellow oil (28 mg, 25%). Both the Z and E isomers were obtained (E:Z = 2.5:1). Major isomer, ^{1}H NMR (500 MHz, d^6-Acetone) δ ppm 7.99 (d, *J* = 8.0 Hz, 1H), 7.96 (d, *J* = 8.0 Hz, 1H), 7.49 (t, *J* = 8.0 Hz, 1H), 7.41 (t, *J* = 8.0 Hz, 1H), 5.95 (q, *J* = 7.5 Hz, 1H), 3.51 (dt, *J* = 7.5, 23.0 Hz, 1H), 3.35 (dt, *J* = 7.5, 23.0 Hz, 1H), 2.15 (s, 3H), 1.74 (d, *J* = 7.0 Hz, 3H), 1.16 (t, *J* = 7.0 Hz, 3H); ^{13}C NMR (125 MHz, d^6-Acetone) δ ppm 173.32, 169.96, 153.24, 135.79, 126.07, 125.30, 122.99, 122.03, 51.87, 40.35, 21.21, 16.79, 15.67; HRMS (ESI) Calcd. for $C_{13}H_{16}N_2OS$ $(M + Na)^+$ 271.0881, found 271.0884.

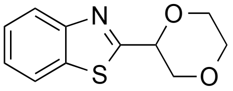

2-(1,4-Dioxan-2-yl)benzo[d]thiazole [(±)-7a]: According to the general procedure (method D), benzothiazole (0.060 g, 0.44 mmol, 1.0 equiv), $(NH_4)_2S_2O_8$ (0.30 g, 1.32 mmol, 3.0 equiv), benzaldehyde (0.046 g, 0.44 mmol, 1.0 equiv), 2.5 mL 1,4-dioxane, and 2.5 mL water were used. After 48 h, the reaction mixture was subjected to the workup protocol outlined in the general procedure and purified through flash chromatography (hexanes:ethyl acetate = 30:1–20:1) to provide the title compound as colorless oil (60 mg, 62%). ^{1}H NMR (500 MHz, d^6-Acetone) δ ppm 8.07 (d, *J* = 8.0 Hz, 1H), 7.98 (d, *J* = 8.0 Hz, 1H), 7.52 (t, *J* = 8.0 Hz, 1H), 7.44 (t, *J* = 8.0 Hz, 1H), 5.01 (dd, *J* = 3.0, 9.5 Hz, 1H), 4.21 (dd, *J* = 3.0, 11.5 Hz, 1H), 3.99 (dd, *J* = 3.0, 12.0 Hz, 1H), 3.94 – 3.91 (m, 1H), 3.82 – 3.79 (m, 1H), 3.71 – 3.66 (m, 1H), 3.63 (dd, *J* = 9.5, 11.5 Hz, 1H); ^{13}C NMR (125 MHz, d^6-Acetone) δ ppm 169.65, 153.59, 134.84, 126.28, 125.33, 123.10, 122.22, 75.39, 70.25, 67.00, 66.33; HRMS (ESI) Calcd. for $C_{11}H_{11}NO_2S$ $(M + Na)^+$ 244.0408, found 244.0409.

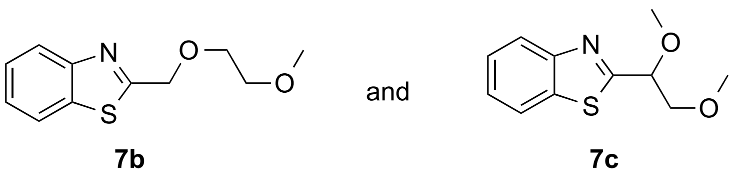

2-((2-Methoxyethoxy) methyl) benzo[d]thiazole (7b) and 2-(1,2-dimethoxyethyl) benzo[d]thiazole (7c): According to the general procedure (method D), benzothiazole (0.060 g, 0.44 mmol, 1.0 equiv), $(NH_4)_2S_2O_8$ (0.30 g, 1.32 mmol, 3.0 equiv), benzaldehyde (0.046 g, 0.44

mmol, 1.0 equiv), 2.5 mL glycol dimethyl ether, and 2.5 mL water were used. After 48 h, the reaction mixture was subjected to the workup protocol outlined in the general procedure and purified through flash chromatography (hexanes:ethyl acetate = 15:1, 10:1 to 4:1) to provide **7b** and **7c** as colorless oil (62 mg, 68%) with a ratio of 1:1 (regioselectivity ratio). **7b:** ^{1}H NMR (500 MHz, d^{6}-Acetone) δ ppm 8.05 (d, *J* = 8.0 Hz, 1H), 7.95 (d, *J* = 8.0 Hz, 1H), 4.95 (s, 2H), 3.80 (t, *J* = 4.5 Hz, 2H), 3.60 (t, *J* = 4.5 Hz, 2H), 3.34 (s, 3H); ^{13}C NMR (125 MHz, d^{6}-Acetone) δ ppm 170.73, 153.57, 135.26, 126.16, 125.19, 122.98, 122.17, 71.93, 70.84, 70.57, 58.27; HRMS (ESI) Calcd for $C_{11}H_{13}NO_2S$ $(M+Na)^+$ 246.0565, found 246.0558. **7c:** ^{1}H NMR (500 MHz, d^{6}-Acetone) δ ppm 8.06 (d, *J* = 8.0 Hz, 1H), 7.97 (d, *J* = 8.0 Hz, 1H), 7.51 (t, *J* = 8.0 Hz, 1H), 7.44 (t, *J* = 8.0 Hz, 1H), 4.83 (dd, *J* = 4.0, 6.5 Hz, 1H), 3.84 (dd, *J* = 4.0, 10.5 Hz, 1H), 3.75 (dd, *J* = 6.5, 10.5 Hz, 1H), 3.51 (s, 3H), 3.62 (s, 3H); ^{13}C NMR (125 MHz, d^{6}-Acetone) δ ppm 172.03, 153.61, 135.33, 126.15, 125.30, 123.09, 122.23, 81.19, 75.30, 58.63, 57.94; HRMS (ESI) Calcd for $C_{11}H_{13}NO_2S$ $(M+Na)^+$ 246.0565, found 246.0560.

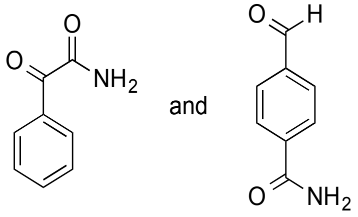

2-Oxo-2-phenylacetamide (8a) and 4-formylbenzamide (8b): According to the general procedure (method B), $(NH_4)_2S_2O_8$ (0.30 g, 1.32 mmol, 1.0 equiv), benzaldehyde (0.14 g, 1.32 mmol, 1.0 equiv), 1.5 mL ethyl acetate, and 1.5 mL formamide were mixed. After 24 h, the reaction mixture was subjected to the workup protocol outlined in the general procedure and purified through flash chromatography (hexanes:acetone = 10:1–5:1–3:1) to provide the title compounds **12** (8 mg, 4%) and **13** (5 mg, 2.5%) as white solid. Compound **8a**: ^{1}H NMR (300 MHz, d^{6}-DMSO) δ ppm 8.32 (brs, 1H), 8.01 (brs, 1H), 7.95 (d, *J* = 7.2 Hz, 2H), 7.72 (t, *J* = 7.2 Hz, 1H), 7.57 (t, *J* = 7.2 Hz, 2H); ^{13}C NMR (75 MHz, d^{6}-DMSO) δ ppm 191.52, 167.85, 135.16, 133.41, 130.30, 129.64; GCMS (EI) acquisition time, 15.86 min, m/z = 149.0 (5.23%), m/z = 104.9 (100%), m/z = 77.0 (53.85%). Compound **8b**: ^{1}H NMR (300 MHz, d^{6}-DMSO) δ ppm 10.06 (s, 1H), 8.18 (brs, 1H), 8.04 (d, *J* = 8.1 Hz, 2H), 7.97 (d, *J* = 8.1 Hz, 2H), 7.61 (brs, 1H); GCMS (EI) acquisition time, 18.19 min, m/z = 148.9 (91.79%), m/z = 132.9 (100%), m/z = 77.0 (33.44%).

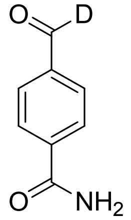

Deuterated-4-formylbenzamide (9). It was prepared by the same procedure as compound **8b**, white solid (4 mg, 2.0%). ^{1}H NMR (300 MHz, d^{6}-DMSO) *δ* ppm 8.16 (brs, 1H), 8.03 (d, *J* = 8.1 Hz, 2H), 7.97 (d, *J* = 8.1 Hz, 2H), 7.60 (brs, 1H); GCMS (EI) acquisition time, 18.19 min, m/z =

149.9 (89.73%), m/z = 133.9 (100%), m/z = 78.0 (26.41/%).

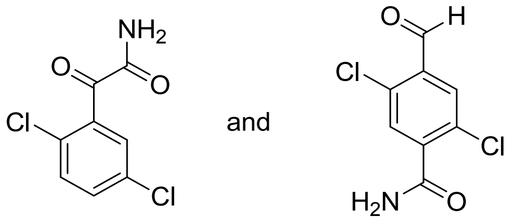

2-(2,5-Dichlorophenyl)-2-oxoacetamide (10a) and 2,5-dichloro-4-formylbenzamide (10b). These two compounds were prepared through the same procedure as compounds **8a** and **8b**. Compound **10a**, white solid (28.2 mg, 19%). ^{1}H NMR (500 MHz, d^{6}-DMSO) δ ppm 8.40 (brs, 1H), 8.05 (brs, 1H), 7.72 (d, J = 3.0 Hz, 1H), 7.66 (dd, J = 3.0, 8.5 Hz, 1H), 7.58 (d, J = 8.5 Hz, 1H); ^{13}C NMR (125 MHz, d^{6}-DMSO) δ ppm 190.23, 164.23, 136.97, 133.58, 132.61, 132.45, 130.97, 130.60; GCMS (EI) acquisition time, 19.82 min, m/z = 216.8 (1.81%), m/z = 104.9 (100%), m/z = 172.8 (100%). Compound **10b** (14.0 mg, 9% yield): ^{1}H NMR (500 MHz, d^{6}-DMSO) δ ppm 10.23 (s, 1H), 8.12 (brs, 1H), 7.87 (brs, 1H), 7.86 (s, 1H), 7.72 (s, 1H); GCMS (EI) acquisition time, 21.43 min, m/z = 216.8 (64.20%), m/z = 200.8 (100%).

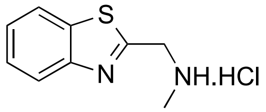

1-(Benzo[*d*]thiazol-2-yl)-*N*-methylmethanamine hydrochloride (11): To a solution of compound **6c** (0.042 g, 0.19 mmol) in 5 mL of ethanol was added 5 mL HCl (6M). The resulting mixture was heated to gentle reflux overnight. Then the mixture was diluted with water (20 mL). The pH value of the solution was adjusted to 10–11, extracted with ethyl acetate (20 mL × 3), dried over Na_2SO_4, and concentrated to give the crude product. The crude product was then dissolved into dichloromethane (10 mL), treated with 1 mL HCl (4M in dioxane). The resulting mixture was further stirred for 10 min, and the solvent was removed under vacuum to give the crude product, which was further dissolved into deionized water (10 mL), washed with ethyl acetate (20 mL × 3), and lyophilized to give the product as white solid (30 mg, 74%). ^{1}H NMR (300 MHz, CD_3OD) δ ppm 8.06 (d, J = 7.2 Hz, 1H), 8.03 (d, J = 7.2 Hz, 1H), 7.56 (t, J = 7.2 Hz, 1H), 7.49 (t, J = 7.2 Hz, 1H), 4.87 (s, 2H), 2.91 (s, 3H); ^{13}C NMR (75 MHz, CD_3OD) δ ppm 161.18, 152.62, 135.53, 126.67, 126.11, 123.07, 122.03, 49.06; HRMS (ESI) Calcd. for $C_9H_{11}ClN_2S$ $(M + H)^+$ 179.0643, found 179.0644.

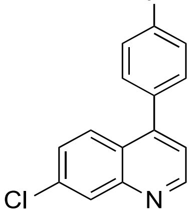

7-Chloro-4-(4-fluorophenyl)quinoline (12): It was prepared according to the reported procedure [2,3]. ^{1}H NMR (500 MHz, d^{6}-Acetone) δ ppm 8.98 (d, J = 4.0 Hz, 1H), 8.13 (d, J = 2.5

Hz, 1H), 7.92 (d, J = 9.0 Hz, 1H), 7.65 – 7.62 (m, 2H), 7.59 (dd, J = 2.0, 9.0 Hz, 1H), 7.47 (d, J = 4.5 Hz, 1H), 7.40 – 7.36 (m, 2H); ^{13}C NMR (75 MHz, d^6-DMSO) δ ppm 163.12 (d, J_{C-F} = 244.5 Hz), 152.13, 149.21, 147.23, 134.81, 133.80, 132.30 (d, J_{C-F} = 8.2 Hz), 128.77, 128.29, 128.15, 125.25, 122.68, 116.50 (d, J_{C-F} = 21.7 Hz); HRMS (ESI) Calcd. for $C_{15}H_9ClFN$ (M + H)$^+$ 258.0486, found 258.0486.

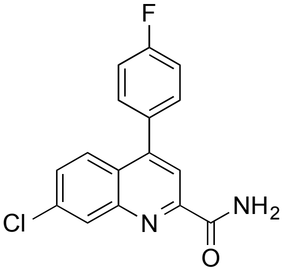

7-Chloro-4-(4-fluorophenyl)quinoline-2-carboxamide (13): According to the general procedure (method A), compound **12** (0.050 g, 0.19 mmol, 1.0 equiv), $(NH_4)_2S_2O_8$ (0.13 g, 0.58 mmol, 3.0 equiv), TsOH•H_2O (0.036 g, 0.19 mmol, 1.0 equiv), benzaldehyde (0.020 g, 0.19 mmol, 1.0 equiv), 2.5 mL formamide, and 2.5 mL water were used. After 24 h, the reaction mixture was subjected to the workup protocol outlined in the general procedure and purified through flash chromatography (hexanes:acetone = 10:1–8:1) to provide the title compound as white solid (50 mg, 88%). ^{1}H NMR (500 MHz, d^6-DMSO) δ ppm 8.33 (brs, 1H), 8.19 (s, 1H), 8.00 (s, 1H), 7.90 (brs, 1H), 7.89 (d, J = 9.0 Hz, 1H), 7.71 (dd, J = 2.5, 9.0 Hz, 1H), 7.65 – 7.62 (m, 2H), 7.42 (t, J = 9.0 Hz, 2H); ^{13}C NMR (125 MHz, d^6-DMSO) δ ppm 166.36, 163.24 (d, J_{C-F} = 245.0 Hz), 151.77, 149.01, 147.87, 135.63, 133.65 (d, J_{C-F} = 3.1 Hz), 132.35 (d, J_{C-F} = 8.5 Hz), 129.69, 129.02, 128.21, 126.11, 119.75, 116.60 (d, J_{C-F} = 21.5 Hz); HRMS (ESI) Calcd. for $C_{16}H_{10}ClFN_2O$ (M + Na)$^+$ 323.0363, found 323.0368.

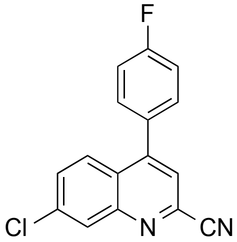

7-Chloro-4-(4-fluorophenyl)quinoline-2-carbonitrile (14): It was prepared according to the reported procedure.[4] To a solution of compound **13** (0.030 g, 0.10 mmol, 1 equiv) and triethylamine (0.031 g, 0.15 mmol, 1.5 equiv) in 3 mL of tetrahydrofuran was added the solution of trifluoroacetic anhydride (0.020 g, 0.20 mmol, 2 equiv) in 2 mL tetrahydrofuran at 0–5 °C. The resulting mixture was stirred for another 2 h at the same temperature. Then, the solution was diluted with ethyl acetate (30 mL), washed with brine (15 mL × 3), dried over Na_2SO_4, and concentrated to give the pure product as white solid (28 mg, 100%). ^{1}H NMR (300 MHz, d^6-DMSO) δ ppm 8.27 (d, J = 2.1 Hz, 1H), 8.04 (s, 1H), 7.92 (d, J = 9.0 Hz, 1H), 7.79 (dd, J = 2.1, 9.0 Hz, 1H), 7.68 – 7.63 (m, 2H), 7.44 (t, J = 8.7 Hz, 2H); ^{13}C NMR (75 MHz, d^6-DMSO) δ ppm 163.51 (d, J_{C-F} = 245.5 Hz),149.67, 148.95, 136.80, 134.57, 132.65 (d, J_{C-F} = 8.5 Hz), 132.33 (d, J_{C-F} = 3.2 Hz), 131.23, 128.95, 128.42, 126.12, 125.00, 117.94, 116.68 (d, J_{C-F} = 21.7 Hz);

HRMS (ESI) Calcd. for $C_{16}H_8ClFN_2$ $(M + H)^+$ 283.0438, found 283.0438.

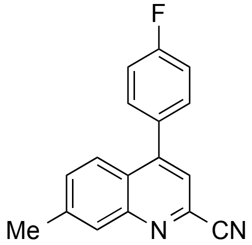

4-(4-Fluorophenyl)-7-methylquinoline-2-carbonitrile (15): It was prepared according to the reported procedure.[5] To a solution of **14** (0.030 g, 0.10 mmol, 1 equiv), potassium phosphate (0.042 g, 0.20 mmol, 2 equiv), 2-dicyclohexylphosphino-2',6'-dimethoxybiphenyl (0.010 g, 0.025 mmol, 0.25 equiv), methylboronic acid (0.018 mg, 0.30 mmol, 3 equiv) in 3 mL of toulene was added palladium acetate (2.00 mg, 0.010 mmol, 0.1 equiv). The resulting mixture was heated to gentle reflux for 24 h under argon. Then, the solution was cooled to room temperature and was diluted with ethyl acetate (30 mL), washed with brine (15 mL × 3), dried over Na_2SO_4, and concentrated to give the crude product. It was purified through flash chromatography (hexanes:ethyl acetate = 80:1–60:1) to provide the title compound as white solid (22 mg, 85%). ^{1}H NMR (500 MHz, d^6-acetone) δ ppm 7.96 (s, 1H), 7.88 (d, J = 8.5 Hz, 1H), 7.78 (s, 1H), 7.69–7.66 (m, 2H), 7.62 (d, J = 8.5 Hz, 1H), 7.40 (t, J = 9.0 Hz, 2H), 2.60 (s, 3H); ^{13}C NMR (125 MHz, d^6-acetone) δ ppm 163.49 (d, $J_{C\text{-}F}$ = 246.50 Hz),149.17, 149.06, 142.08, 133.52, 132.91 (d, $J_{C\text{-}F}$ = 3.4 Hz), 132.23, 132.00 (d, $J_{C\text{-}F}$ = 8.5 Hz), 129.12, 125.57, 125.46, 123.20, 117.82,116.80 (d, $J_{C\text{-}F}$ = 21.7 Hz), 20.95; HRMS (ESI) Calcd. for $C_{17}H_{11}FN_2$ $(M + H)^+$ 263.0979, found 263.0980.

Supplementary References:

1. (a) Martin, I.; Anvelt, J.; Vares, L.; Kühn, I.; Claesson, A. *Acta. Chem. Scand.* **1995**, *49*, 230–232; (b) Moinet, G.; Cravo, D. U.S. Patent 7,491,733, February 17, 2009.
2. Ducharme, Y.; Wu, T. Y.-H.; Frenette, R. PCT Int. Appl. WO 2007038865 A1, 2007.
3. Friesen, R. W.; Trimble, L. A. *Can. J. Chem.* **2004**, *82*, 206–214.
4. Wendt, M. D.; Rockway, T. W.; Geyer, A.; McClellan, W.; Weitzberg, M.; Zhao, X.; Mantei, R.; Nienaber, V. L.; Stewart, K.; Klinghofer, V.; Giranda, V. L. *J. Med. Chem.* **2004**, *47*, 303–324.
5. Minutolo, F.; Bellini, R.; Bertini, S.; Carboni, I.; Lapucci, A.; Pistolesi, L.; Prota, G.; Rapposelli, S.; Solati, F.; Tuccinardi, T.; Martinelli, A.; Stossi, F.; Carlson, K. E.; Katzenellenbogen, B. S.; Katzenellenbogen, J. A.; Macchia, M. *J. Med. Chem.* **2008**, *51*, 1344–1351.

Spectral Data for the Products

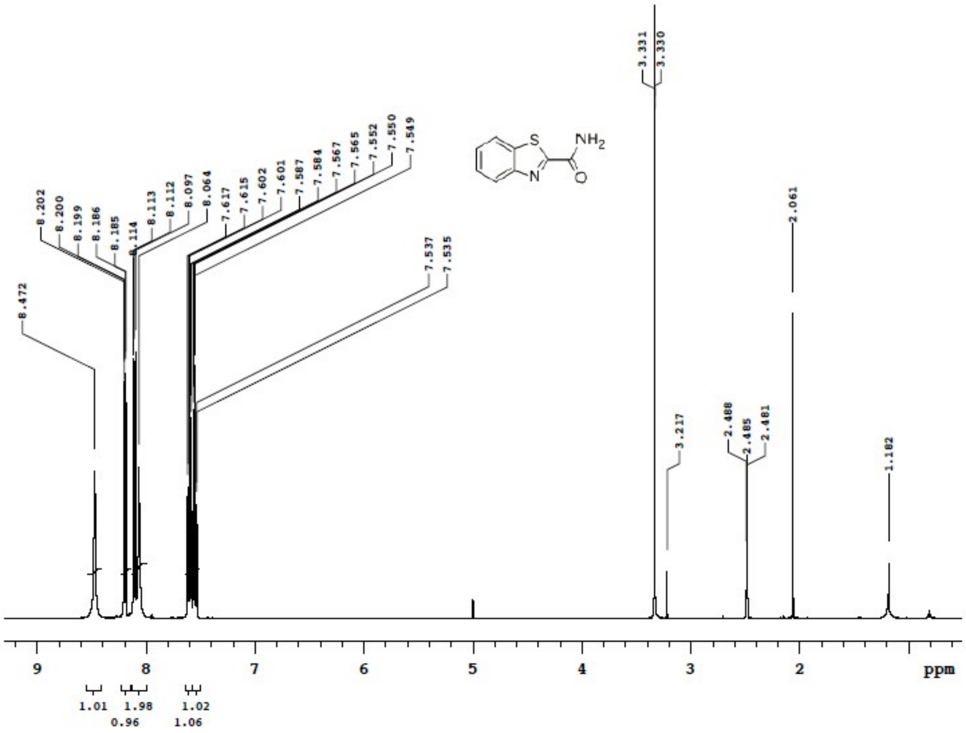

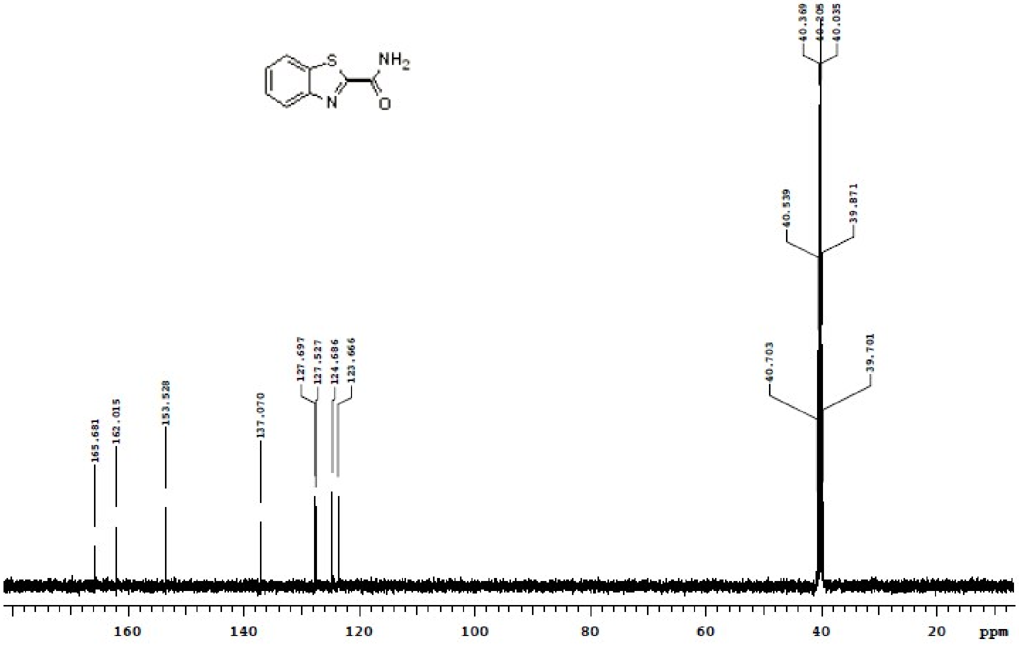

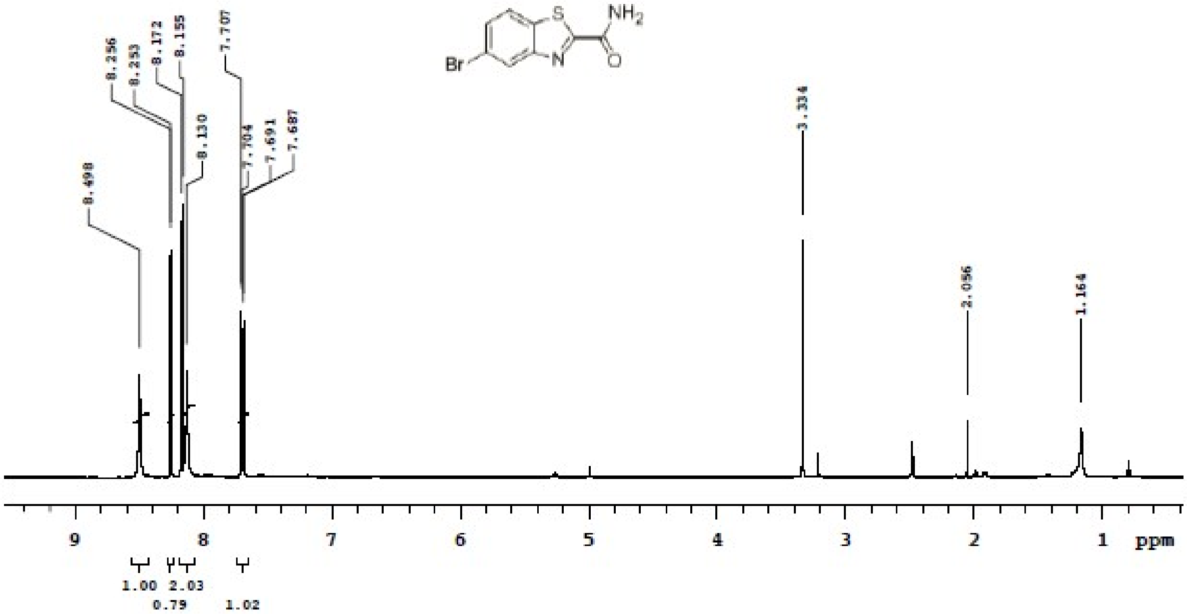

S
NH2
Br
N
O
8.498
8.256
8.253
8.172
8.155
8.130
7.707
7.704
7.691
7.687
3.334
2.056
1.164
9 8 7 6 5 4 3 2 1 ppm
1.00
0.79
2.03
1.02

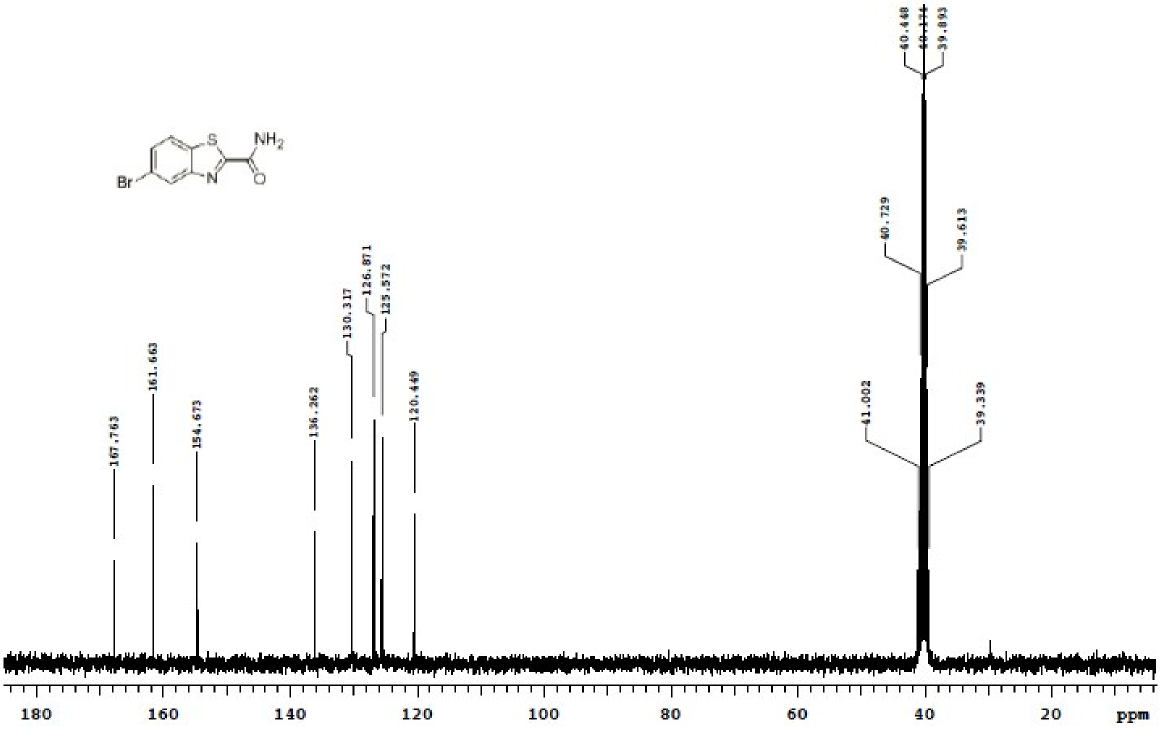

S
NH2
Br
N
O
167.763
161.663
154.673
136.262
130.317
126.871
125.572
120.449
41.002
40.729
40.448
39.893
39.613
39.339
180 160 140 120 100 80 60 40 20 ppm

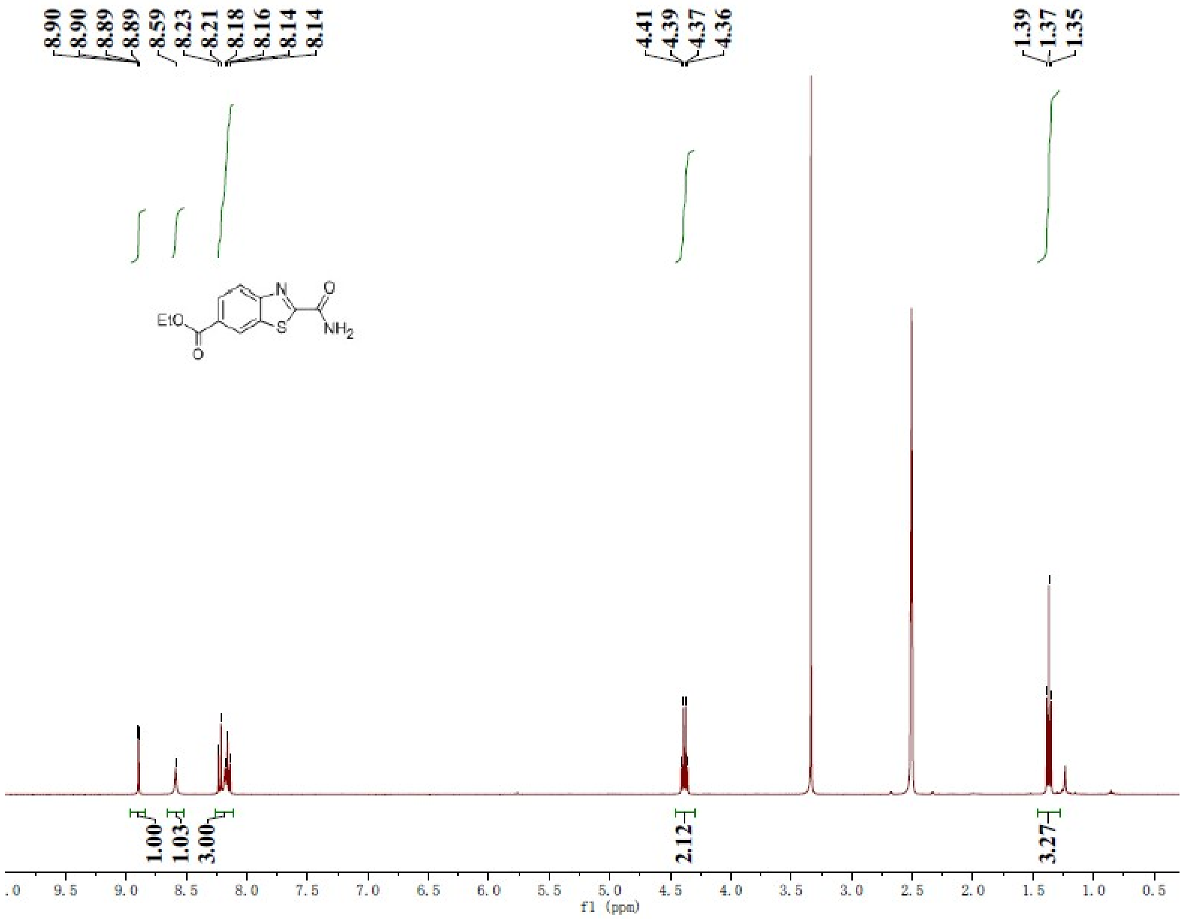
8.90
8.90
8.89
8.89
8.59
8.23
8.21
8.18
8.16
8.14
8.14
4.41
4.39
4.37
4.36
1.39
1.37
1.35
EtO
N
S
O
NH2
O
1.00
1.03
3.00
2.12
3.27
f1 (ppm)

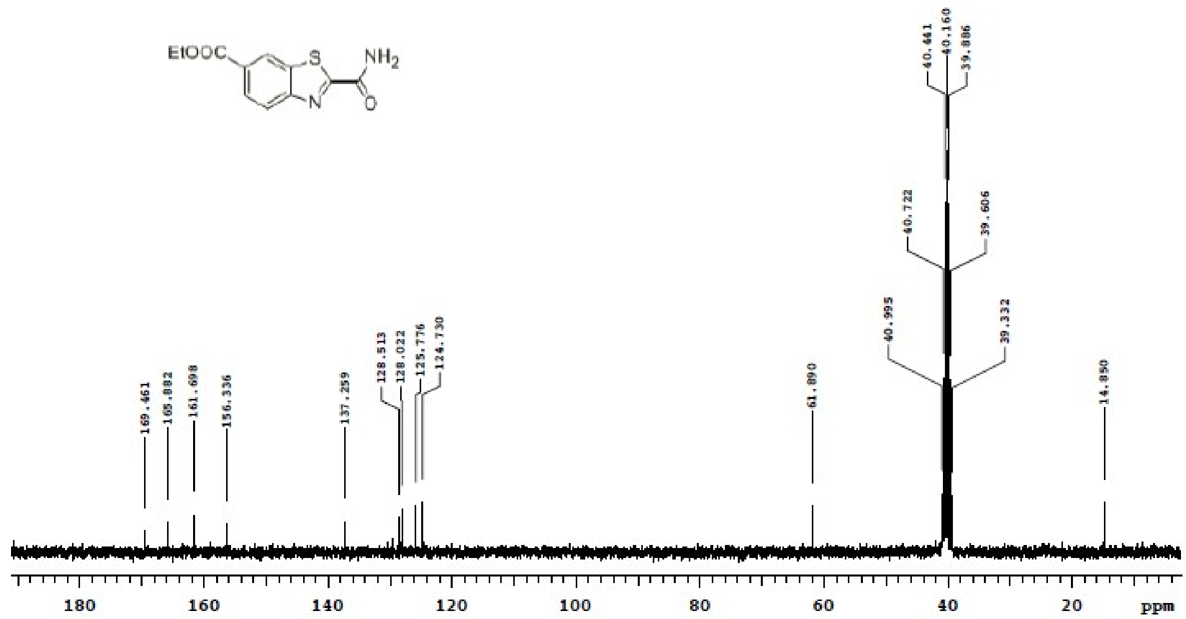
EtOOC
S
NH2
N
O
169.461
165.882
161.698
156.336
137.259
128.513
128.022
125.776
124.730
61.890
40.995
40.722
40.441
40.160
39.886
39.606
39.332
14.850
ppm

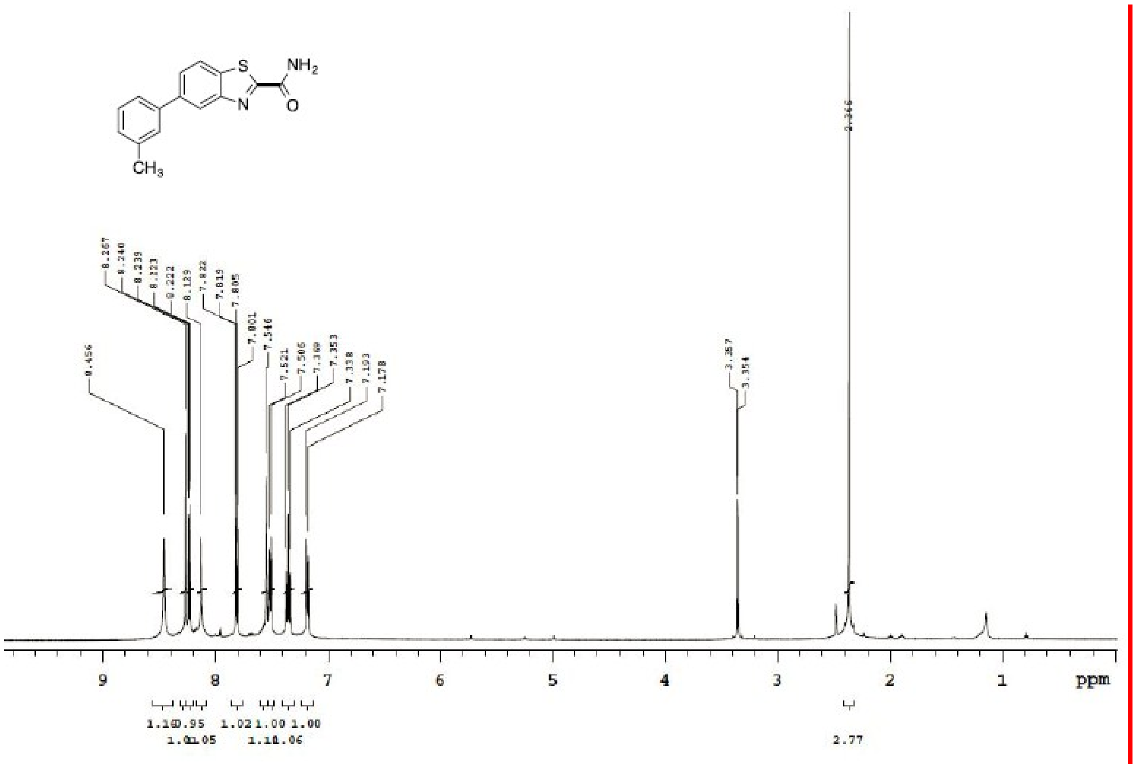

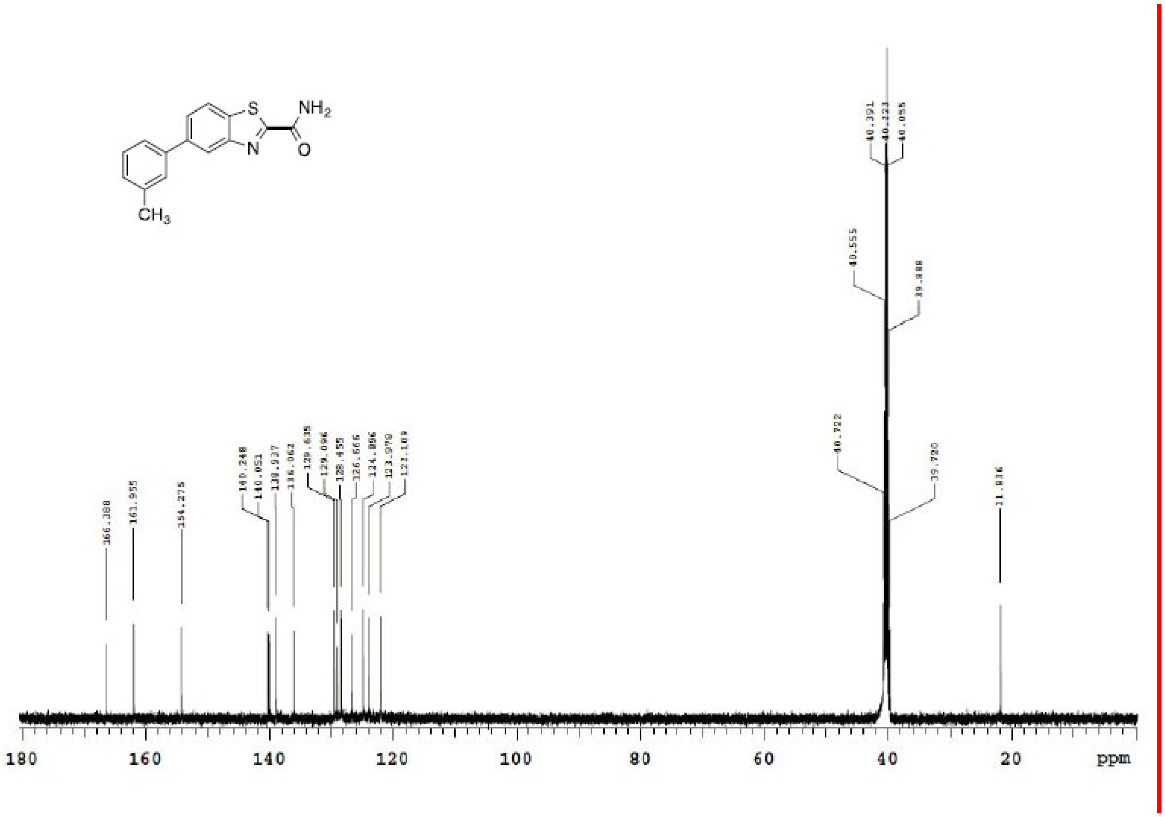

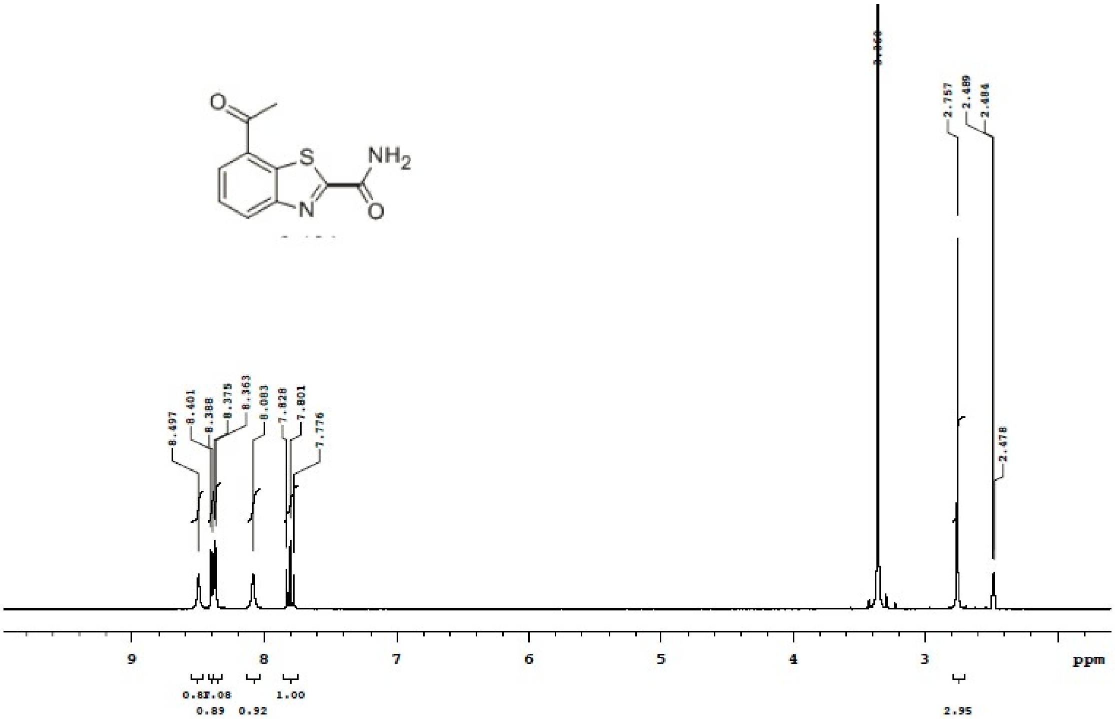
O
S
NH2
N
O
8.497
8.401
8.388
8.375
8.363
8.083
7.828
7.801
7.776
2.757
2.489
2.484
2.478
9
8
7
6
5
4
3
ppm
0.87
1.08
0.89
0.92
1.00
2.95

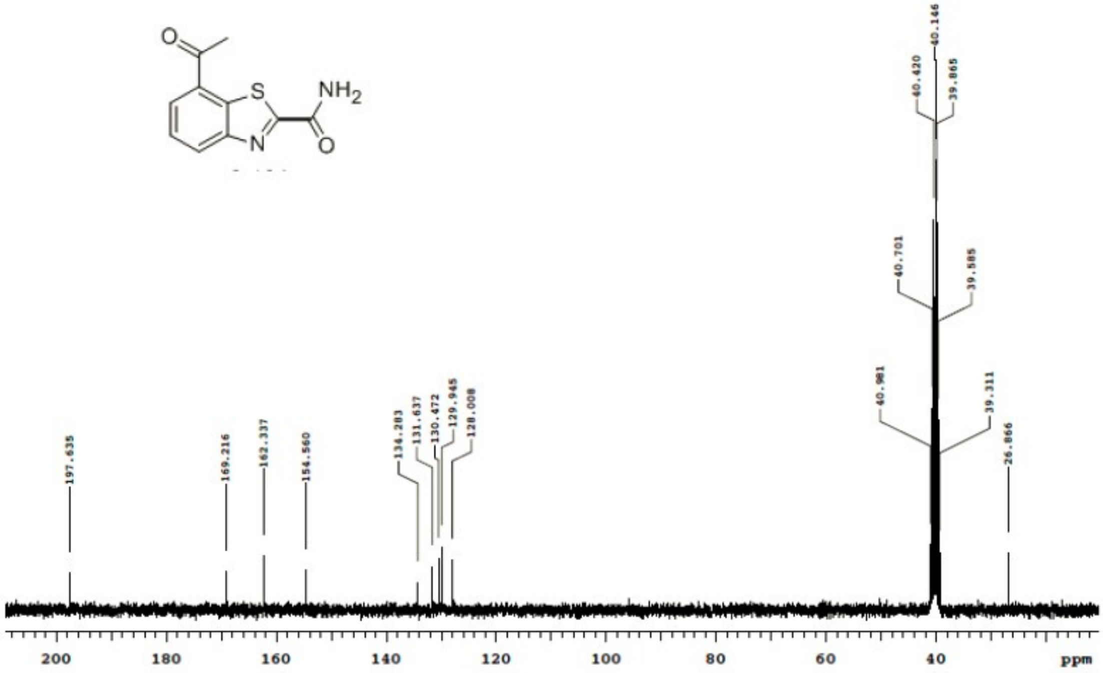
O
S
NH2
N
O
197.635
169.216
162.337
154.560
134.283
131.637
130.472
129.945
128.008
40.981
40.701
40.420
40.146
39.865
39.585
39.311
26.866
200
180
160
140
120
100
80
60
40
ppm

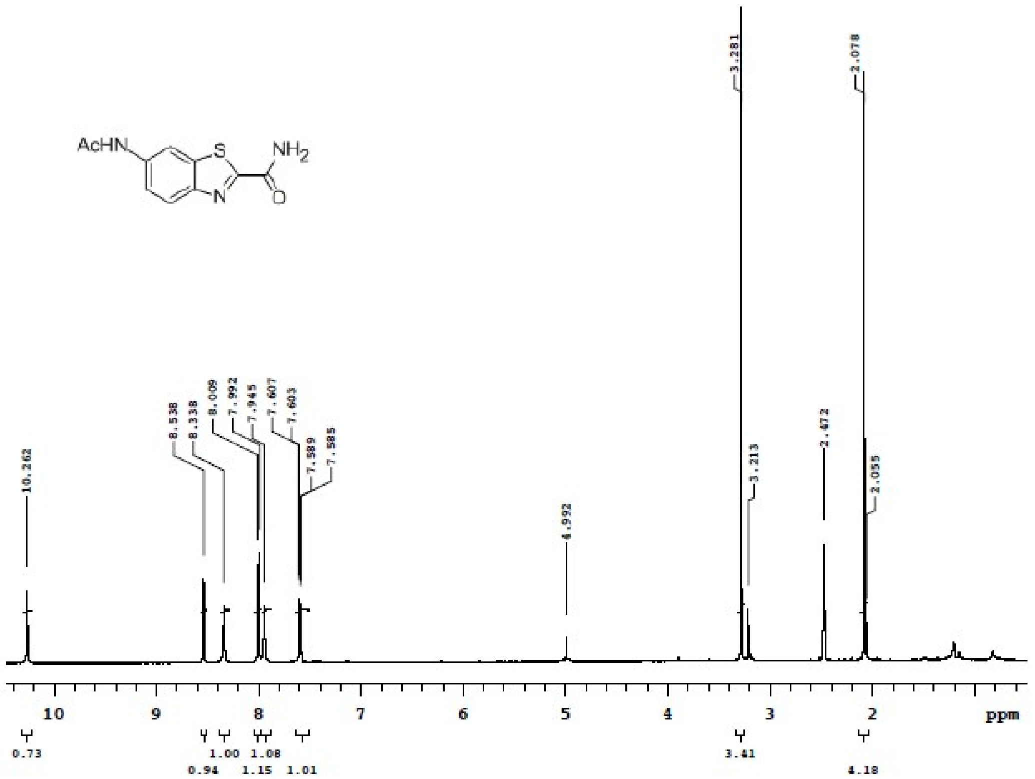

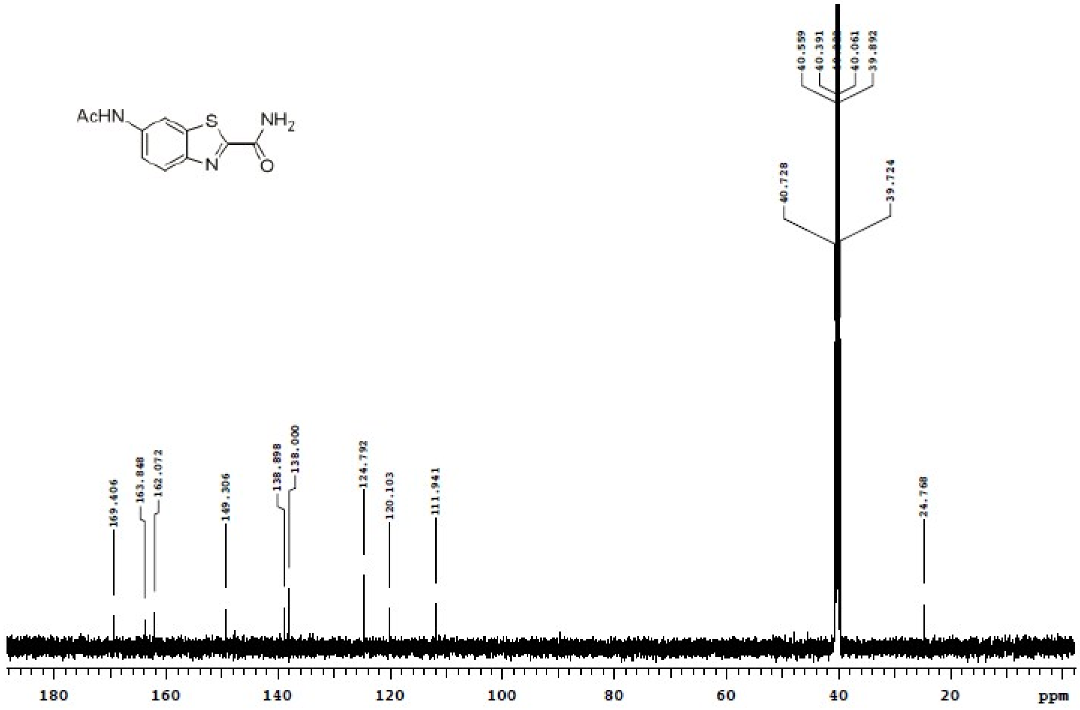

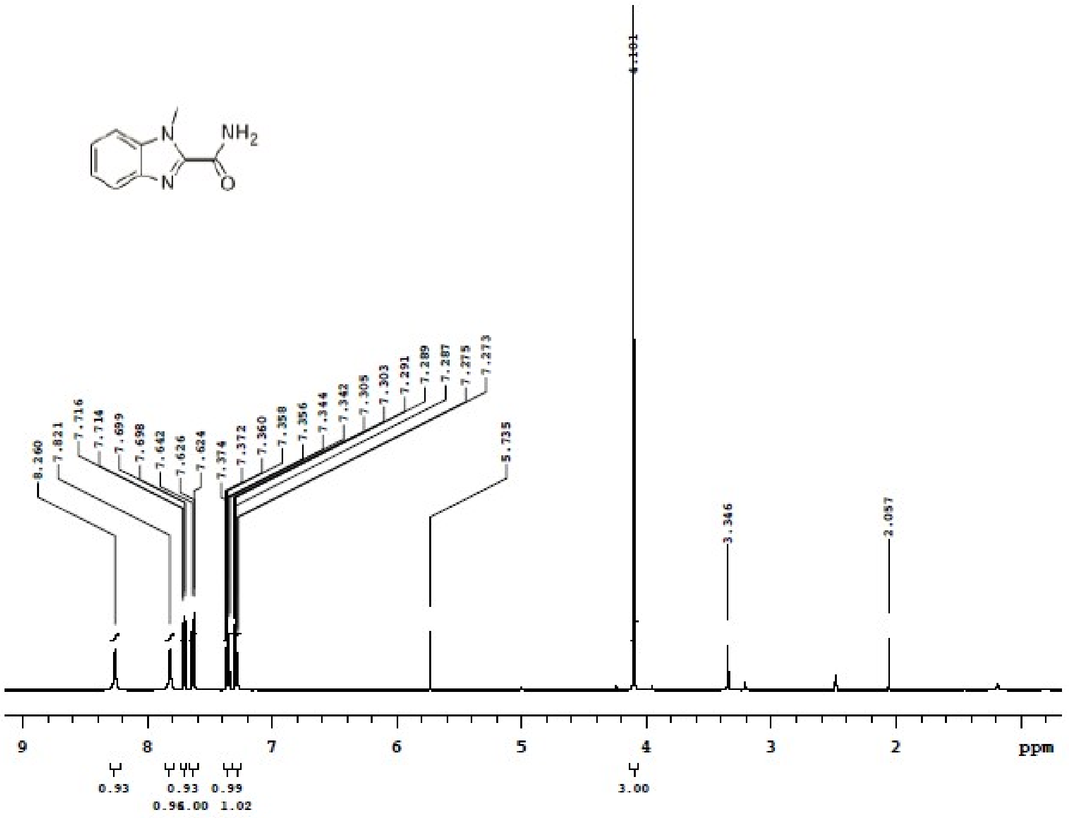
NH2
N
N
O
8.260
7.821
7.716
7.714
7.699
7.698
7.642
7.626
7.624
7.374
7.372
7.360
7.358
7.356
7.344
7.342
7.305
7.303
7.291
7.289
7.287
7.275
7.273
5.735
4.101
3.346
2.057
0.93
0.96
0.93
1.00
0.99
1.02
3.00
ppm

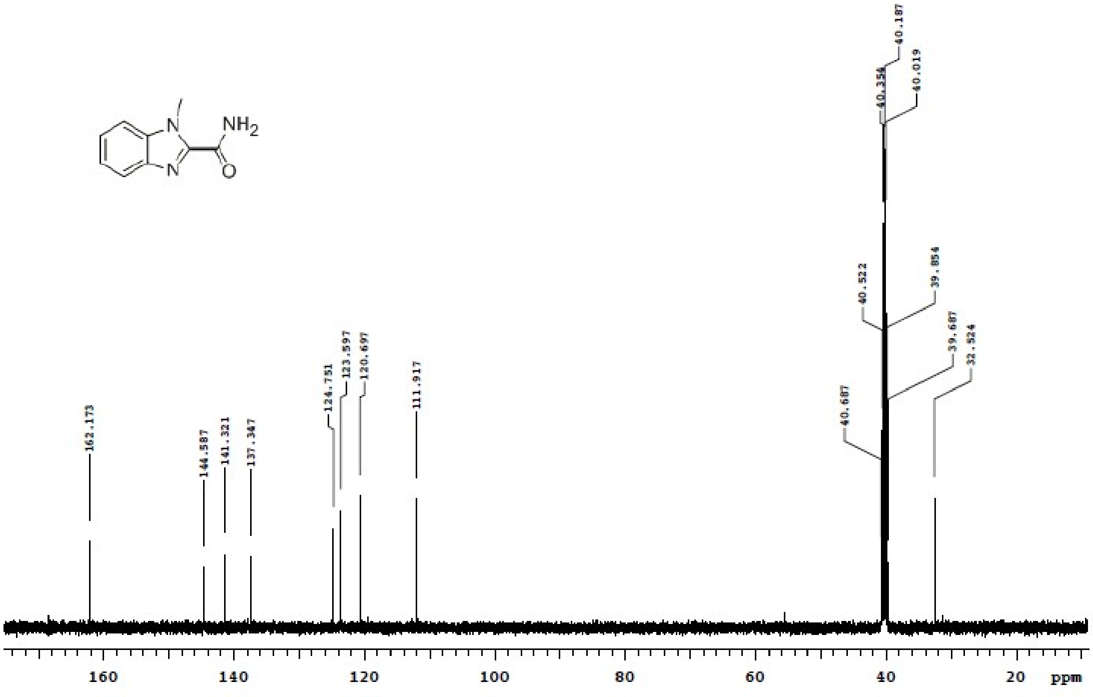
NH2
N
N
O
162.173
144.587
141.321
137.347
124.751
123.597
120.697
111.917
40.687
40.522
40.354
40.187
40.019
39.854
39.687
32.524
ppm

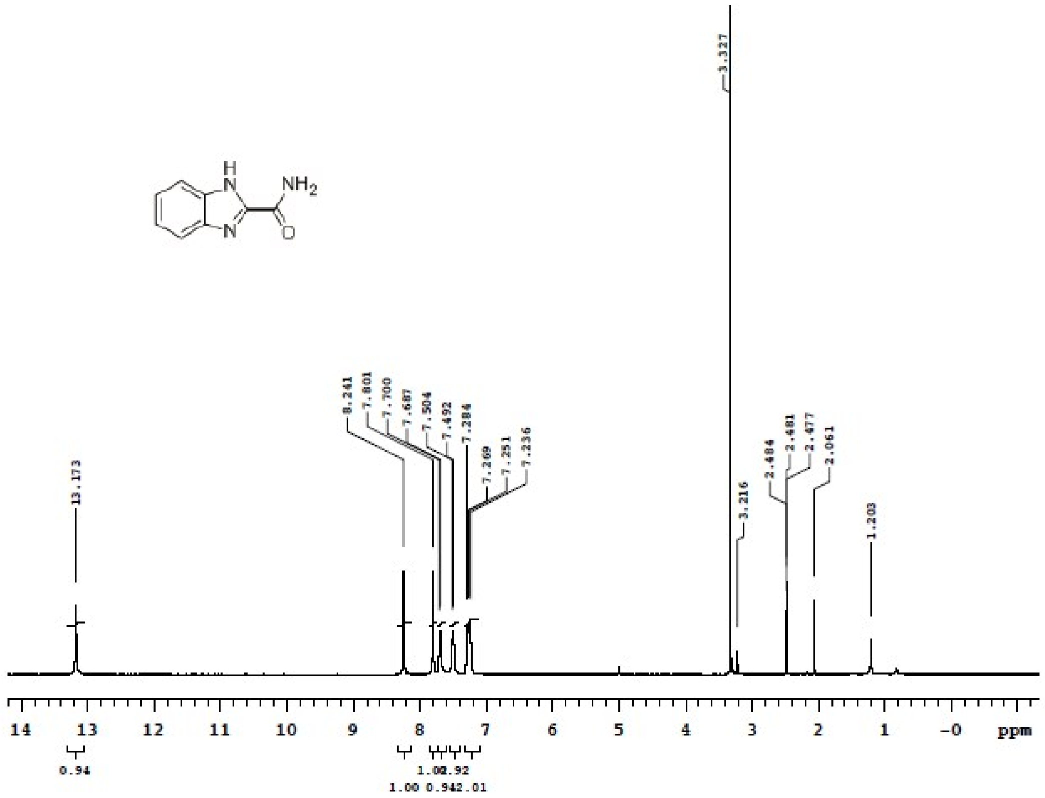
H
NH2
N
O
13.173
8.241
7.801
7.700
7.687
7.504
7.492
7.284
7.269
7.251
7.236
3.327
3.216
2.484
2.481
2.477
2.061
1.203
14 13 12 11 10 9 8 7 6 5 4 3 2 1 -0 ppm
0.94
1.00
1.02
2.92
0.94
2.01

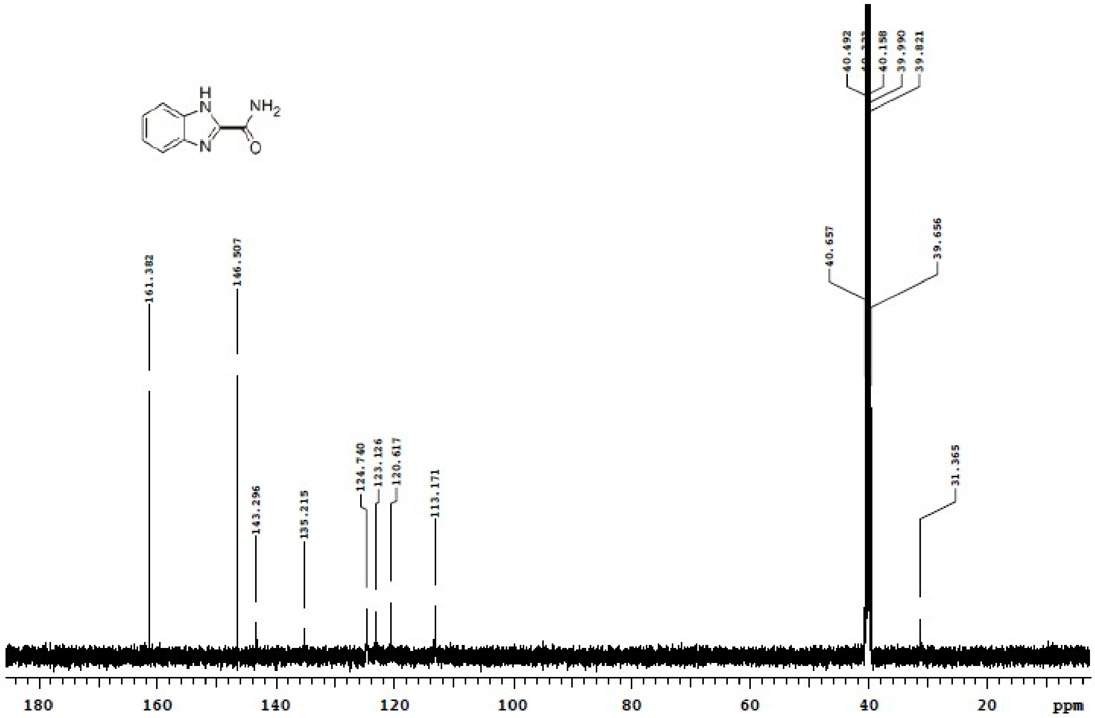
H
NH2
N
O
161.382
146.507
143.296
135.215
124.740
123.126
120.617
113.171
40.492
40.158
39.990
39.821
40.657
39.656
31.365
180 160 140 120 100 80 60 40 20 ppm

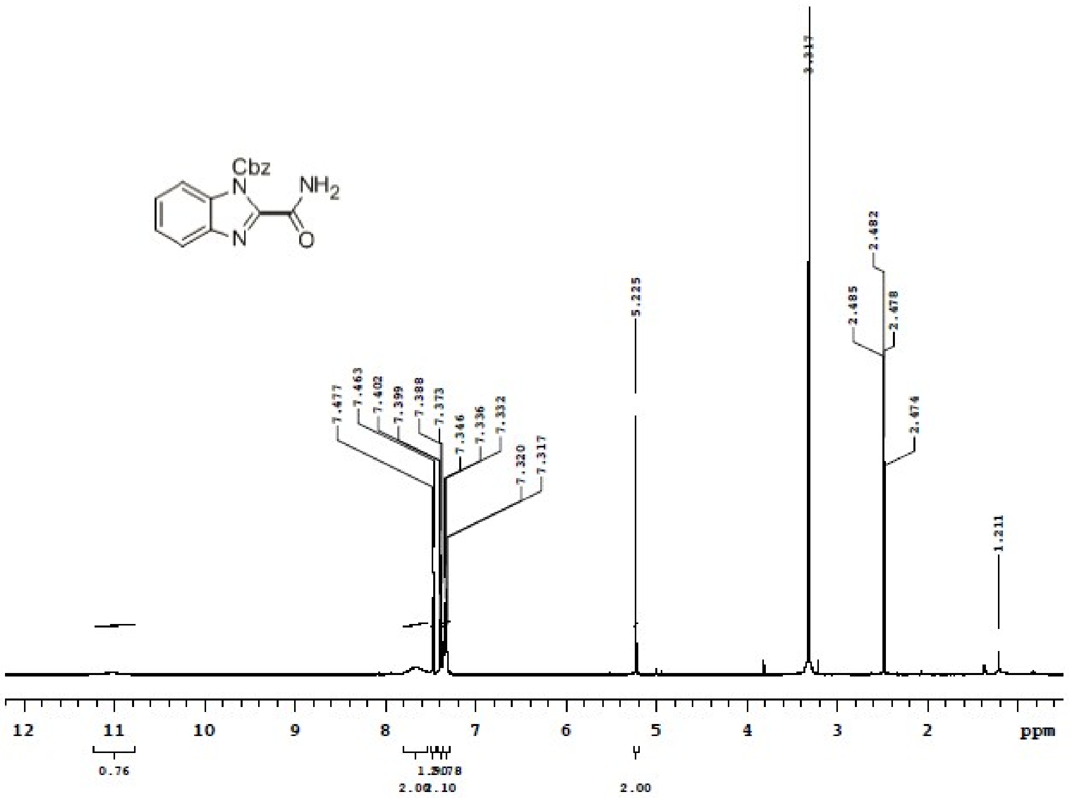
Cbz
NH2
N
O
7.477
7.463
7.402
7.399
7.388
7.373
7.346
7.336
7.332
7.320
7.317
5.225
2.485
2.482
2.478
2.474
1.211
12
11
10
9
8
7
6
5
4
3
2
ppm
0.76
2.00
2.00

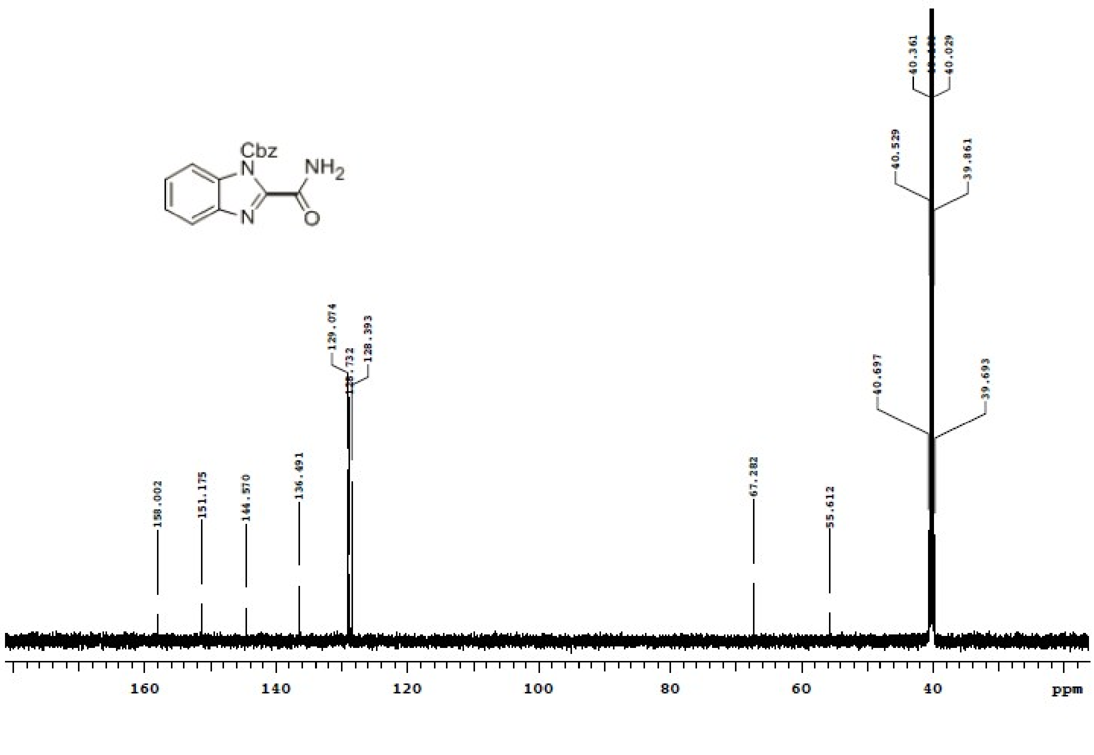
Cbz
NH2
N
O
40.361
40.029
40.529
39.861
129.074
128.393
40.697
39.693
158.002
151.175
144.570
136.491
67.282
55.612
160
140
120
100
80
60
40
ppm

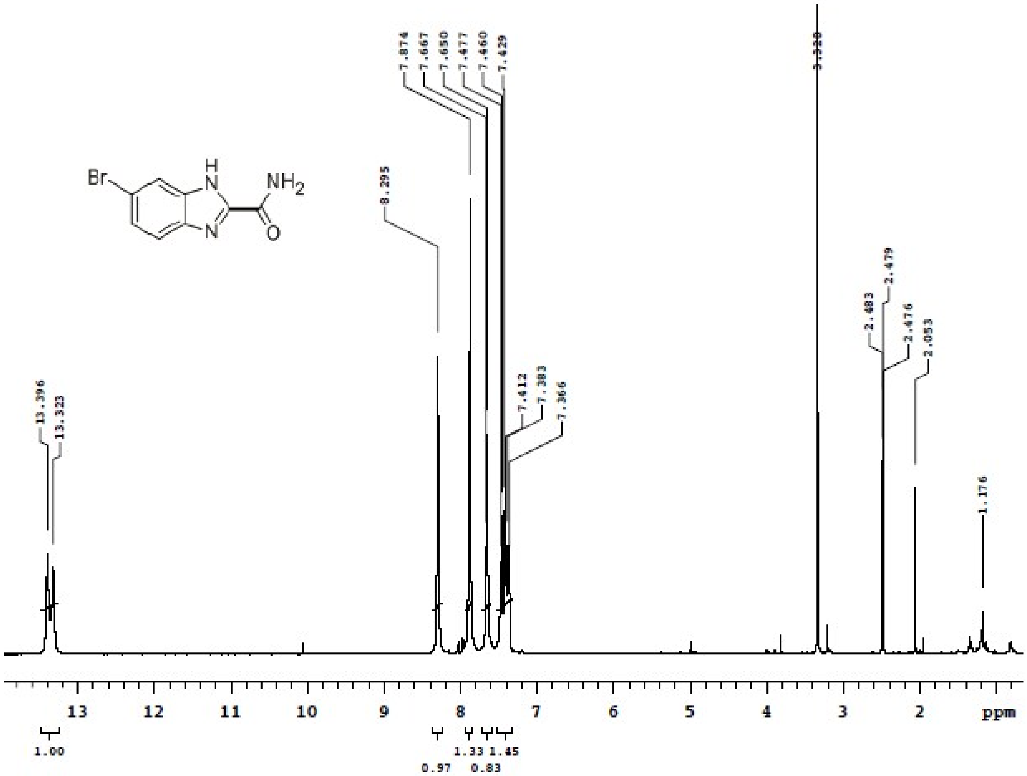

Br
H
N
NH2
N
O
13.396
13.323
8.295
7.874
7.667
7.650
7.477
7.460
7.429
7.412
7.383
7.366
3.328
2.483
2.479
2.476
2.053
1.176
1.00
0.97
1.33
0.83
1.45
ppm

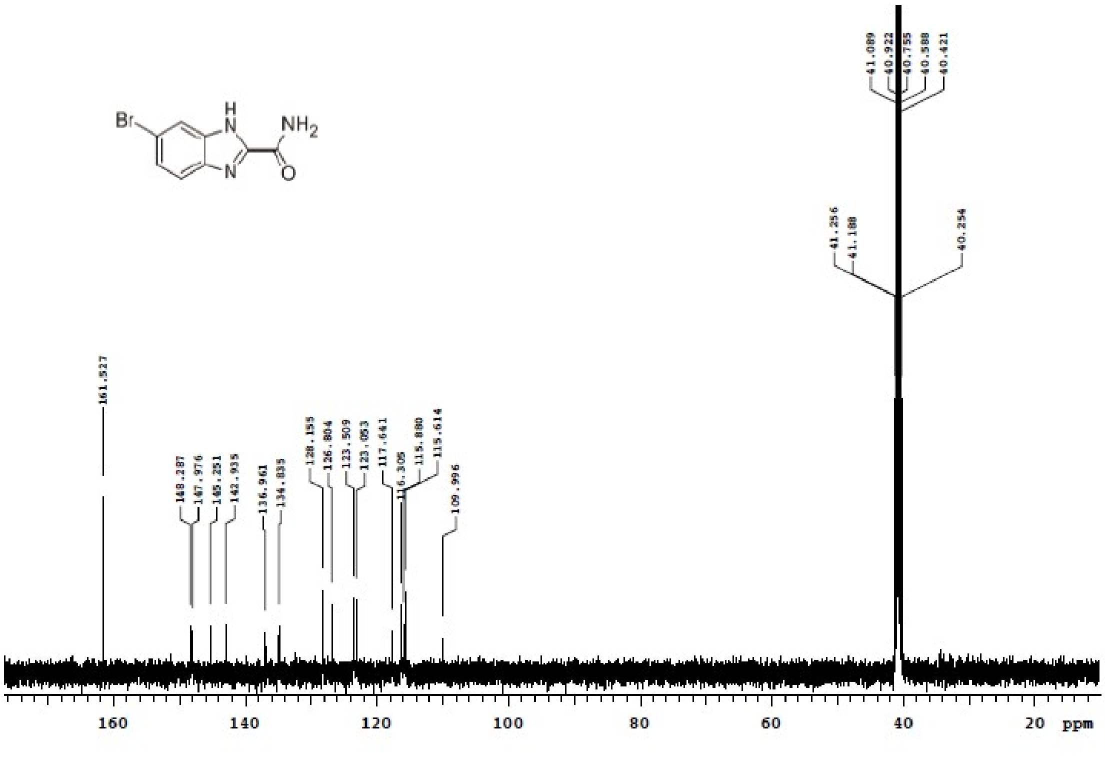

Br
H
N
NH2
N
O
161.527
148.287
147.976
145.251
142.935
136.961
134.835
128.155
126.804
123.509
123.053
117.641
116.305
115.880
115.614
109.996
41.256
41.188
41.089
40.922
40.755
40.588
40.421
40.254
ppm

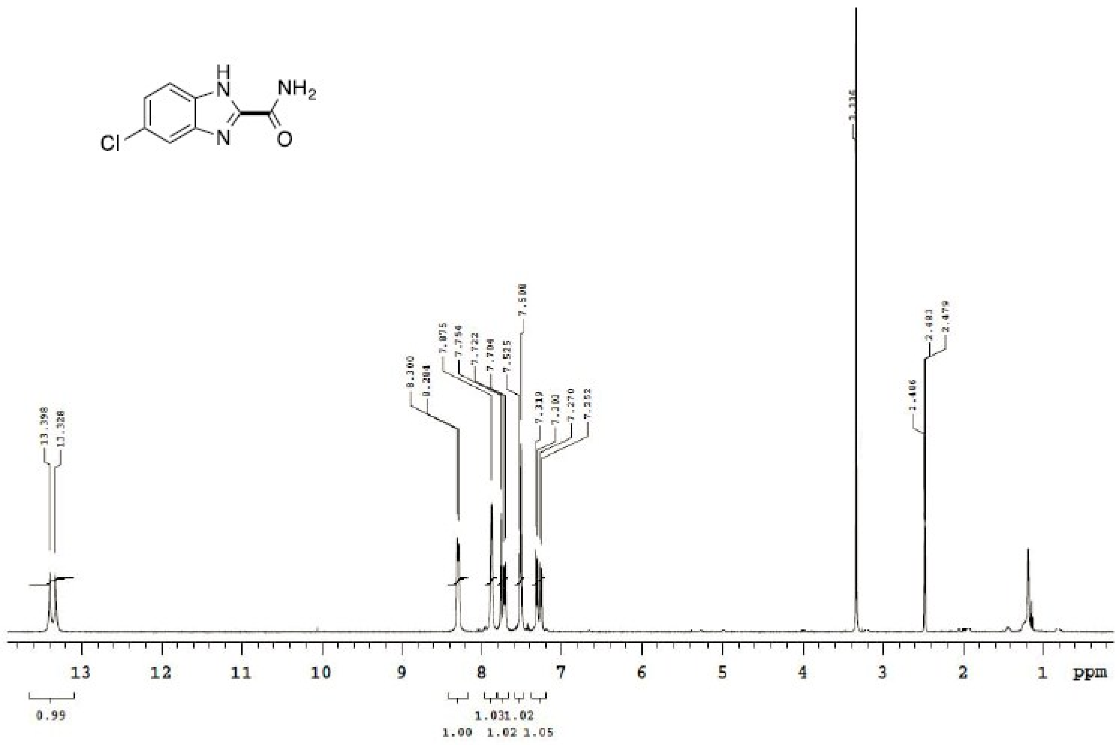

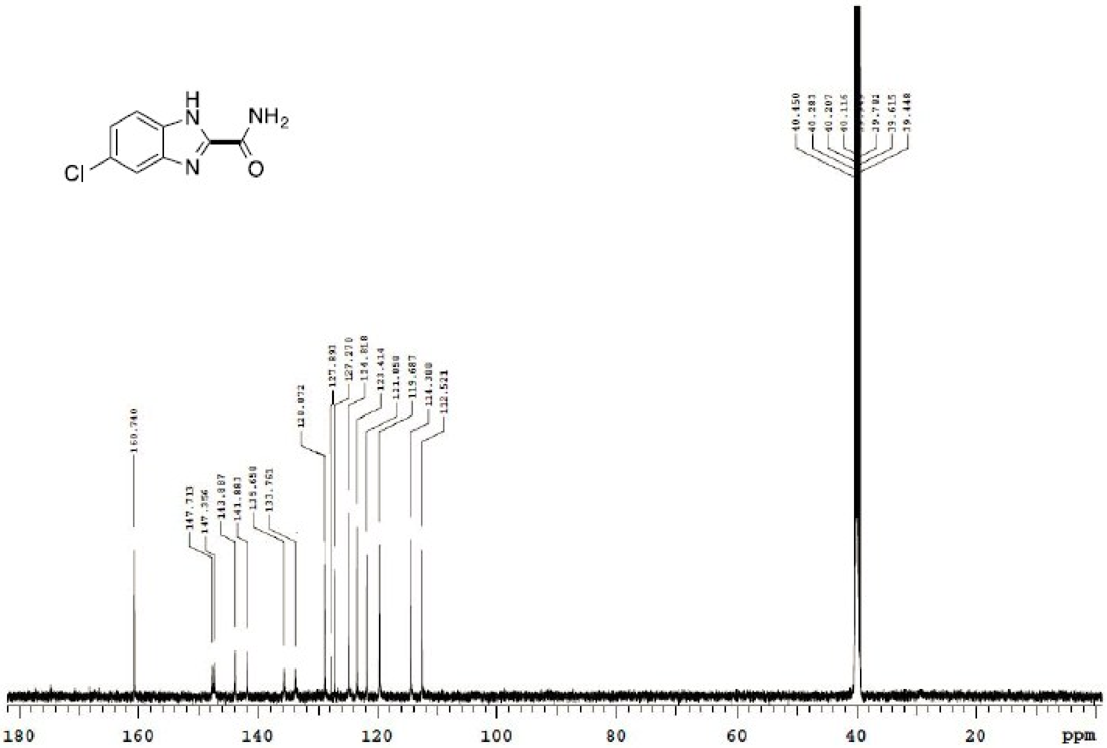

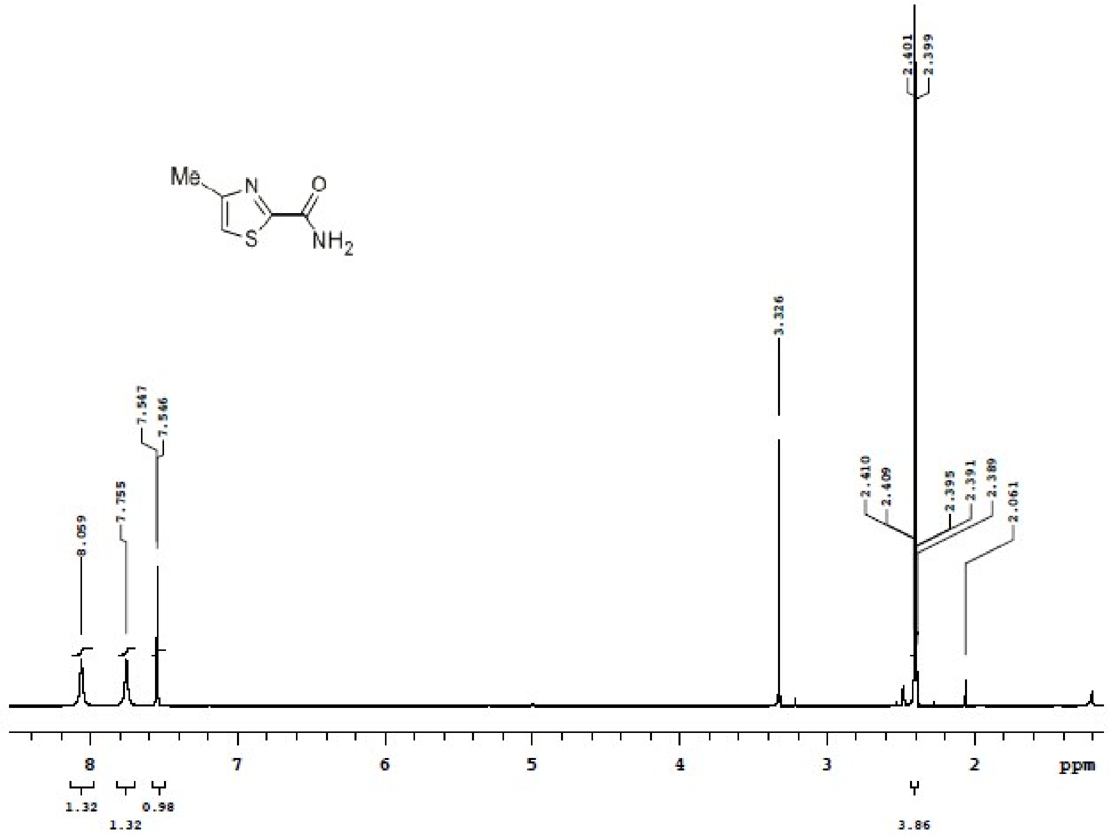
Me
N
O
S
NH2
8.059
7.755
7.547
7.546
3.326
2.401
2.399
2.410
2.409
2.395
2.391
2.389
2.061
8
7
6
5
4
3
2
ppm
1.32
1.32
0.98
3.86

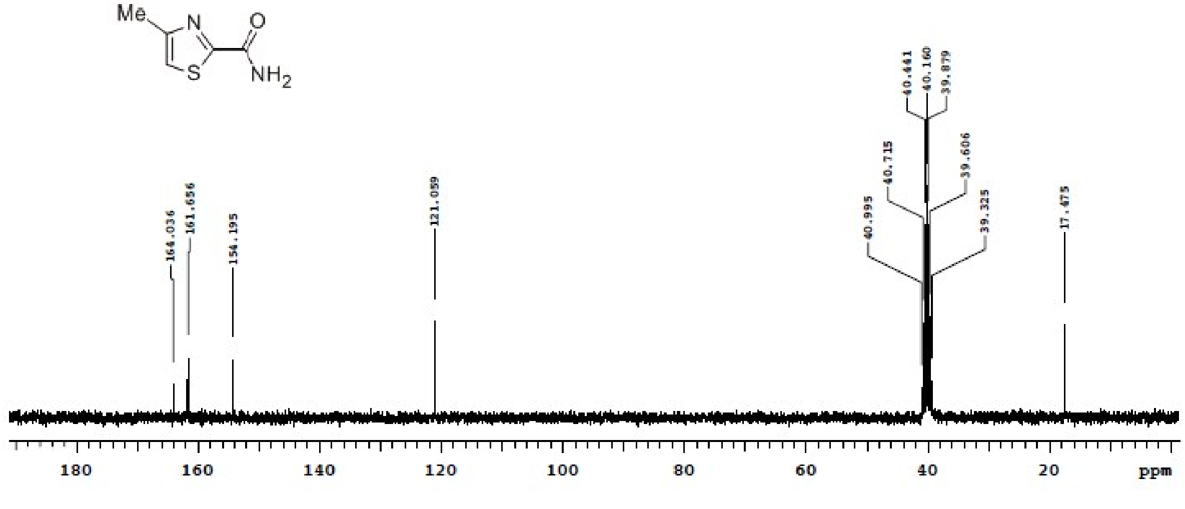
Me
N
O
S
NH2
164.036
161.656
154.195
121.059
40.441
40.160
39.879
40.715
39.606
40.995
39.325
17.475
180
160
140
120
100
80
60
40
20
ppm

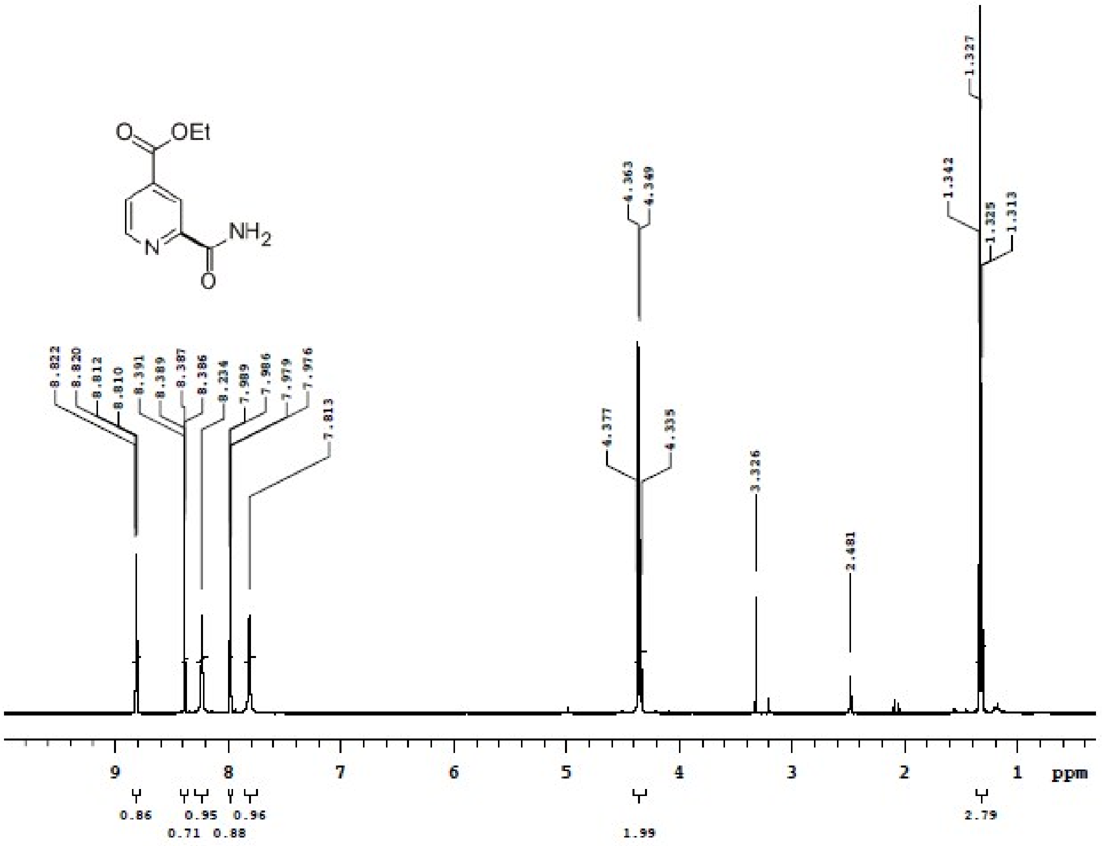

OEt
NH2
N
O
8.822
8.820
8.812
8.810
8.391
8.389
8.387
8.386
8.234
7.989
7.986
7.979
7.976
7.813
4.363
4.349
4.377
4.335
3.326
2.481
1.327
1.342
1.325
1.313
0.86
0.95
0.96
0.71
0.88
1.99
2.79
ppm

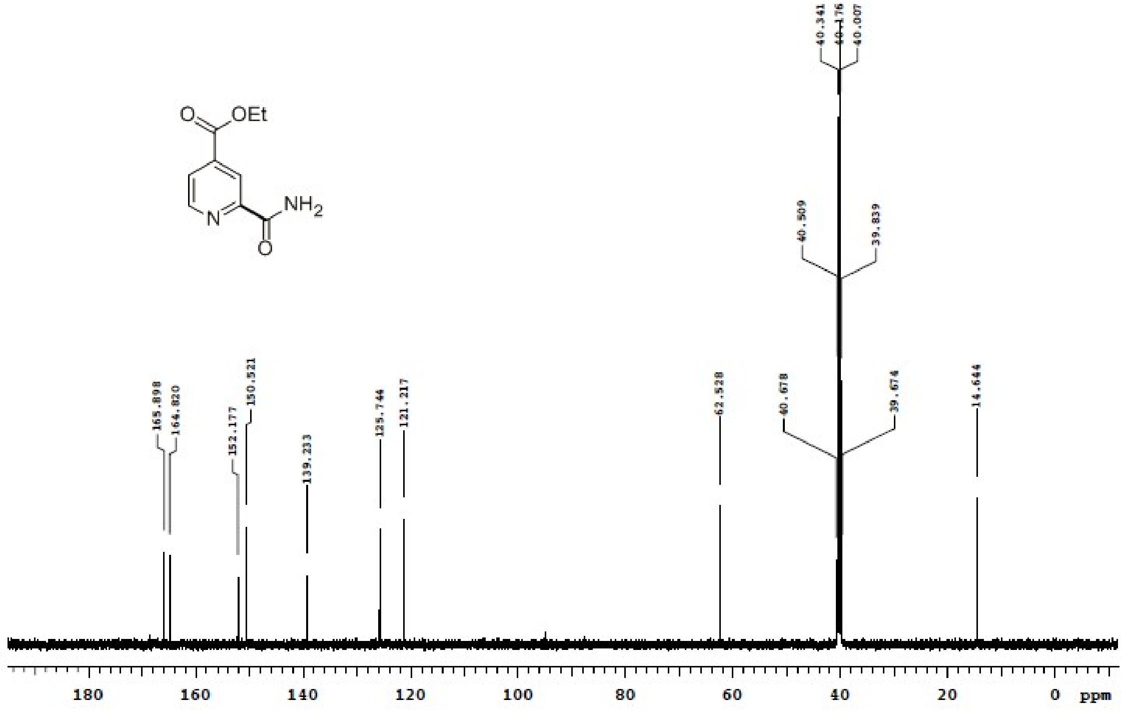

OEt
NH2
N
O
165.898
164.820
152.177
150.521
139.233
125.744
121.217
62.528
40.341
40.176
40.007
40.509
39.839
40.678
39.674
14.644
ppm

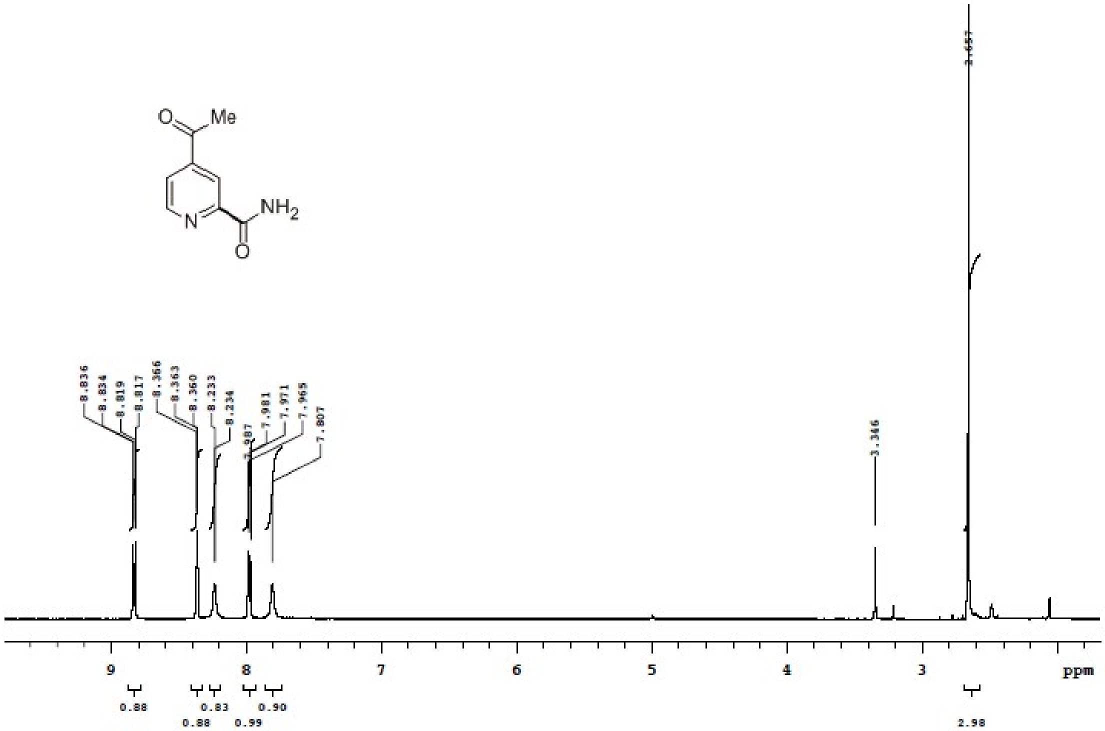

Me
NH2
N
O
8.836
8.834
8.819
8.817
8.366
8.363
8.360
8.233
8.234
7.987
7.981
7.971
7.965
7.807
3.346
9
8
7
6
5
4
3
ppm
0.88
0.88
0.83
0.99
0.90
2.98

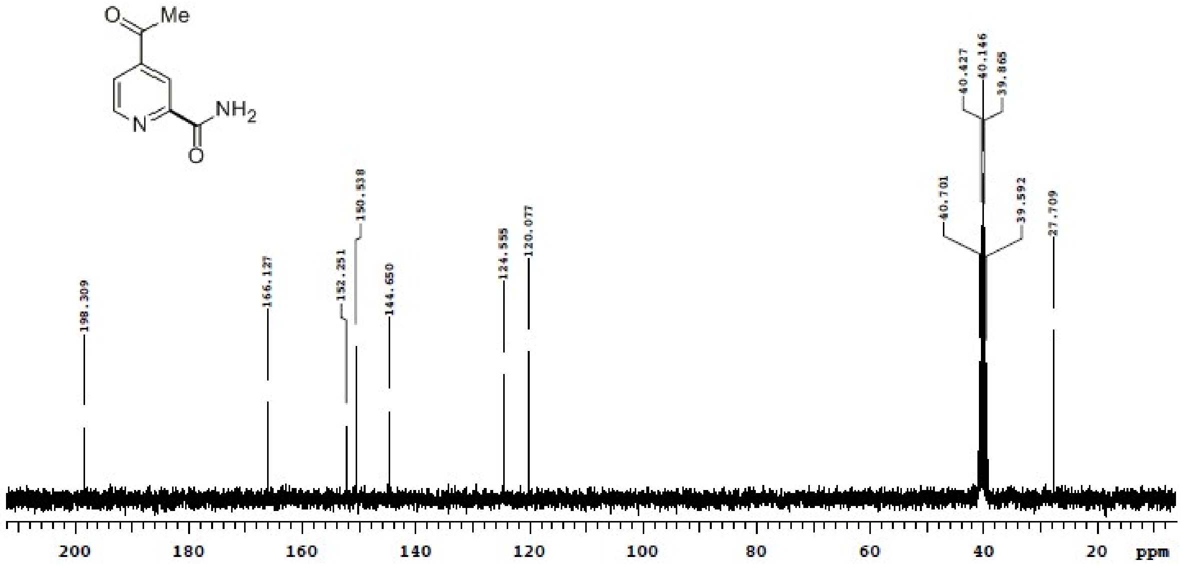

Me
NH2
N
O
198.309
166.127
152.251
150.538
144.650
124.555
120.077
40.701
40.427
40.146
39.865
39.592
27.709
200
180
160
140
120
100
80
60
40
20
ppm

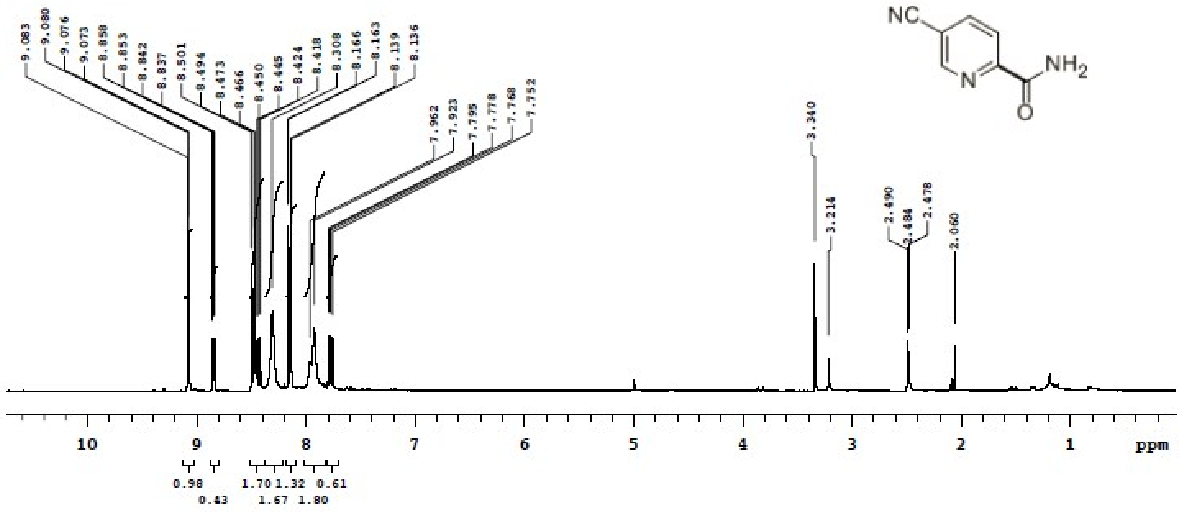
9.083
9.080
9.076
9.073
8.858
8.853
8.842
8.837
8.501
8.494
8.473
8.466
8.450
8.445
8.424
8.418
8.308
8.166
8.163
8.139
8.136
7.962
7.923
7.795
7.778
7.768
7.752
3.340
3.214
2.490
2.484
2.478
2.060
NC
N
NH2
O
10
9
8
7
6
5
4
3
2
1
ppm
0.98
0.43
1.70
1.67
1.32
1.80
0.61

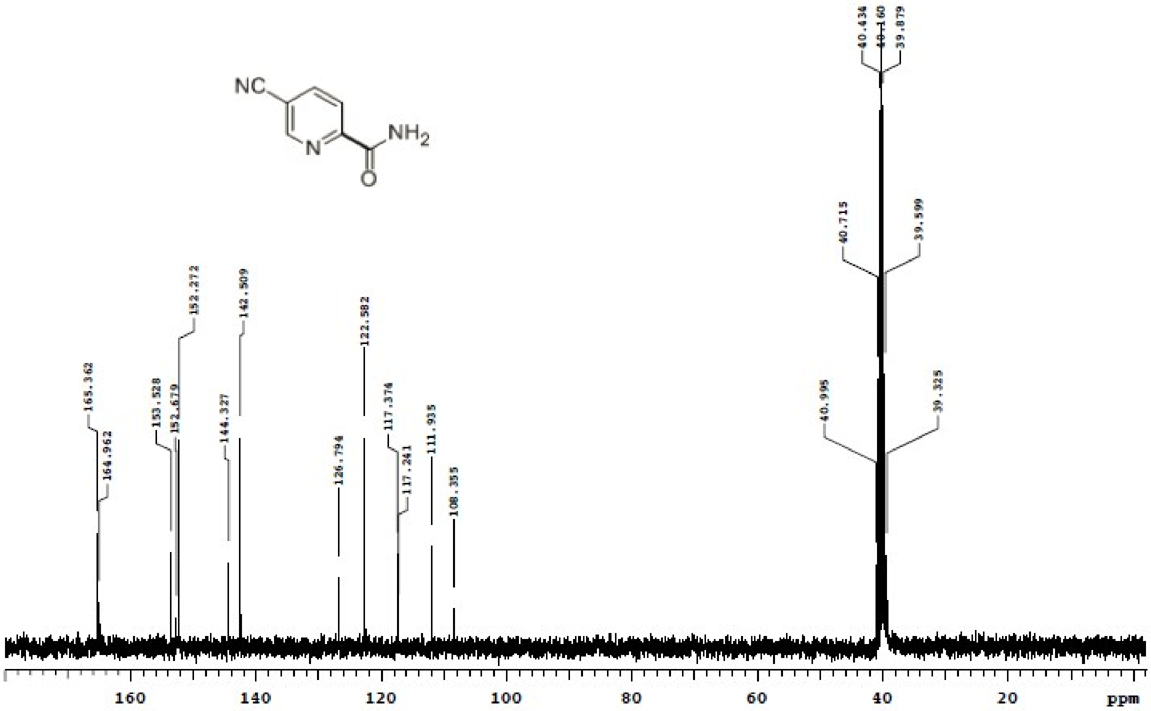
40.434
40.160
39.879
NC
N
NH2
O
40.715
39.599
153.272
142.509
122.582
165.362
153.528
152.679
144.327
40.995
39.325
164.962
117.374
111.935
126.794
117.241
108.355
160
140
120
100
80
60
40
20
ppm

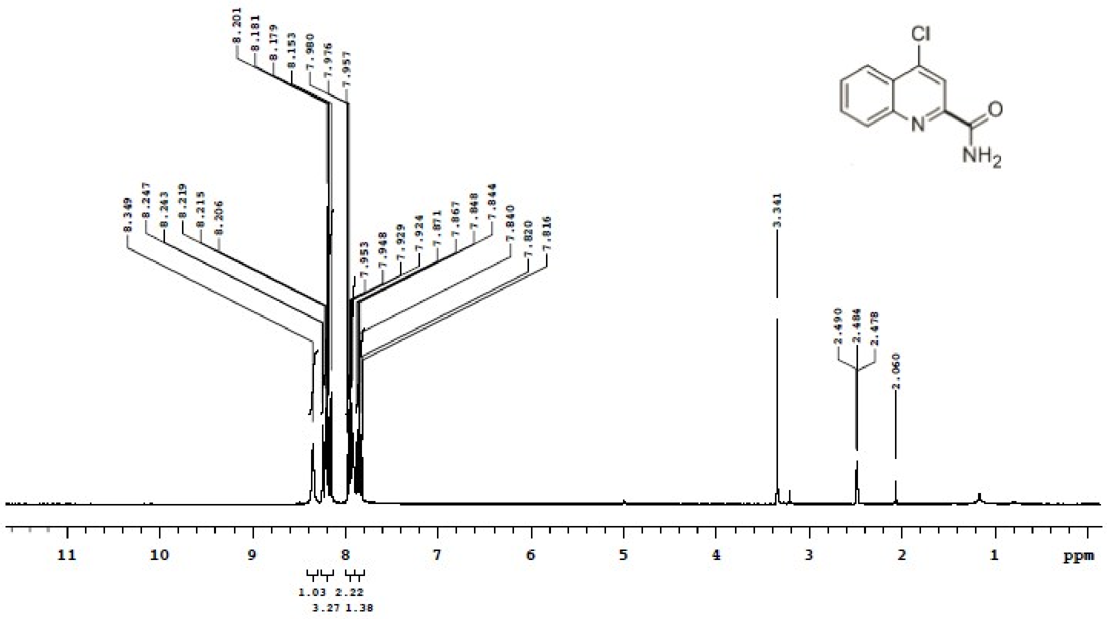
8.201
8.181
8.179
8.153
7.980
7.976
7.957
8.349
8.247
8.243
8.219
8.215
8.206
7.953
7.948
7.929
7.924
7.871
7.867
7.848
7.844
7.840
7.820
7.816
3.341
2.490
2.484
2.478
2.060
Cl
N
O
NH2
11
10
9
8
7
6
5
4
3
2
1
ppm
1.03
2.22
3.27
1.38

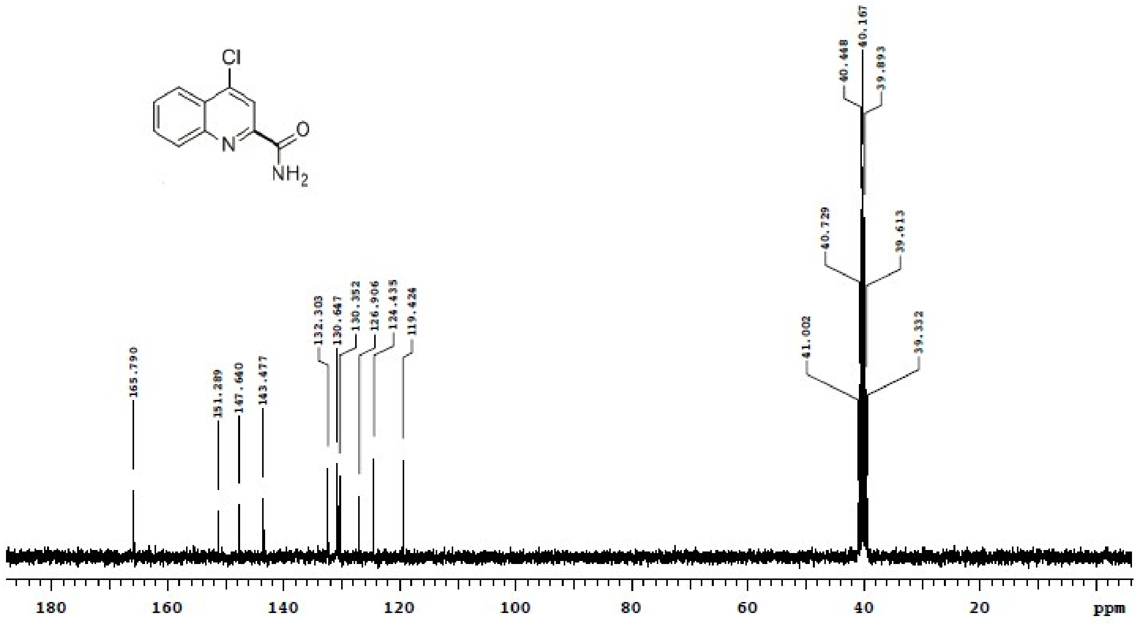
Cl
N
O
NH2
40.448
40.167
39.893
40.729
39.613
41.002
39.332
165.790
151.289
147.640
143.477
132.303
130.647
130.352
126.906
124.435
119.424
180
160
140
120
100
80
60
40
20
ppm

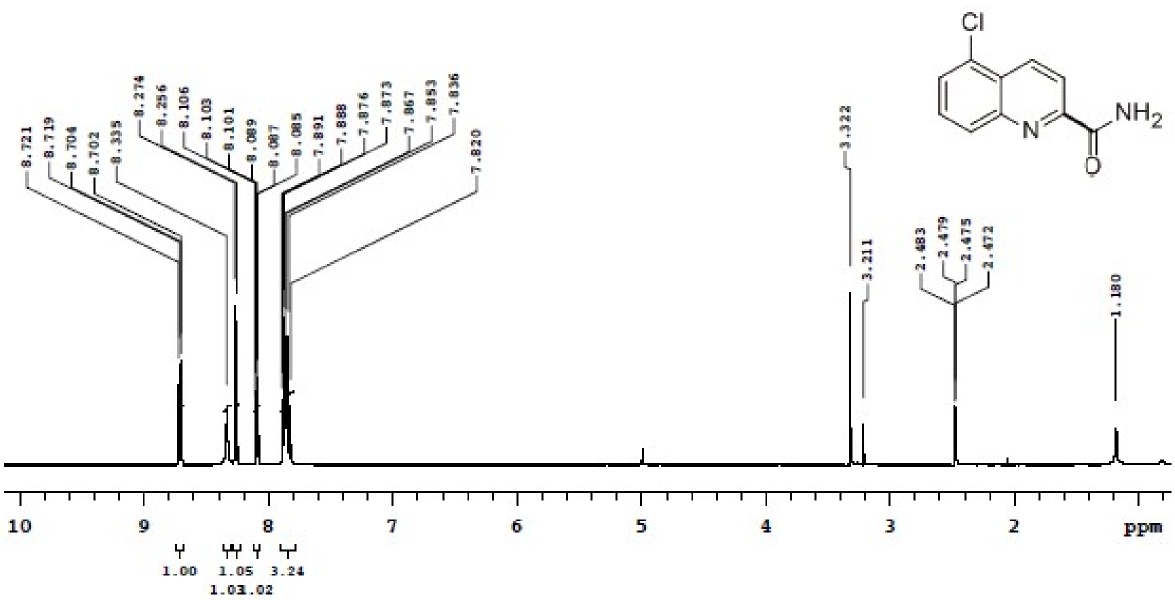
Cl
N
NH2
O
8.721
8.719
8.704
8.702
8.335
8.274
8.256
8.106
8.103
8.101
8.089
8.087
8.085
7.891
7.888
7.876
7.873
7.867
7.853
7.836
7.820
3.322
3.211
2.483
2.479
2.475
2.472
1.180
10
9
8
7
6
5
4
3
2
ppm
1.00
1.05
3.24
1.03
1.02

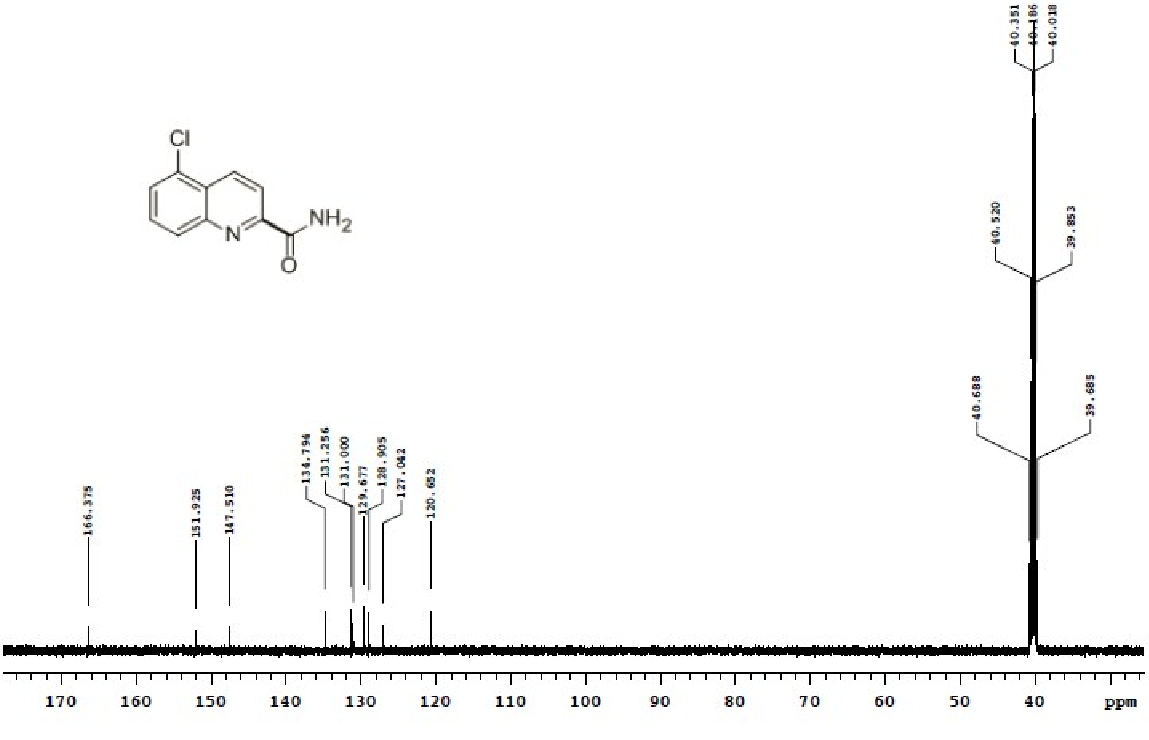
Cl
N
NH2
O
166.375
151.925
147.510
134.794
131.256
131.000
129.677
128.905
127.042
120.652
40.351
40.186
40.018
40.520
39.853
40.688
39.685
170
160
150
140
130
120
110
100
90
80
70
60
50
40
ppm

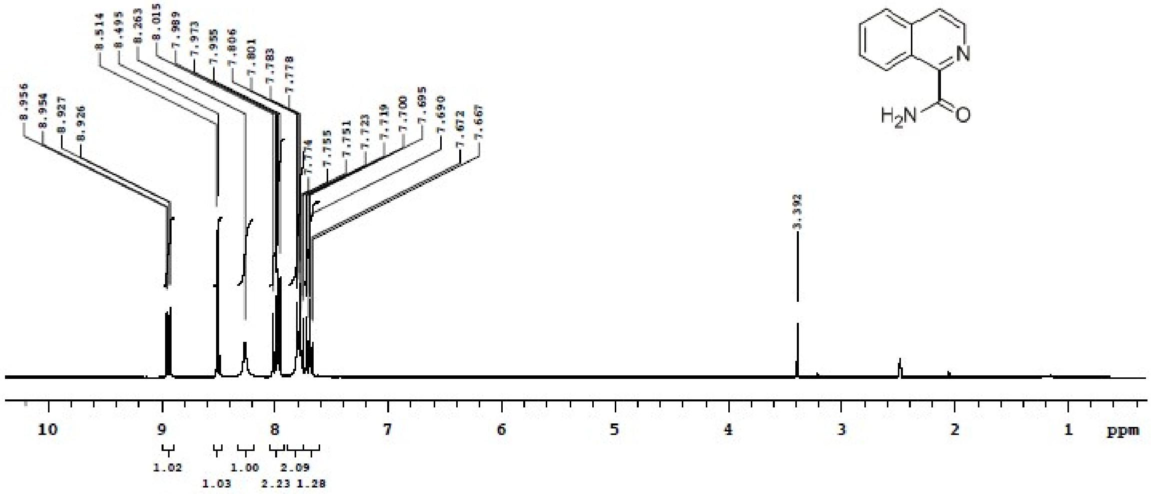
8.956
8.954
8.927
8.926
8.514
8.495
8.263
8.015
7.989
7.973
7.955
7.806
7.801
7.783
7.778
7.774
7.755
7.751
7.723
7.719
7.700
7.695
7.690
7.672
7.667
3.392
H_2N
O
N
10
9
8
7
6
5
4
3
2
1
ppm
1.02
1.03
1.00
2.23
2.09
1.28

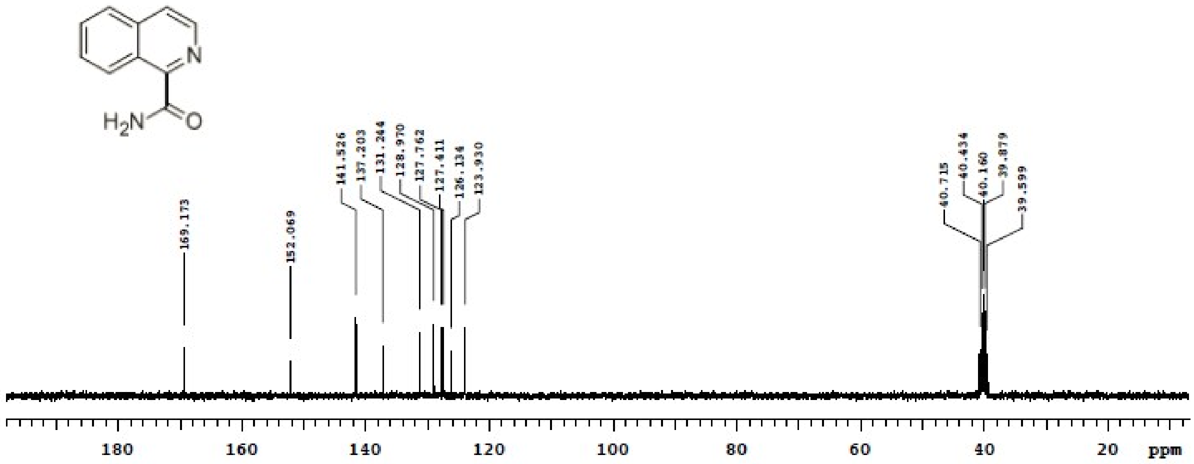
H_2N
O
N
169.173
152.069
141.526
137.203
131.244
128.970
127.762
127.411
126.134
123.930
40.715
40.434
40.160
39.879
39.599
180
160
140
120
100
80
60
40
20
ppm

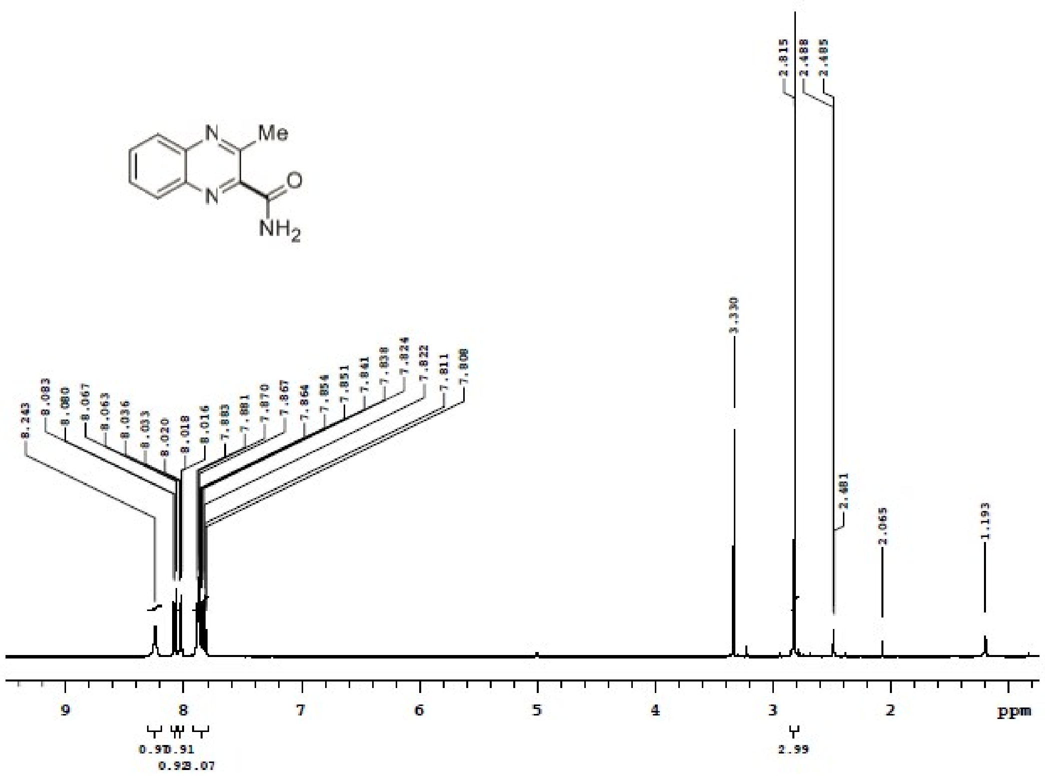
Me
N
N
O
NH2
8.243
8.083
8.080
8.067
8.063
8.036
8.033
8.020
8.018
8.016
7.883
7.881
7.870
7.867
7.864
7.854
7.851
7.841
7.838
7.824
7.822
7.811
7.808
3.330
2.815
2.488
2.485
2.481
2.065
1.193
9
8
7
6
5
4
3
2
ppm
2.99

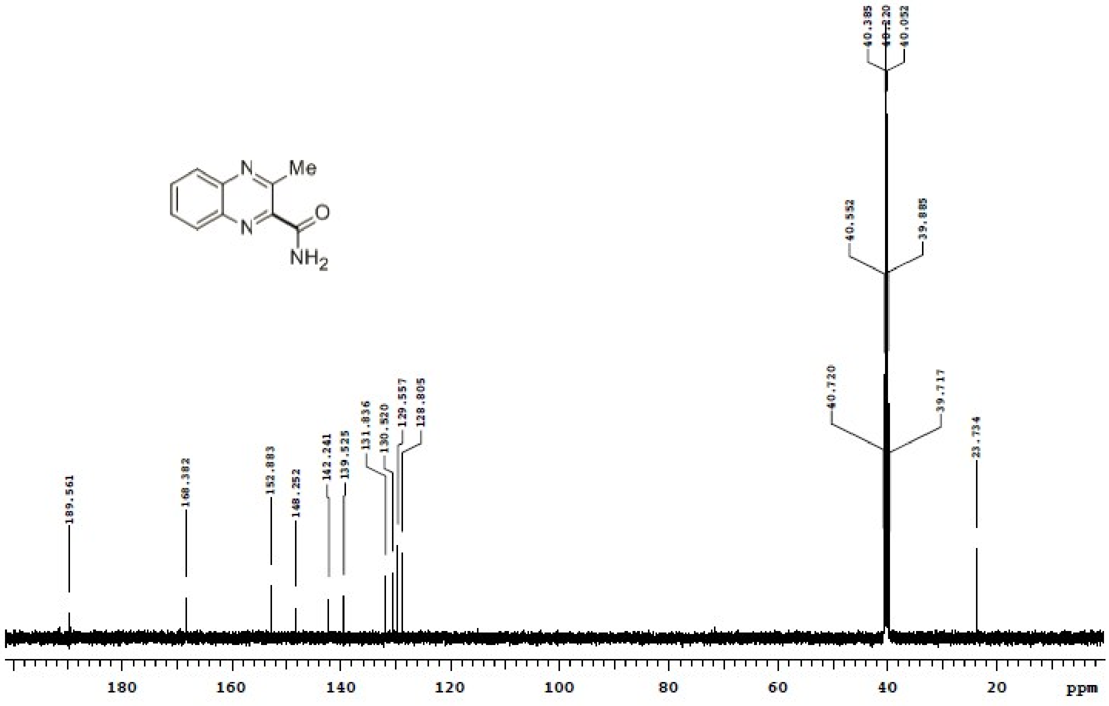
Me
N
N
O
NH2
189.561
168.382
152.883
148.252
142.241
139.525
131.836
130.520
129.557
128.805
40.385
40.052
40.552
39.885
40.720
39.717
23.734
180
160
140
120
100
80
60
40
20
ppm

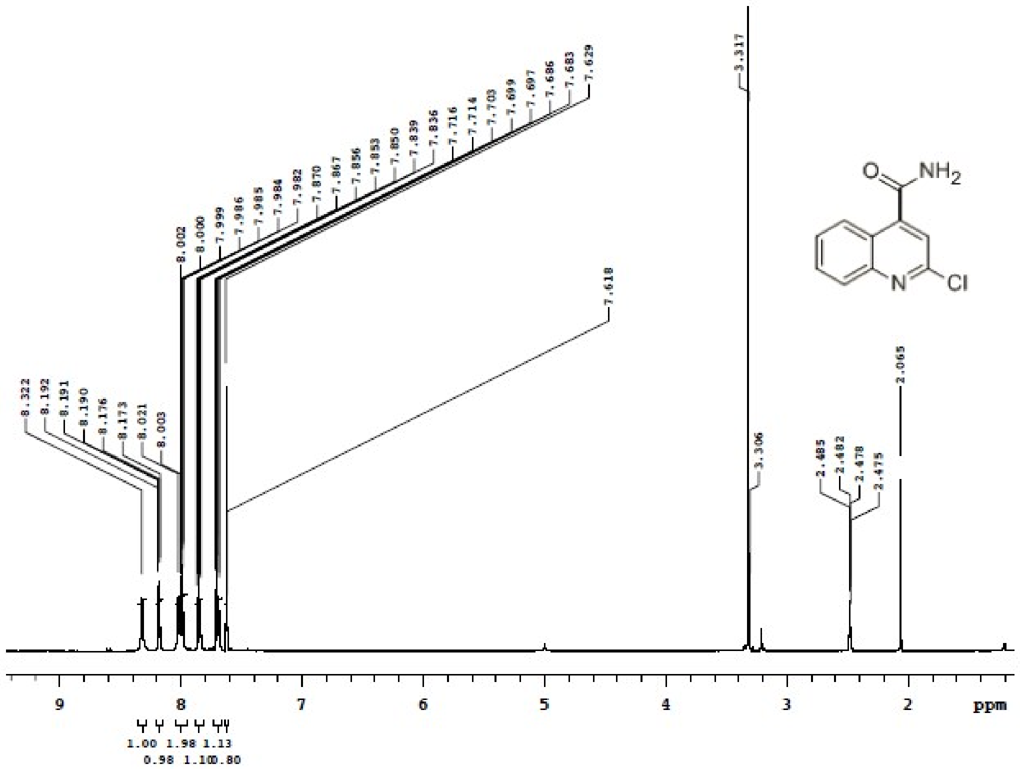

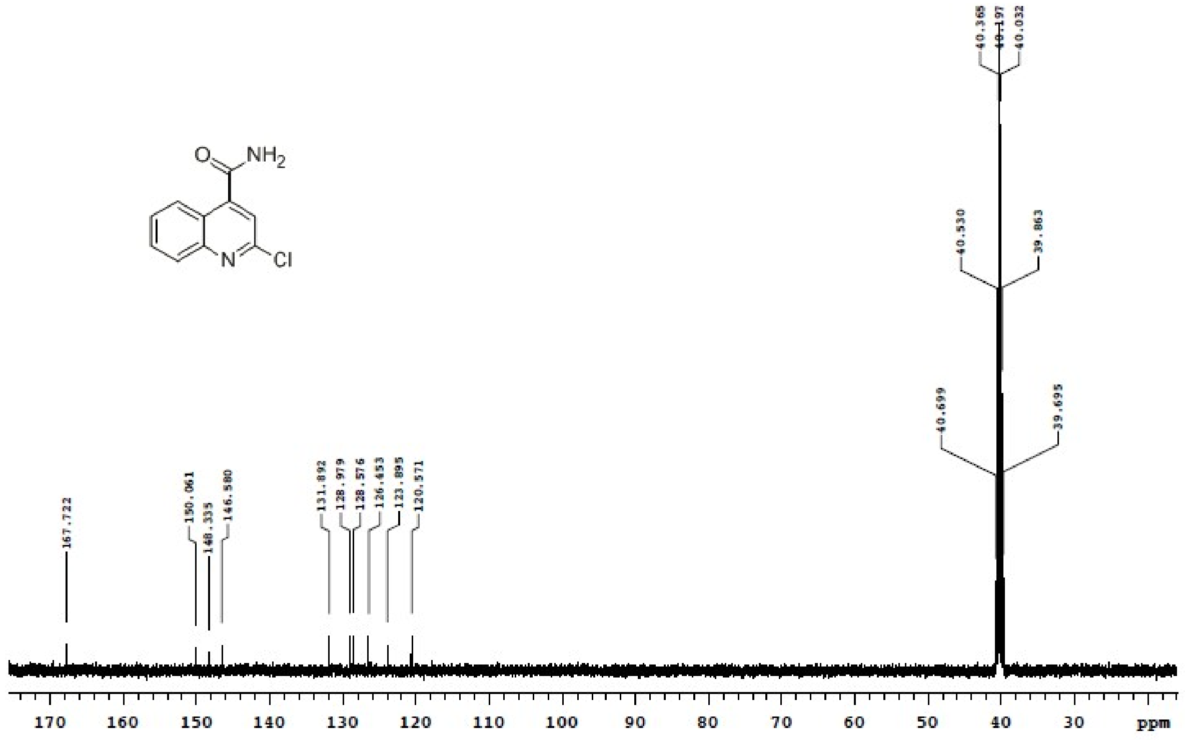

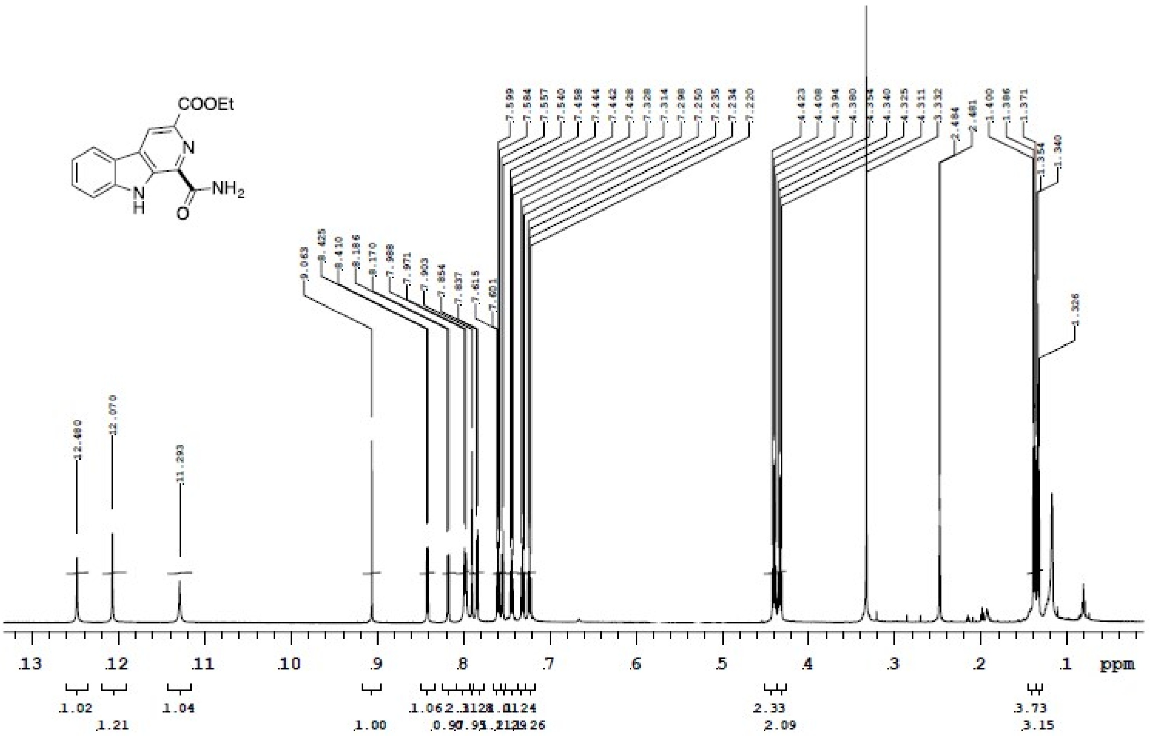
COOEt
N
NH2
N
H
O
ppm

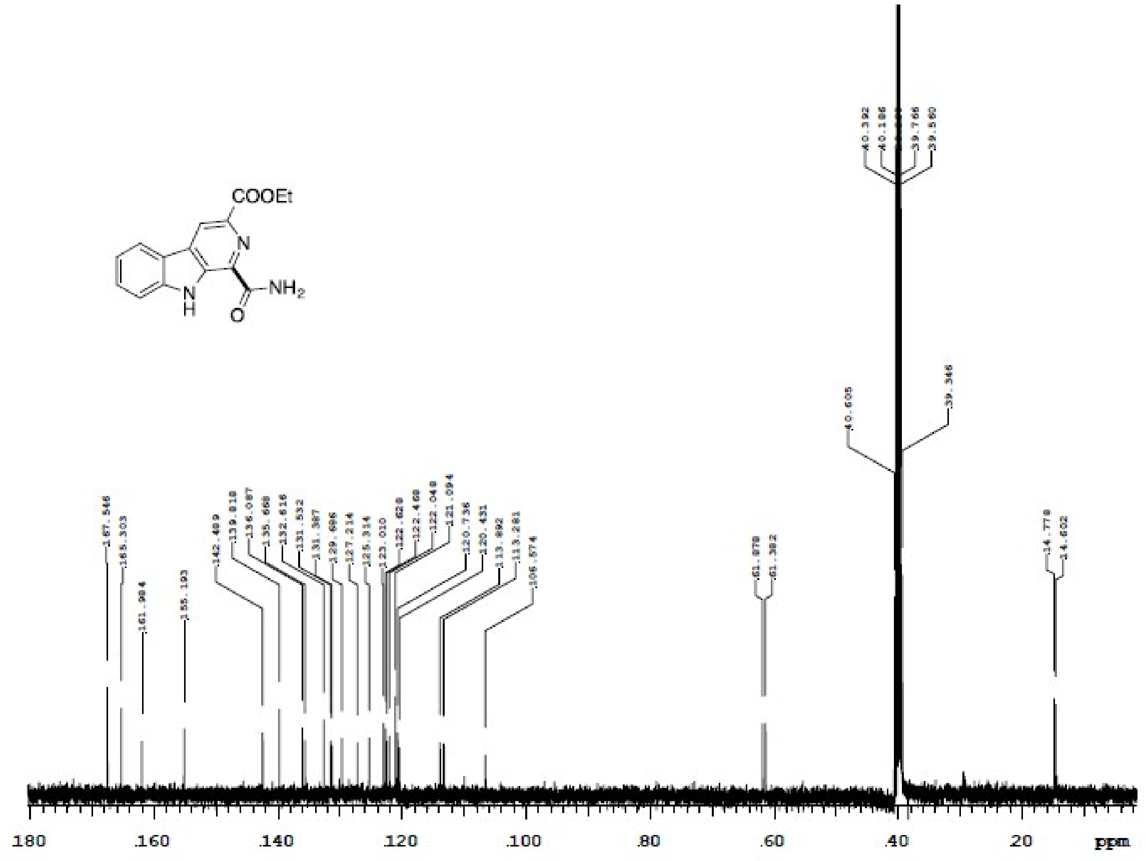
COOEt
N
NH2
N
H
O
ppm

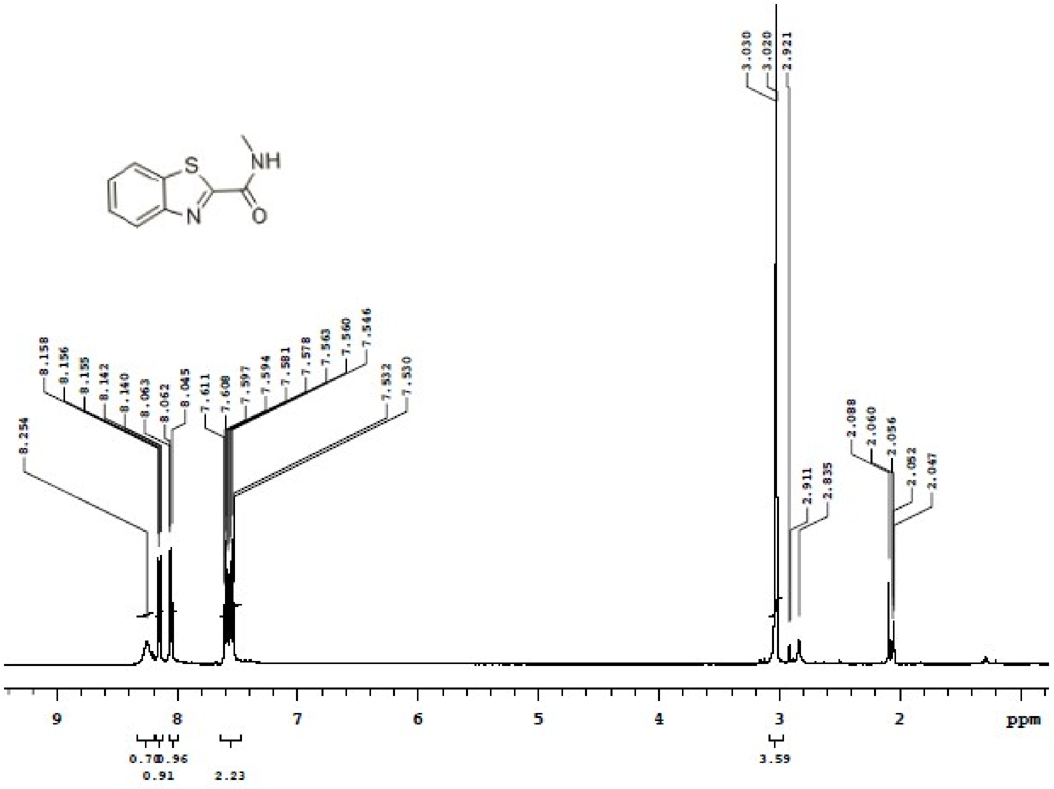
3.030
3.020
2.921
NH
S
N
O
8.158
8.156
8.155
8.142
8.140
8.063
8.062
8.045
7.611
7.608
7.597
7.594
7.581
7.578
7.563
7.560
7.546
7.532
7.530
8.254
2.088
2.060
2.056
2.052
2.047
2.911
2.835
9
8
7
6
5
4
3
2
ppm
0.70
0.96
0.91
2.23
3.59

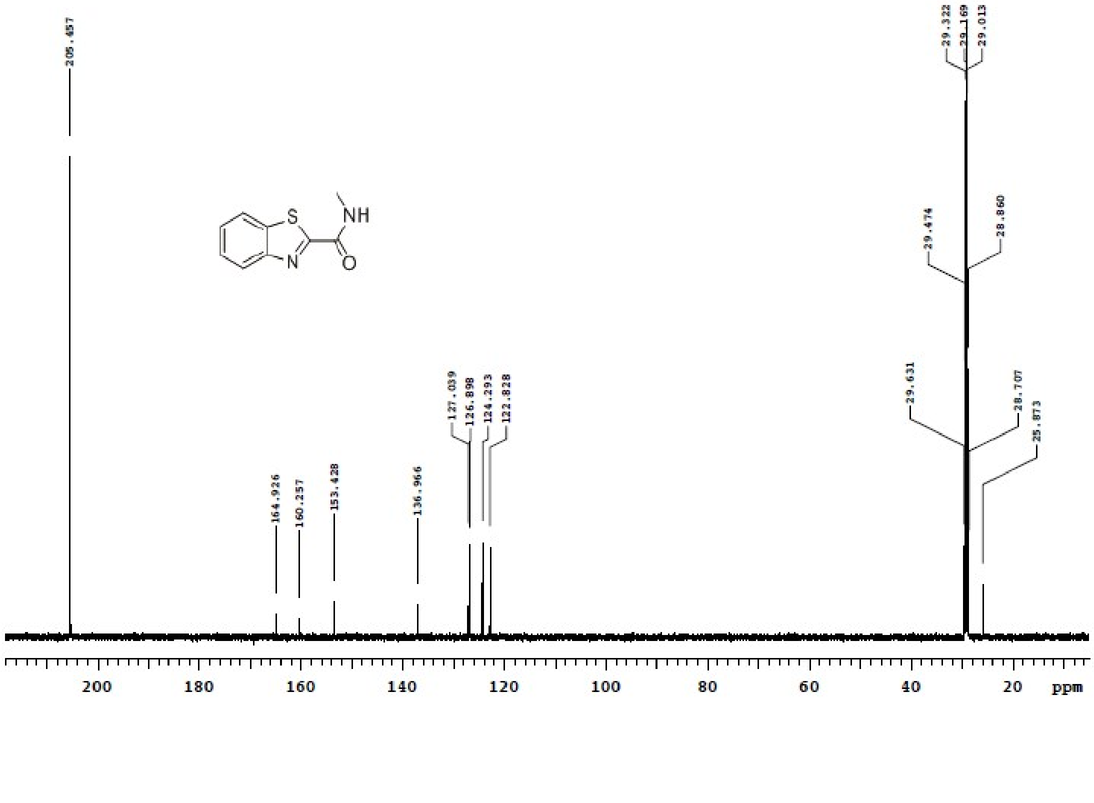
205.457
29.322
29.169
29.013
NH
S
N
O
29.474
28.860
29.631
28.707
25.873
127.039
126.898
124.293
122.828
164.926
160.257
153.428
136.966
200
180
160
140
120
100
80
60
40
20
ppm

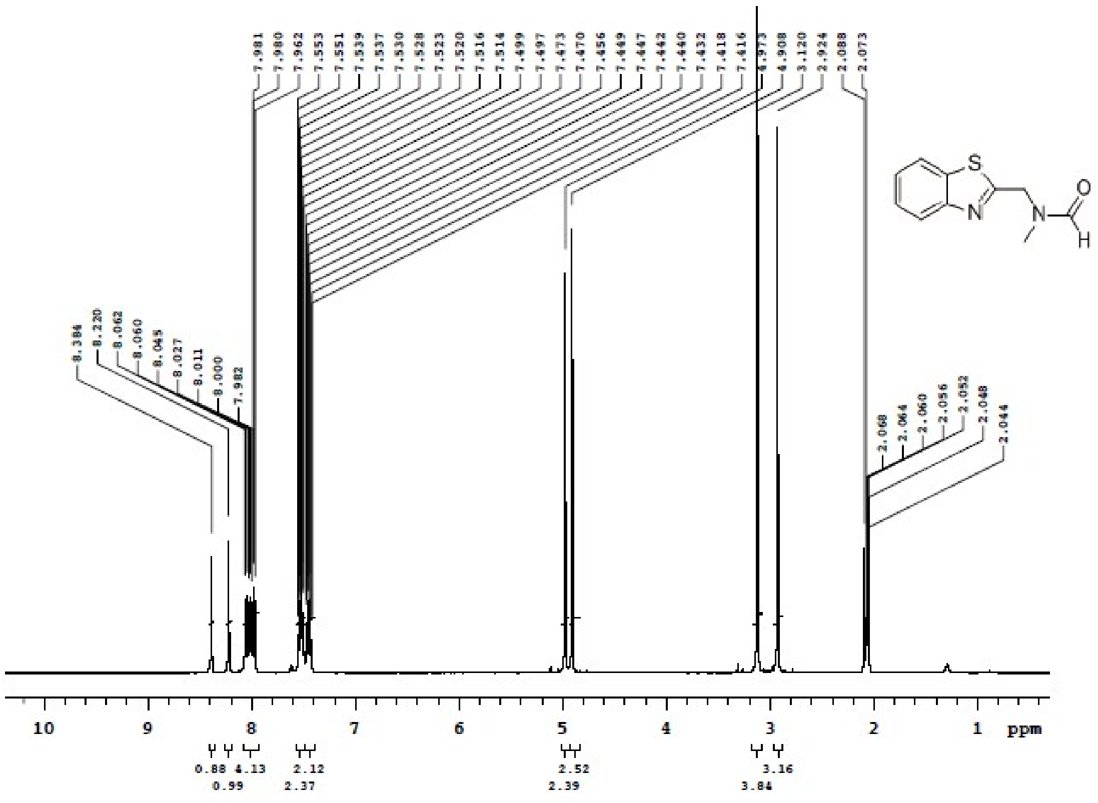

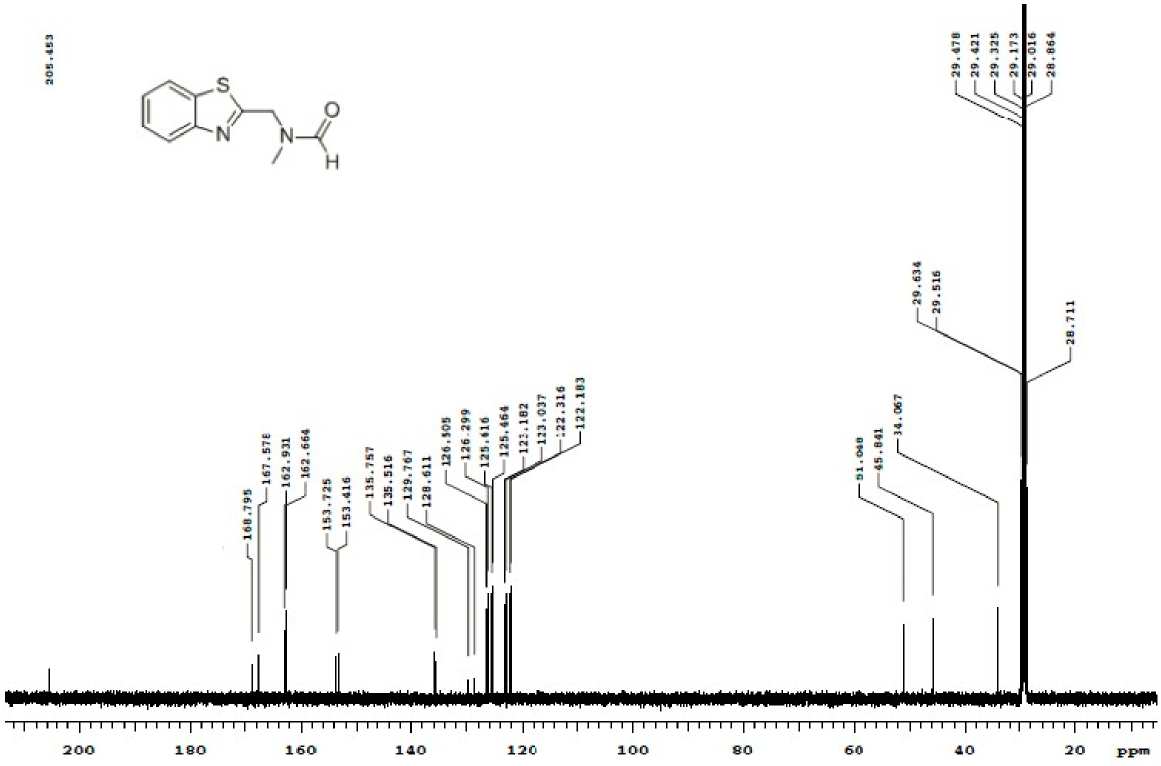

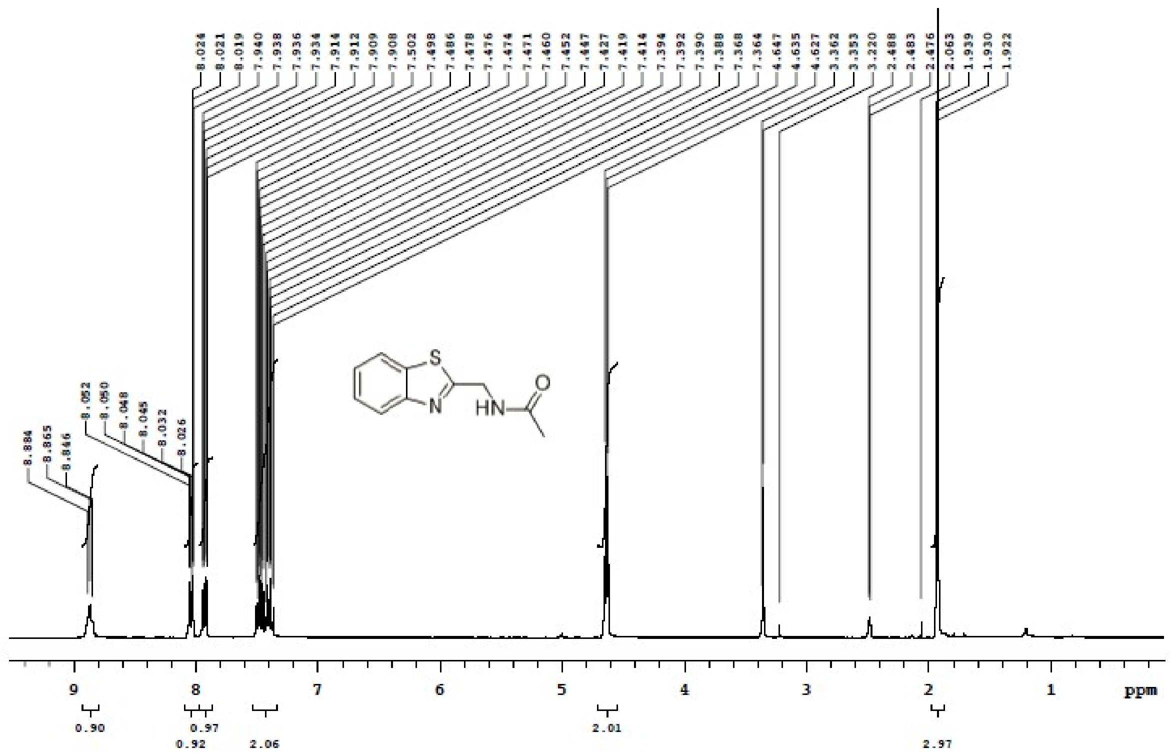
S
N
HN
O
ppm
0.90
0.92
0.97
2.06
2.01
2.97

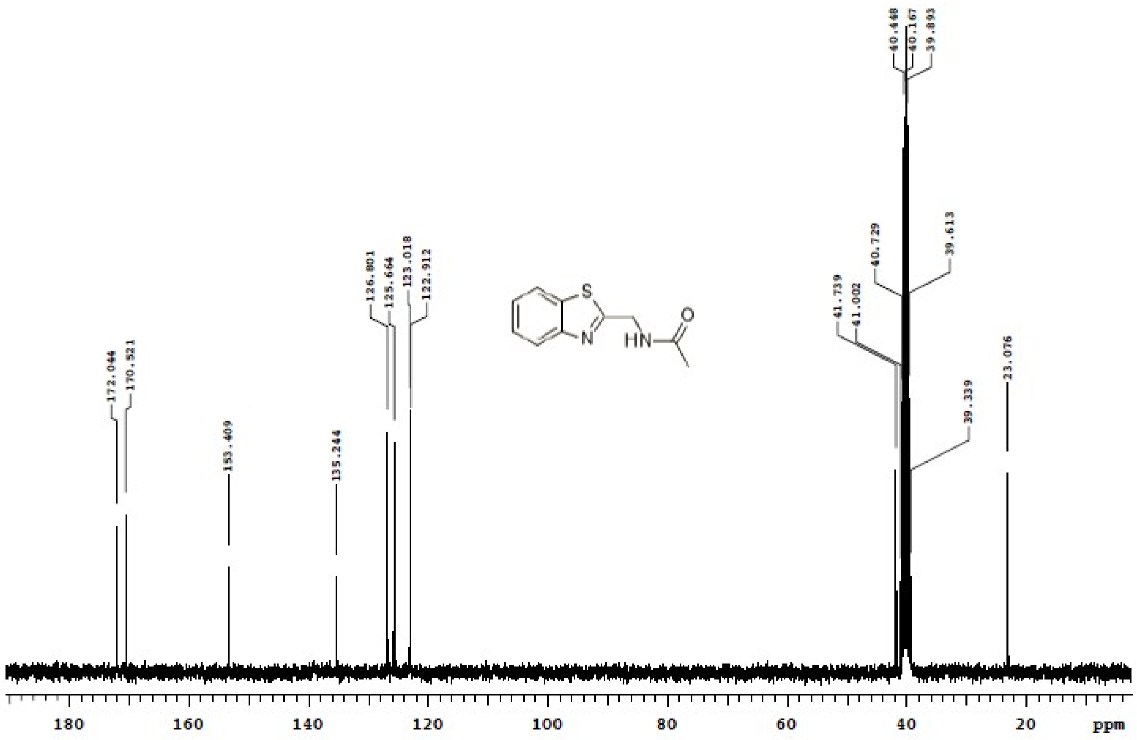
S
N
HN
O
172.044
170.521
153.409
135.244
126.801
125.664
123.018
122.912
41.739
41.002
40.729
40.448
40.167
39.893
39.613
39.339
23.076
ppm

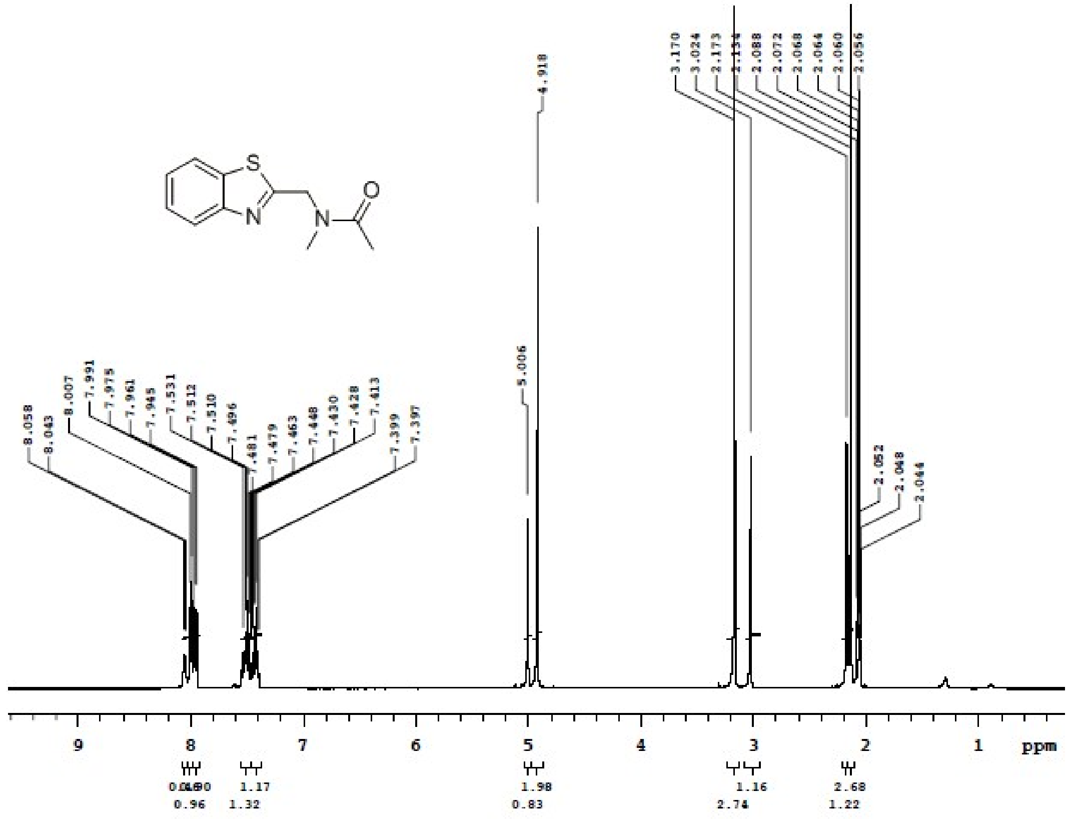

8.058
8.043
8.007
7.991
7.975
7.961
7.945
7.531
7.512
7.510
7.496
7.481
7.479
7.463
7.448
7.430
7.428
7.413
7.399
7.397
5.006
4.918
3.170
3.024
2.173
2.154
2.088
2.072
2.068
2.064
2.060
2.056
2.052
2.048
2.044
ppm
0.96
1.17
1.32
1.98
0.83
1.16
2.74
2.68
1.22

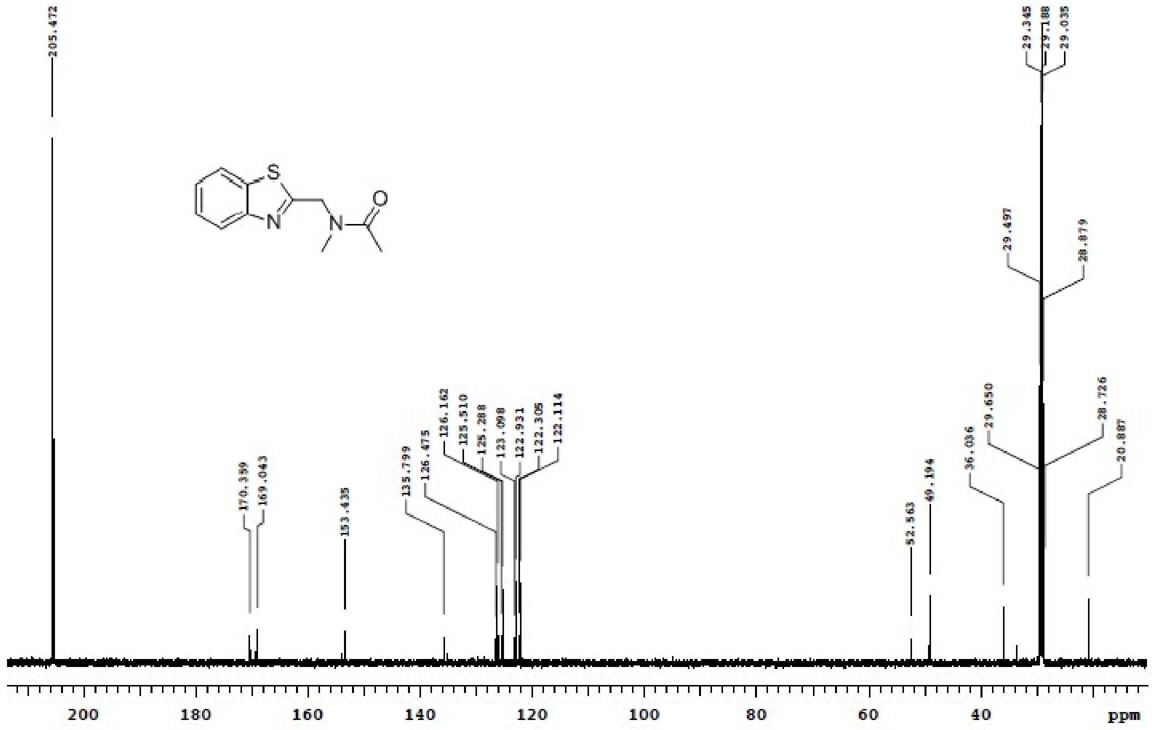

205.472
170.359
169.043
153.435
135.799
126.475
126.162
125.510
125.288
123.098
122.931
122.305
122.114
52.563
49.194
36.036
29.650
29.497
29.345
29.188
29.035
28.879
28.726
20.887
ppm

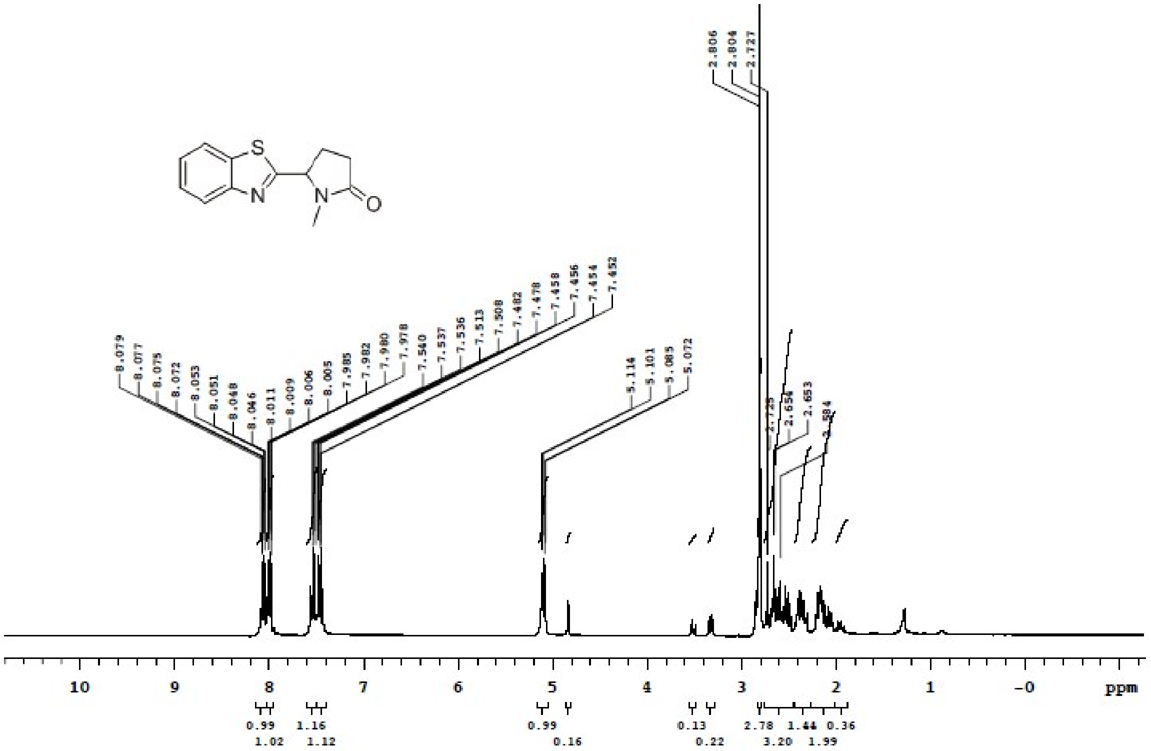

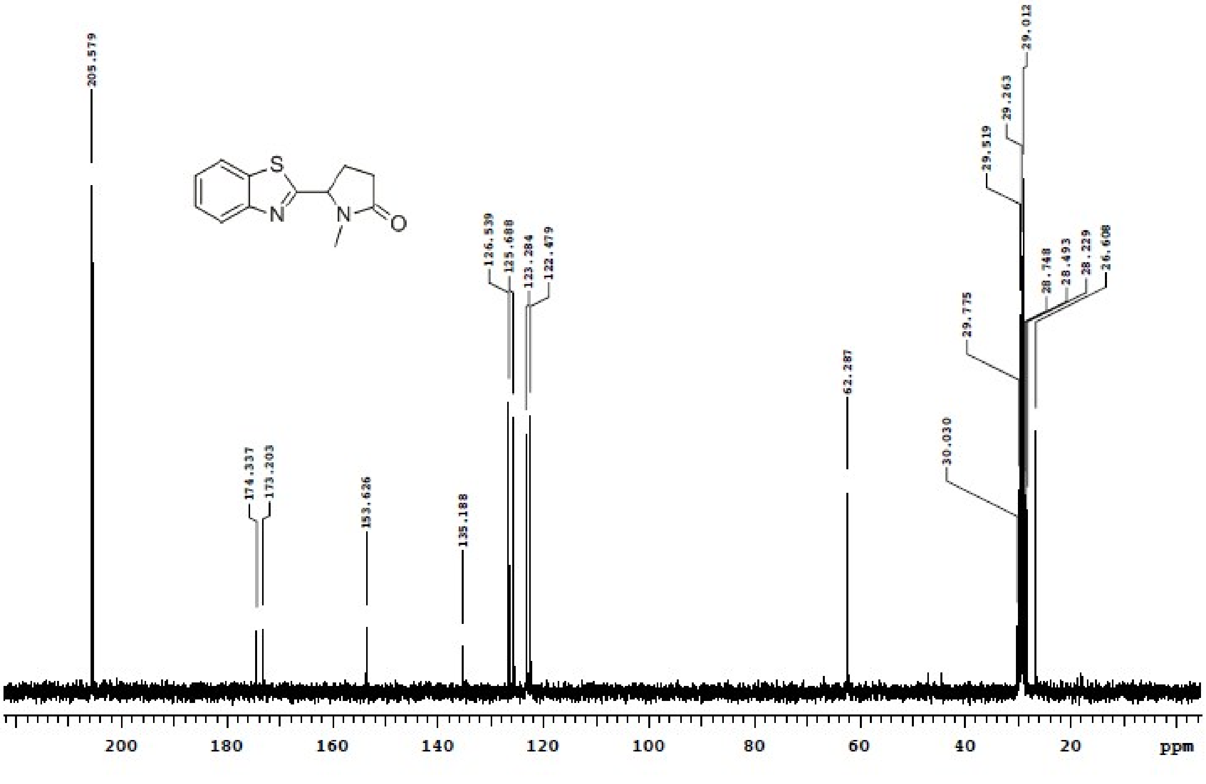

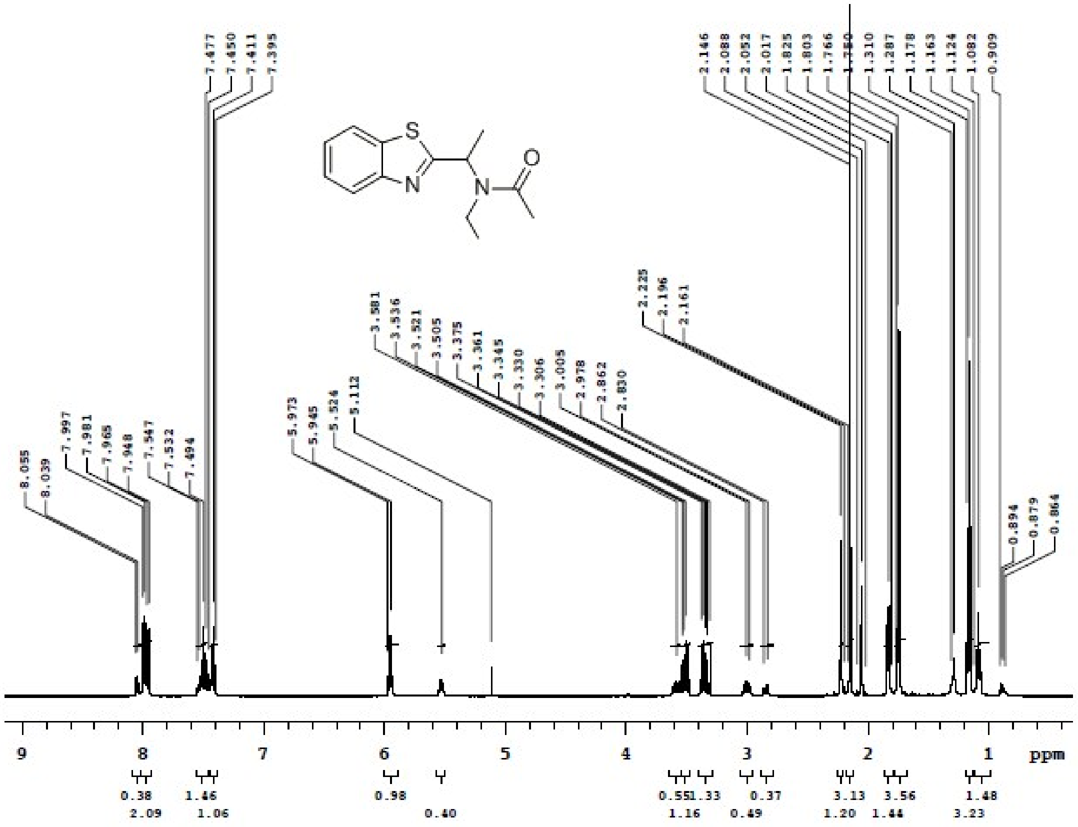
7.477
7.450
7.411
7.395
2.146
2.088
2.052
2.017
1.825
1.803
1.766
1.750
1.310
1.287
1.178
1.163
1.124
1.082
0.909
S
N
N
O
2.225
2.196
2.161
3.581
3.536
3.521
3.505
3.375
3.361
3.345
3.330
3.306
3.005
2.978
2.862
2.830
5.973
5.945
5.524
5.112
8.055
8.039
7.997
7.981
7.965
7.948
7.547
7.532
7.494
0.894
0.879
0.864
9
8
7
6
5
4
3
2
1
ppm
0.38
2.09
1.46
1.06
0.98
0.40
0.55
1.16
1.33
0.49
0.37
3.13
1.20
3.56
1.44
1.48
3.23

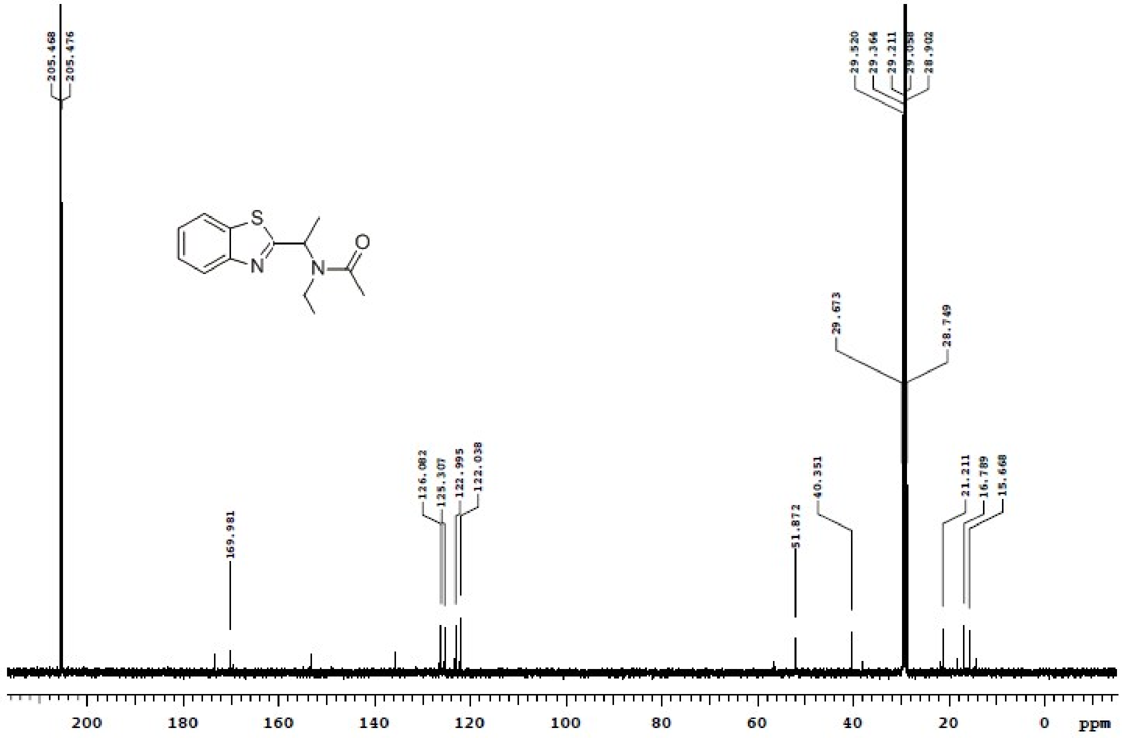
205.468
205.476
29.520
29.364
29.211
29.058
28.902
S
N
N
O
29.673
28.749
126.082
125.307
122.995
122.038
169.981
51.872
40.351
21.211
16.789
15.668
200
180
160
140
120
100
80
60
40
20
0
ppm

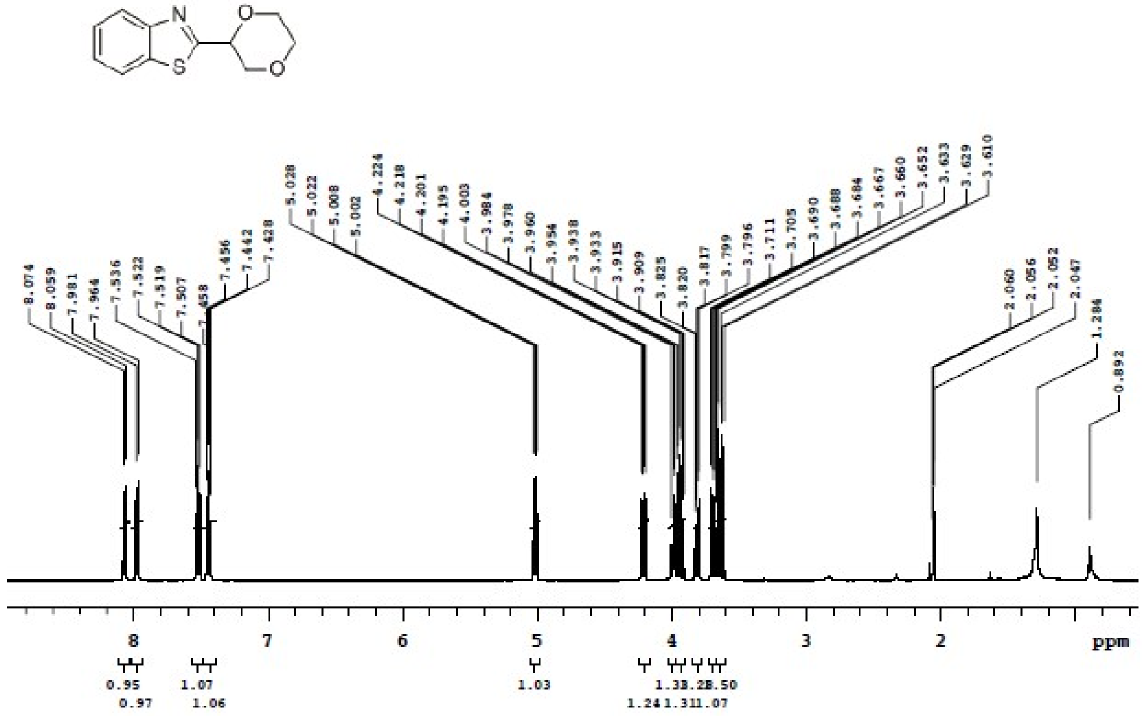
N
S
O
O
8.074
8.059
7.981
7.964
7.536
7.522
7.519
7.507
7.458
7.456
7.442
7.428
5.028
5.022
5.008
5.002
4.224
4.218
4.201
4.195
4.003
3.984
3.978
3.960
3.954
3.938
3.933
3.915
3.909
3.825
3.820
3.817
3.799
3.796
3.711
3.705
3.690
3.688
3.684
3.667
3.660
3.652
3.633
3.629
3.610
2.060
2.056
2.052
2.047
1.284
0.892
8
7
6
5
4
3
2
ppm
0.95
0.97
1.07
1.06
1.03
1.24
1.31
1.07
1.50

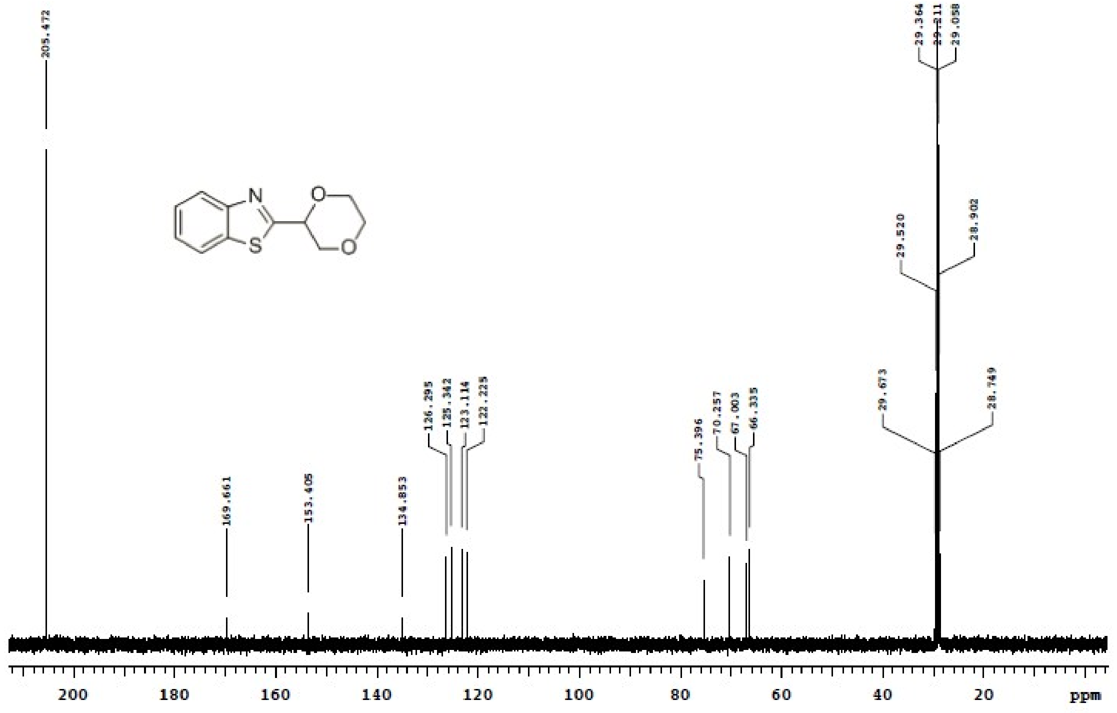
205.472
29.364
29.211
29.058
N
S
O
O
29.520
28.902
29.673
28.749
126.295
125.342
123.114
122.225
75.396
70.257
67.003
66.335
169.661
153.405
134.853
200
180
160
140
120
100
80
60
40
20
ppm

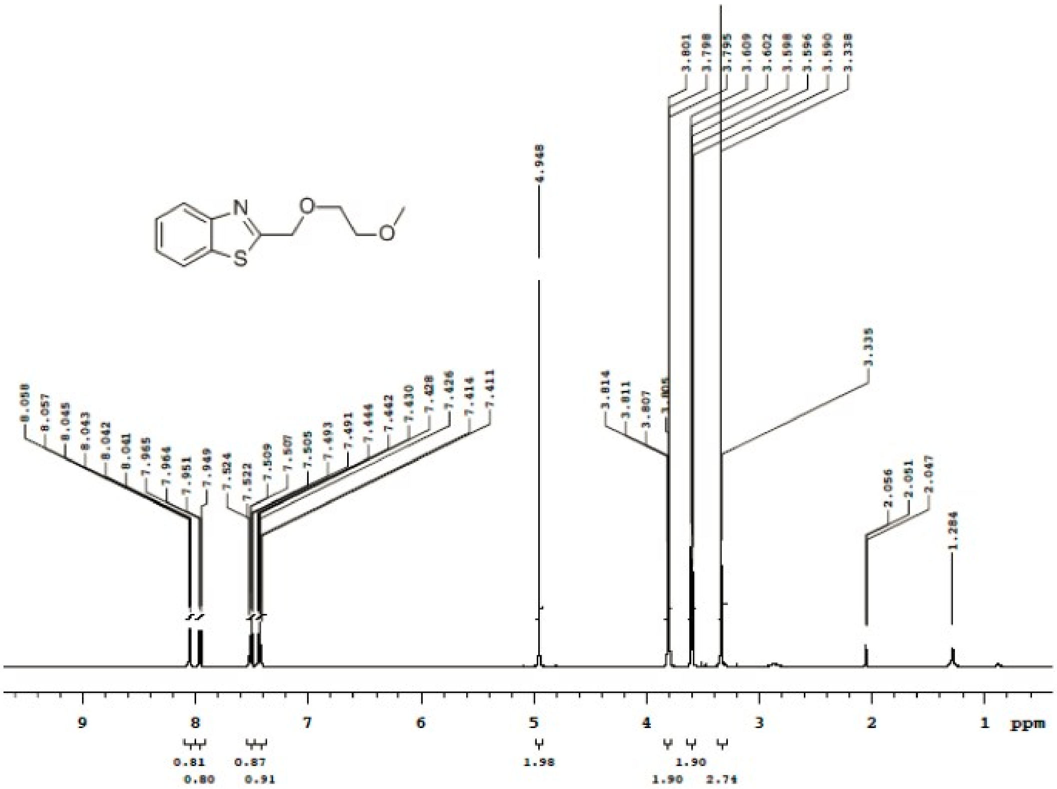
N
O
O
S
8.058
8.057
8.045
8.043
8.042
8.041
7.965
7.964
7.951
7.949
7.524
7.522
7.509
7.507
7.505
7.493
7.491
7.444
7.442
7.430
7.428
7.426
7.414
7.411
4.948
3.814
3.811
3.807
3.805
3.801
3.798
3.795
3.609
3.602
3.598
3.596
3.590
3.338
3.335
2.056
2.051
2.047
1.284
ppm
0.81
0.80
0.87
0.91
1.98
1.90
1.90
2.74

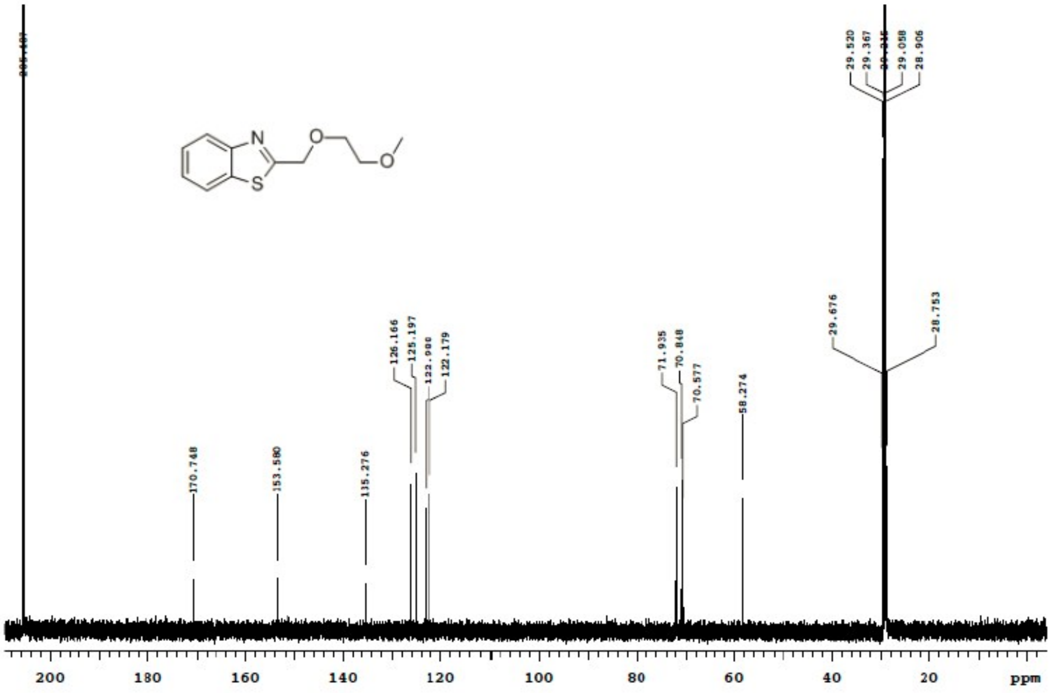
N
O
O
S
206.407
170.748
153.580
135.276
126.166
125.197
122.986
122.179
71.935
70.848
70.577
58.274
29.676
29.520
29.367
29.058
28.906
28.753
ppm

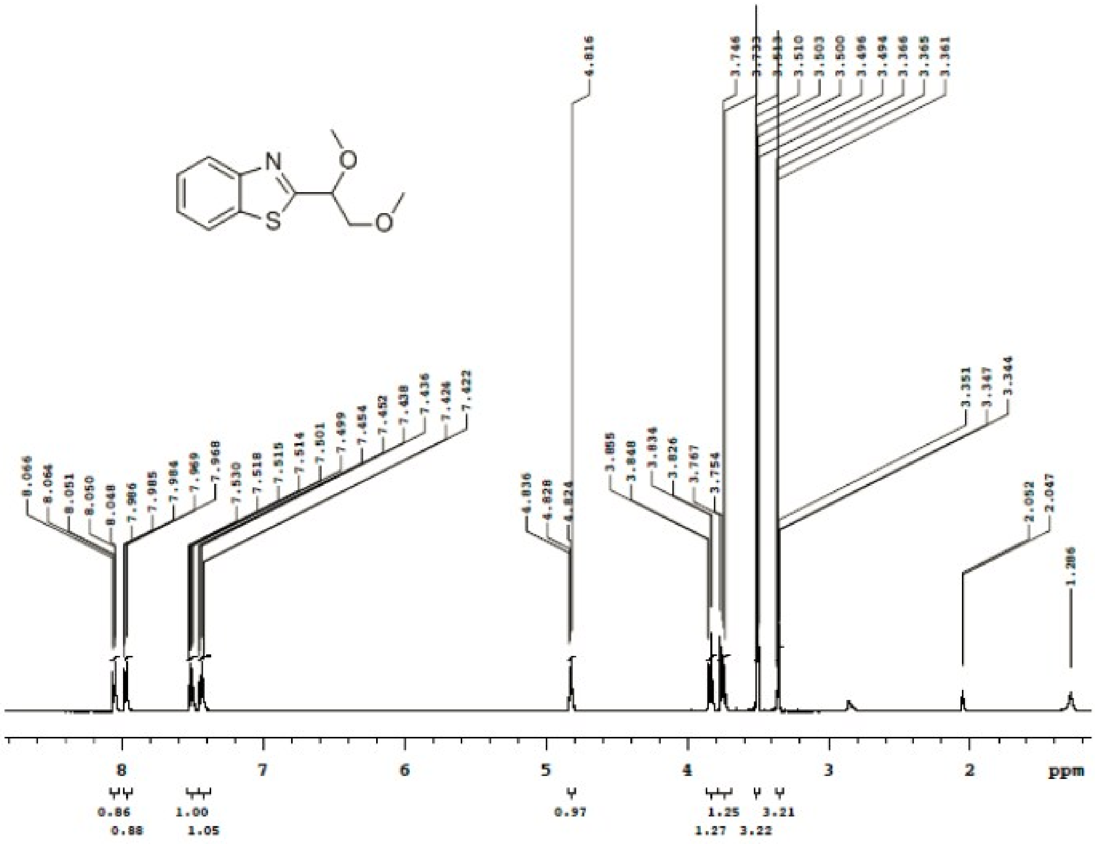
N
S
O
O
8.066
8.064
8.051
8.050
8.048
7.986
7.985
7.984
7.969
7.968
7.530
7.518
7.515
7.514
7.501
7.499
7.454
7.452
7.438
7.436
7.424
7.422
4.836
4.828
4.824
4.816
3.855
3.848
3.834
3.826
3.767
3.754
3.746
3.733
3.513
3.510
3.503
3.500
3.496
3.494
3.366
3.365
3.361
3.351
3.347
3.344
2.052
2.047
1.286
ppm
0.86
0.88
1.00
1.05
0.97
1.25
1.27
3.22
3.21

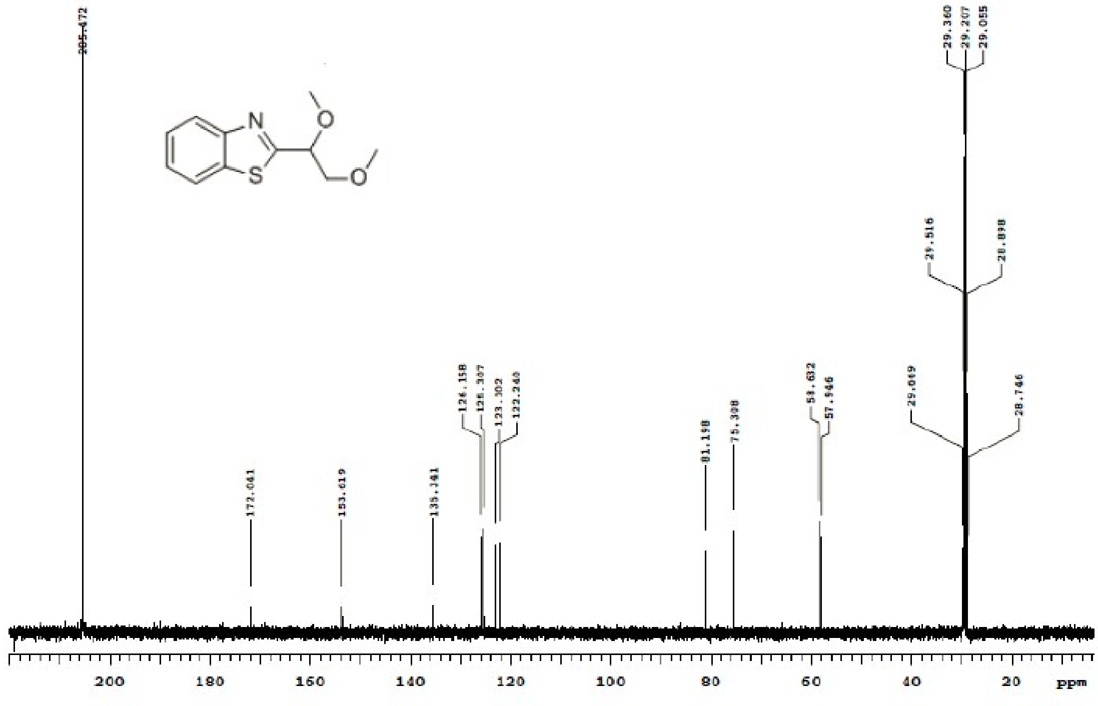
N
S
O
O
205.472
172.041
153.619
135.341
126.158
125.307
123.302
122.240
81.198
75.308
58.632
57.946
29.360
29.207
29.055
29.516
28.898
29.049
28.746
ppm

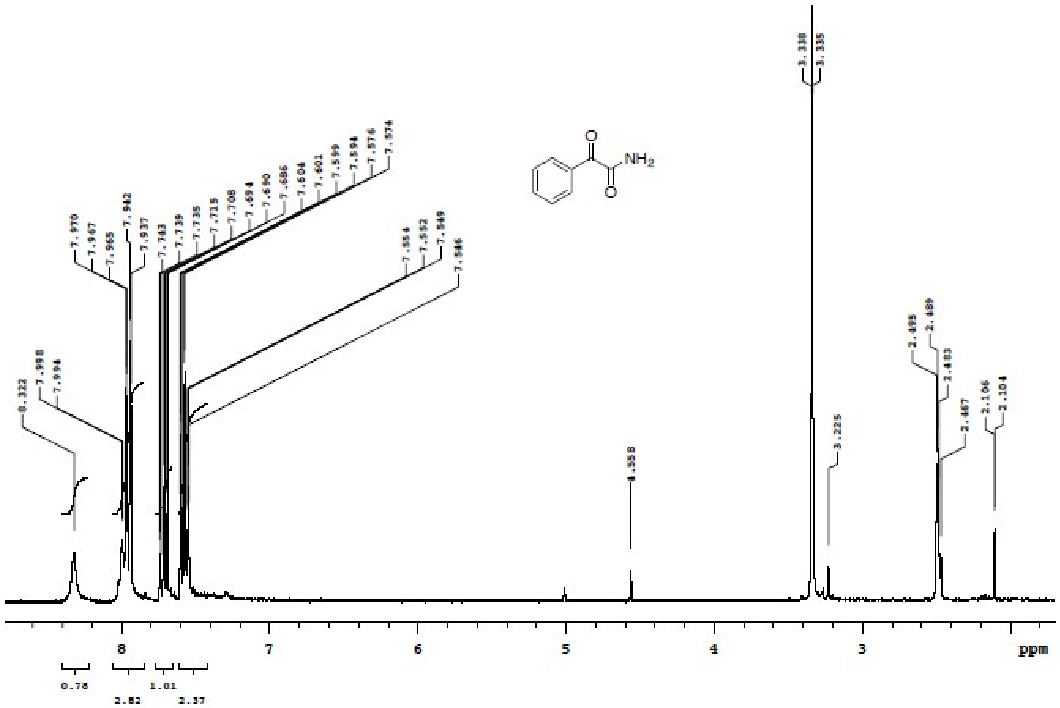

8.322
7.998
7.994
7.970
7.967
7.965
7.942
7.937
7.743
7.739
7.735
7.715
7.708
7.694
7.690
7.686
7.604
7.601
7.599
7.594
7.576
7.574
7.554
7.552
7.549
7.546
4.558
3.338
3.335
3.225
2.495
2.489
2.483
2.467
2.106
2.104
NH2
O
8
7
6
5
4
3
ppm
0.78
2.82
1.01
2.37

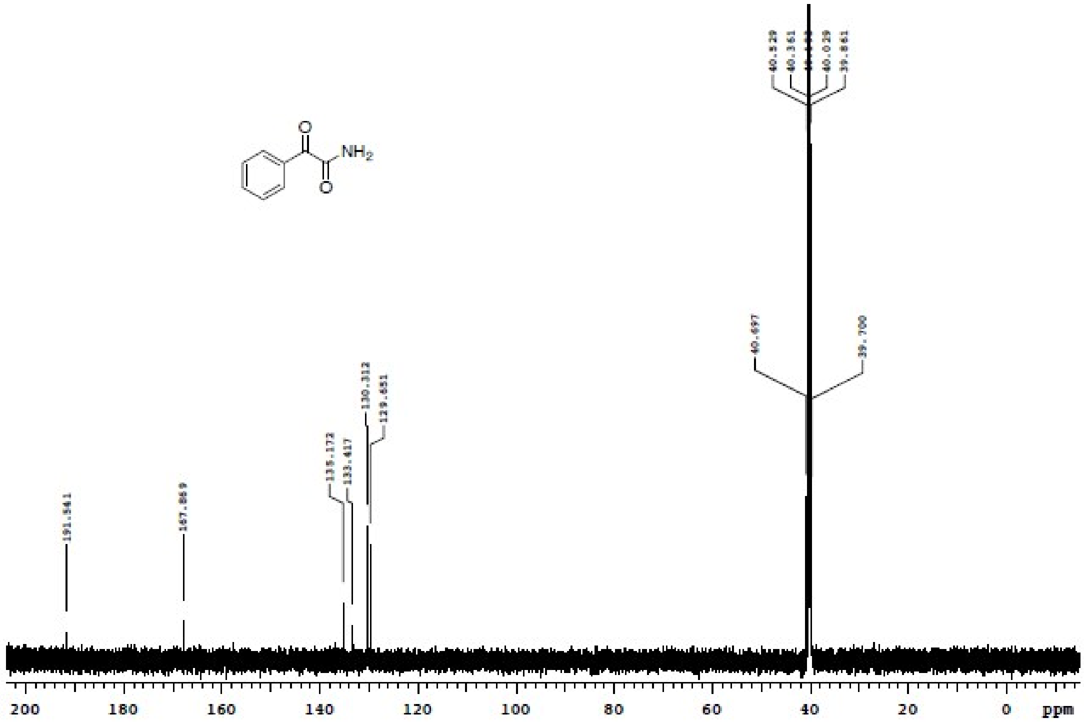

NH2
O
191.541
167.869
135.172
133.417
130.312
129.651
40.529
40.361
40.029
39.861
40.697
39.700
200
180
160
140
120
100
80
60
40
20
0
ppm

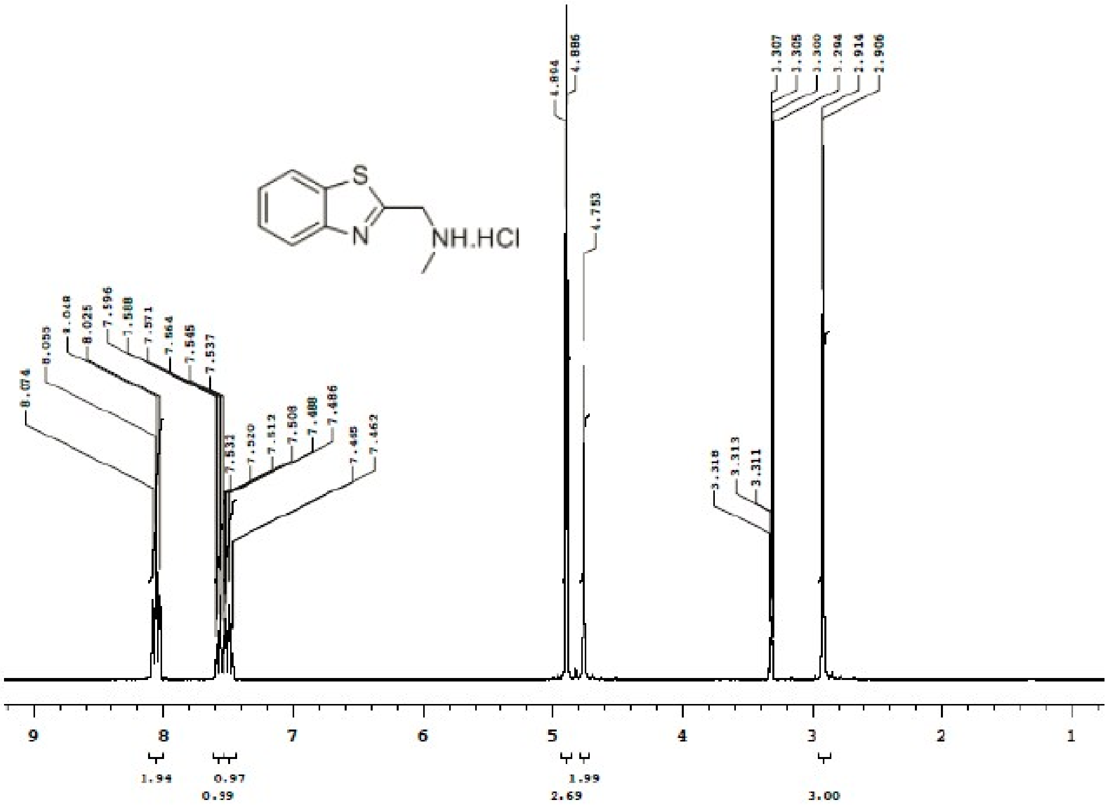
NH.HCl
S
N

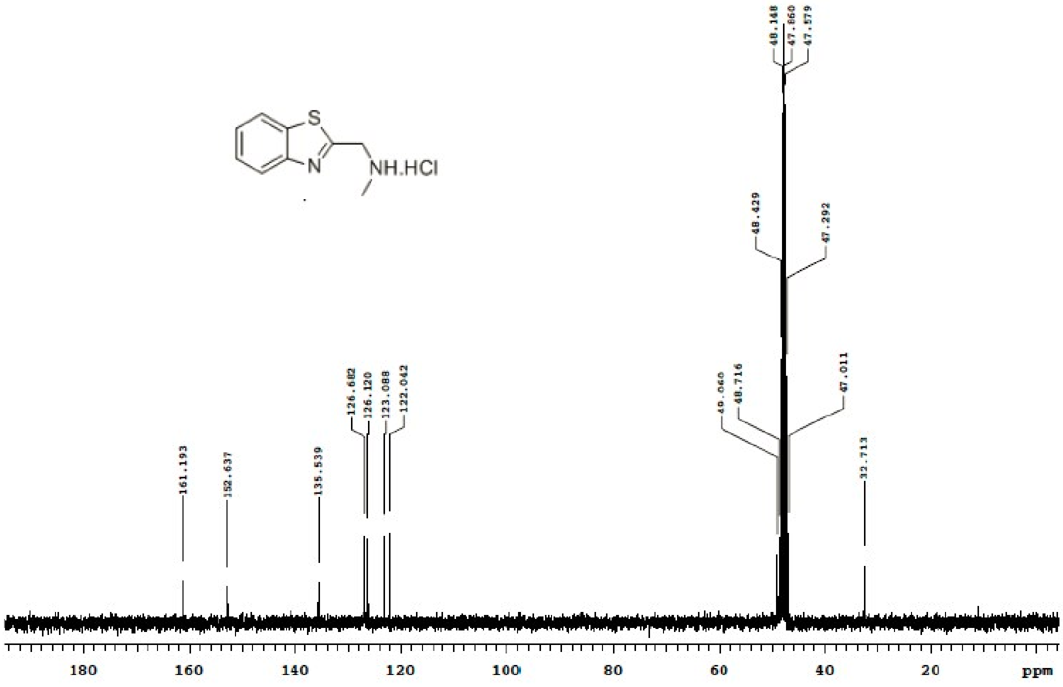
NH.HCl
S
N
ppm

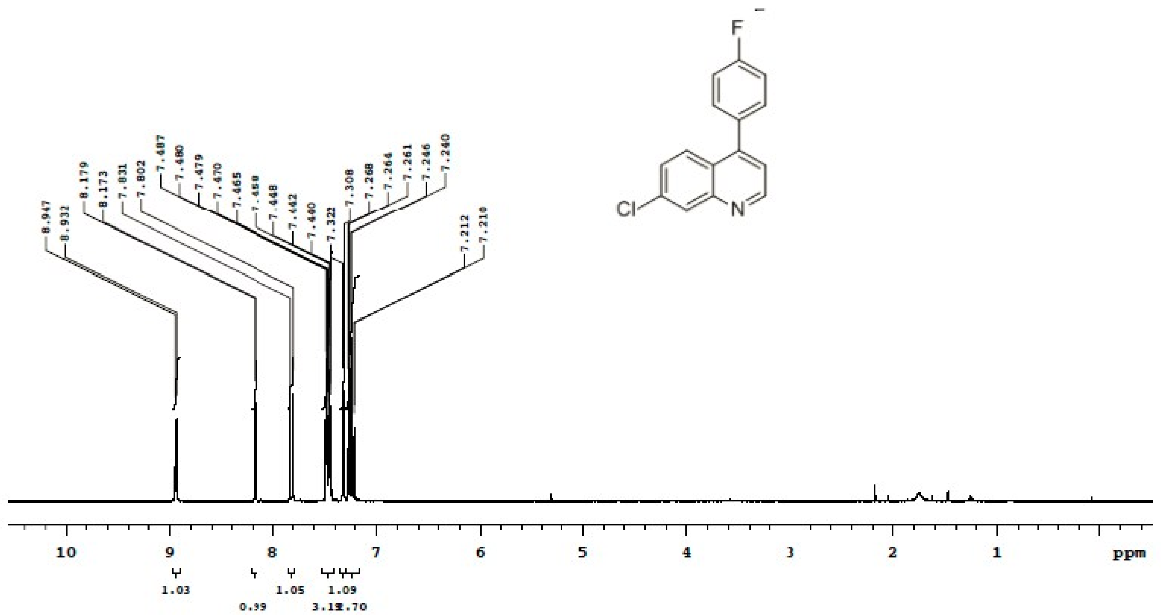

F
Cl
N
8.947
8.931
8.179
8.173
7.831
7.802
7.487
7.480
7.479
7.470
7.465
7.450
7.448
7.442
7.440
7.322
7.308
7.268
7.264
7.261
7.246
7.240
7.212
7.210
ppm
1.03
0.99
1.05
3.12
1.09
2.70

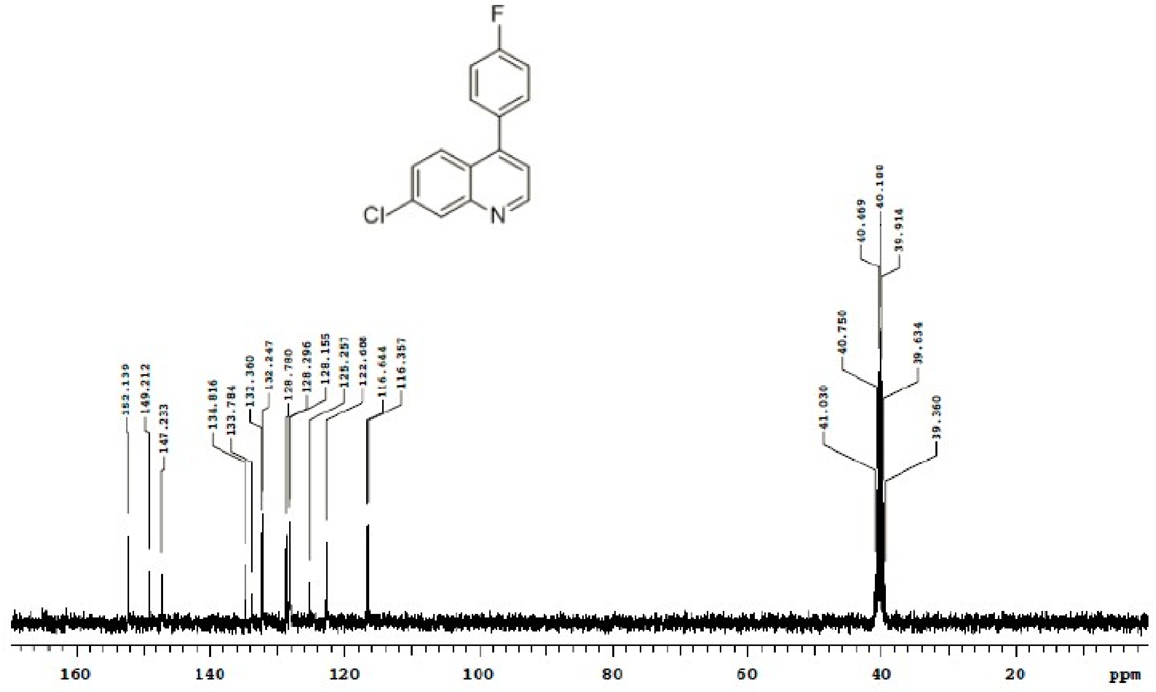

F
Cl
N
152.139
149.212
147.233
134.816
133.784
132.360
132.247
128.780
128.296
128.155
125.257
122.608
116.644
116.357
41.030
40.750
40.469
40.188
39.914
39.634
39.360
ppm

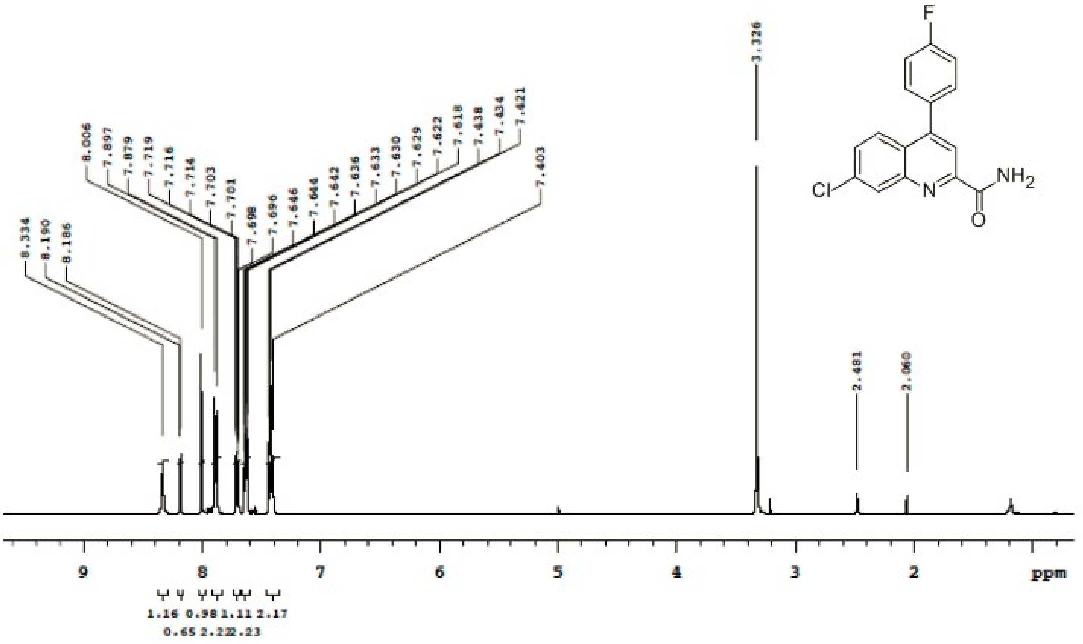

8.334
8.190
8.186
8.006
7.897
7.879
7.719
7.716
7.714
7.703
7.701
7.698
7.696
7.646
7.644
7.642
7.636
7.633
7.630
7.629
7.622
7.618
7.438
7.434
7.421
7.403
3.326
2.481
2.060
F
Cl
N
NH2
O
1.16
0.65
0.98
2.22
1.11
2.23
2.17
ppm

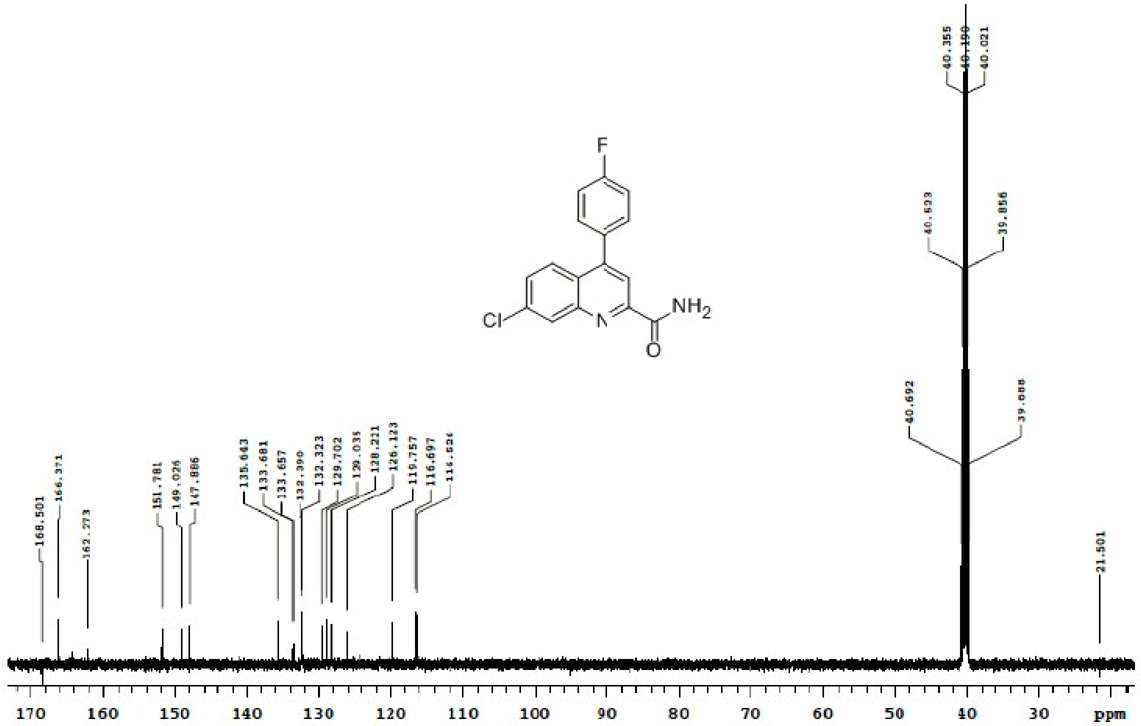

168.501
166.371
162.273
151.781
149.026
147.886
135.643
133.681
133.657
133.390
132.323
129.702
128.221
126.123
119.757
116.697
40.355
40.190
40.021
40.523
39.856
40.692
39.688
21.501
F
Cl
N
NH2
O
ppm

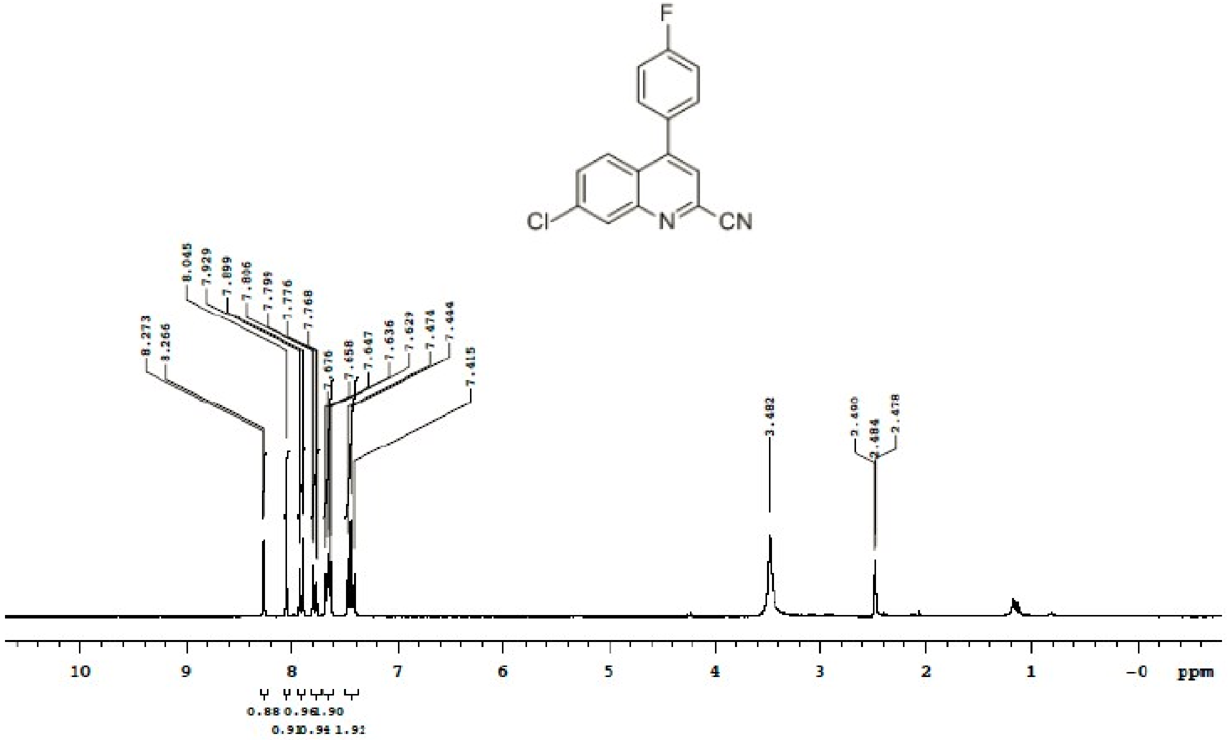
F
Cl
N
CN
8.273
8.266
8.045
7.929
7.899
7.806
7.799
7.776
7.768
7.676
7.658
7.647
7.636
7.629
7.474
7.444
7.415
3.482
2.490
2.484
2.478
10
9
8
7
6
5
4
3
2
1
-0
ppm
0.88
0.96
1.90
0.91
0.94
1.92

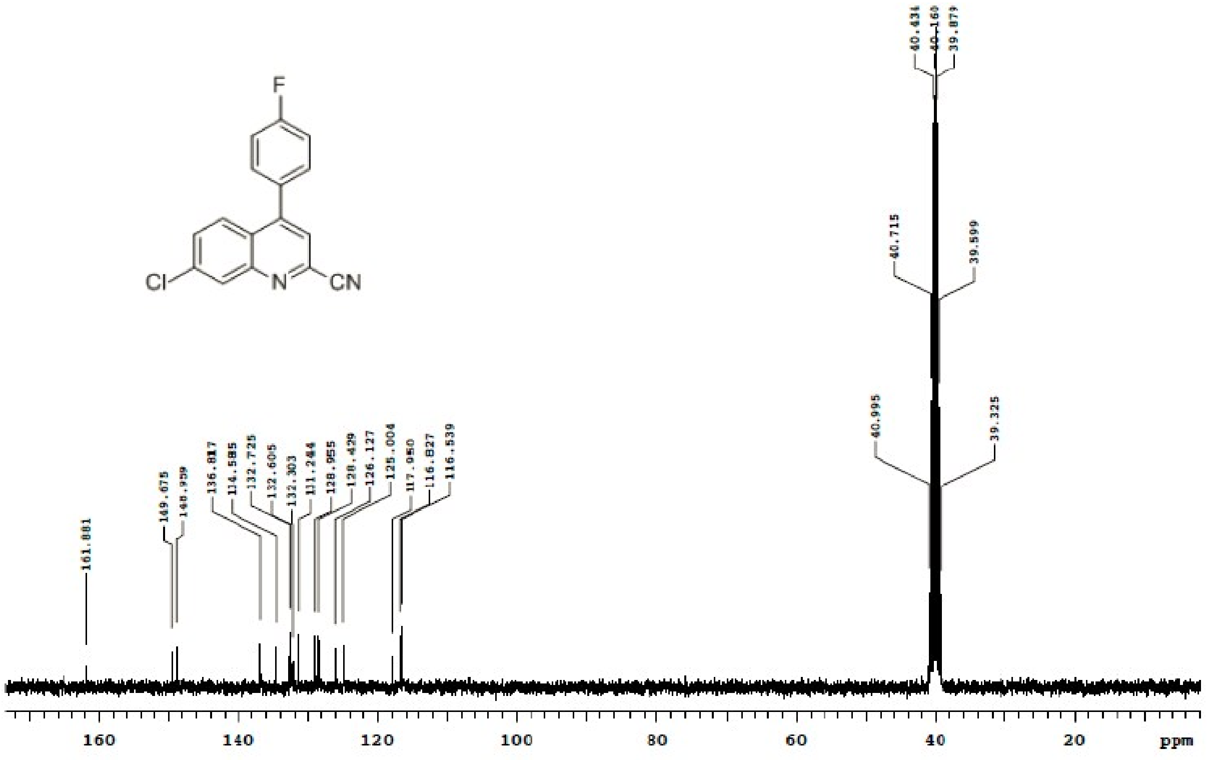
F
Cl
N
CN
161.881
149.675
148.959
136.817
144.585
132.725
132.605
132.303
131.244
128.955
128.429
126.127
125.004
117.950
116.827
116.539
40.434
40.160
39.879
40.715
39.599
40.995
39.325
160
140
120
100
80
60
40
20
ppm

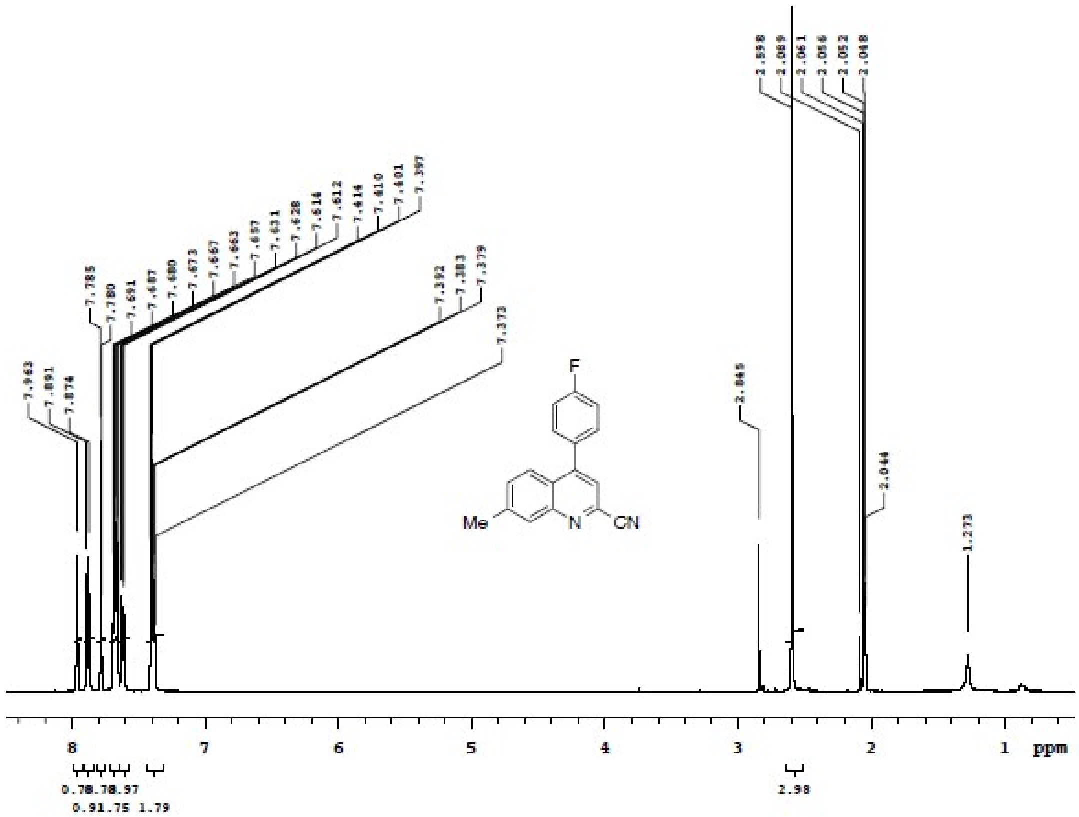

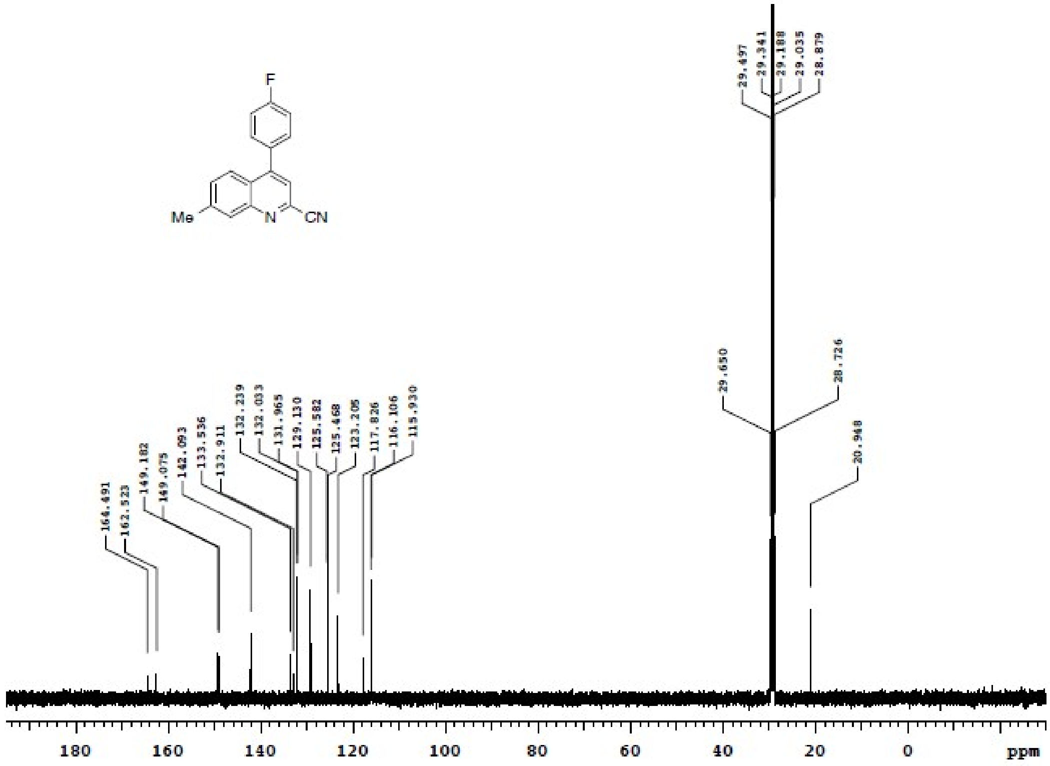

Supplement: Supplementary file 1 [file SC-007-C5SC03640B-s001.pdf]
